# Supplementary material for: Cran1, member of a new class of OLD family ATPases, functions in cell cycle progression in an archaeon
Source: EMBO Rep. 2025 Dec 2;27(1):208–29. doi: 10.1038/s44319-025-00650-y (PMC12796447; doi:10.1038/s44319-025-00650-y)
Supplement: Supplementary file 1 — Appendix [file 44319_2025_650_MOESM1_ESM.pdf]

Appendix: Table of Contents

Appendix	Page
Appendix Figure S1	2
Appendix Figure S2	3
Appendix Figure S3	4
Appendix Figure S4	5
Appendix Figure S5	6
Appendix Table S1	7–10
Appendix Table S2	11–14

Appendix Figures

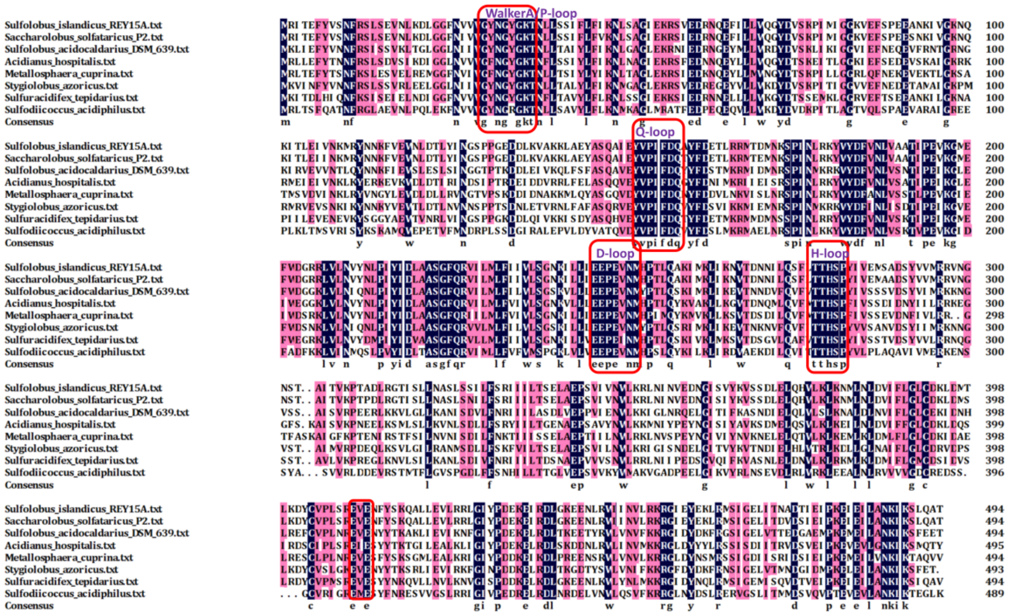

Appendix Figure S1. The Cran1 is highly conserved in Sulfolobales. Multiple sequence alignment of Cran1 homologs from Sulfolobales by DNAMAN software. The names of the species and strain where the homolog sequences were retrieved from the geneomes are indicated on the left. The protein sequences including Cran1 from S. islandicus REY15A (SiRe_0086), Sulfolobus acidocaldarius DSM639 (Saci_0157), Saccharolobus solfataricus,Acidianus, Metallosphaera cuprina, Sulfuracidifex tepidarius, Stygiolobus azoricus, Thermoprotei archaeon were retrieved from NCBI database after BLAST/PSI-BLAST analysis using SiRe_0086 as a query sequence.The red boxes are labelled with predicted conserved ATPase active sites, including P-loop,Q-loop,D-loop,H-loop.

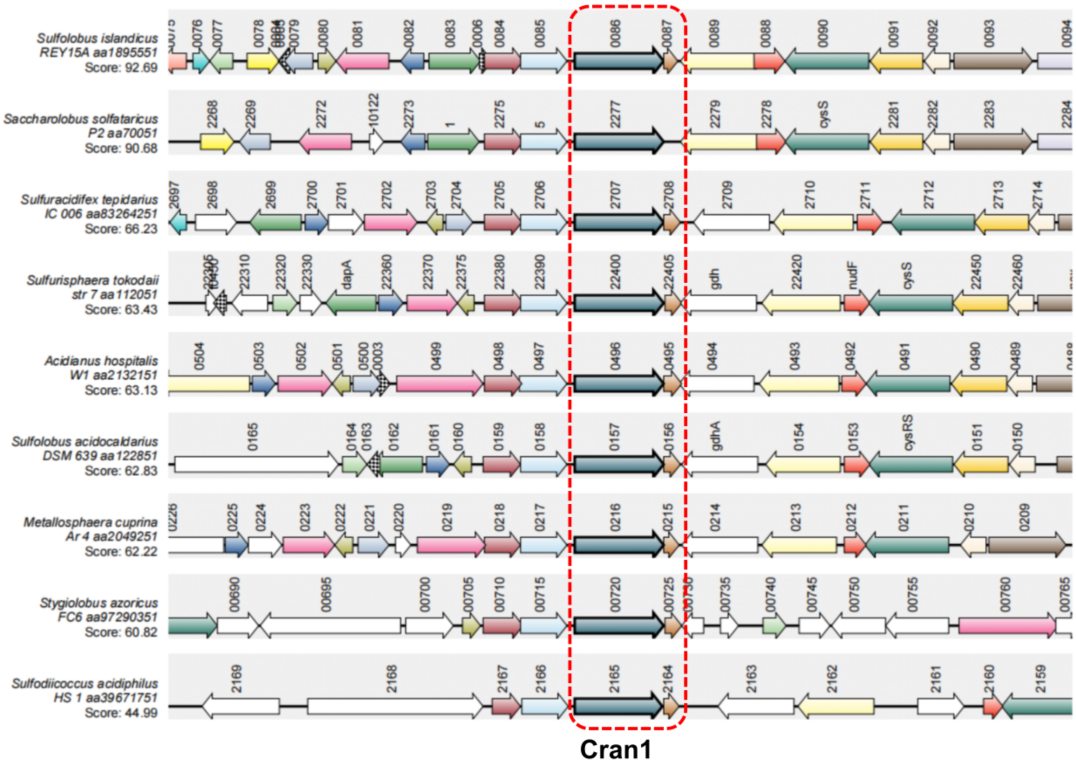

Appendix Figure S2. Gene synteny analysis of Cran1 proteins in representative species. The amino acid sequence of Cran1 (SiRe_0086) from *S. islandicus* REY15A was used as the query for the analysis by the SyntTax (https://archaea.i2bc.paris-saclay.fr/SyntTax/). The homologous genes corresponding to the query proteins are indicated with bold frames. The scores below the microbial names on the left indicate the normalized blast score between the query sequence and the orthologues in the matched chromosome.

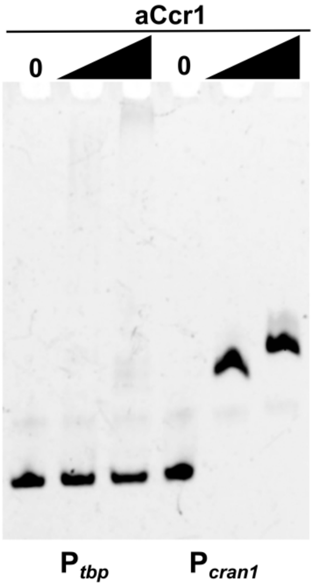

Appendix Figure S3. aCcr1 binds to the promoter of *cran1*. EMSA of aCcr1 binding to the promoter of *cran1* and *tbp* was shown. The 5’ FAM-labelled and the corresponding complementary nucleotide sequences are listed in Appendix Table S2. The reaction was performed at 37°C for 30 min and analysed on a 10% native PAGE (see ‘Materials and Methods’). Each reaction contained 2 nM of the FAM-labelled probe and 0, 0.5 and 1 μM aCcr1 protein.

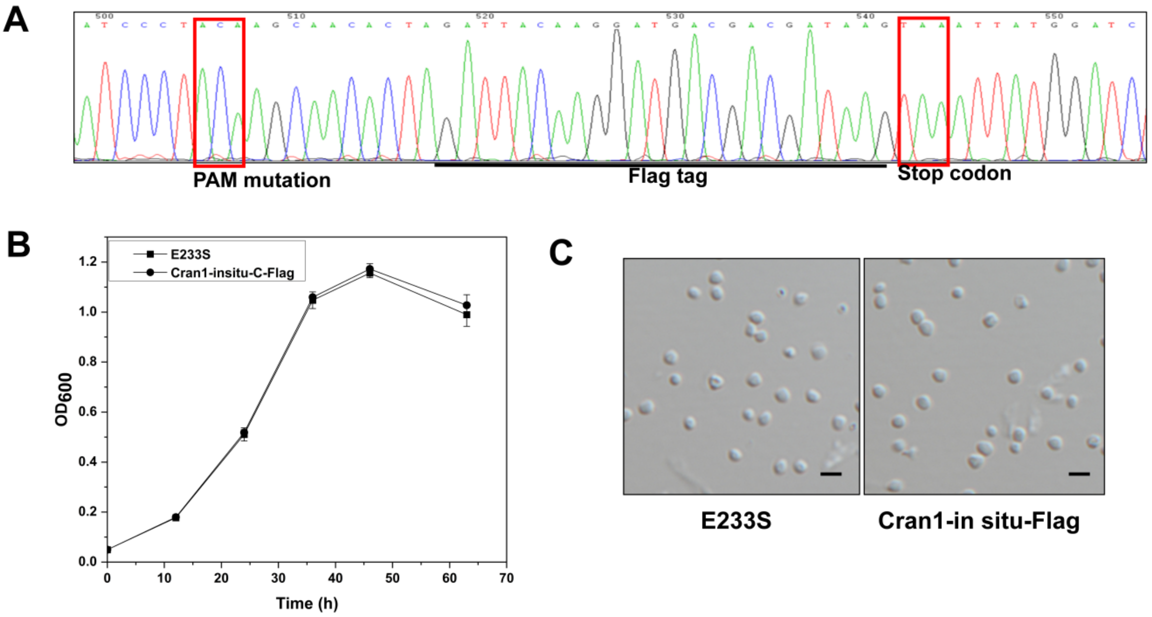

Appendix Figure S4. The growth of the *in situ* Flag-tagged Cran1 strain is not affected. (**A**) Verification of the *in situ* Flag-tagged strains by sequencing. The red boxes indicate the mutated PAM sequence and the stop codon, respectively. Comparison of the growth (**B**) and cell morphology (**C**) of the *in situ* Flag-tagged strain and the control E233S.

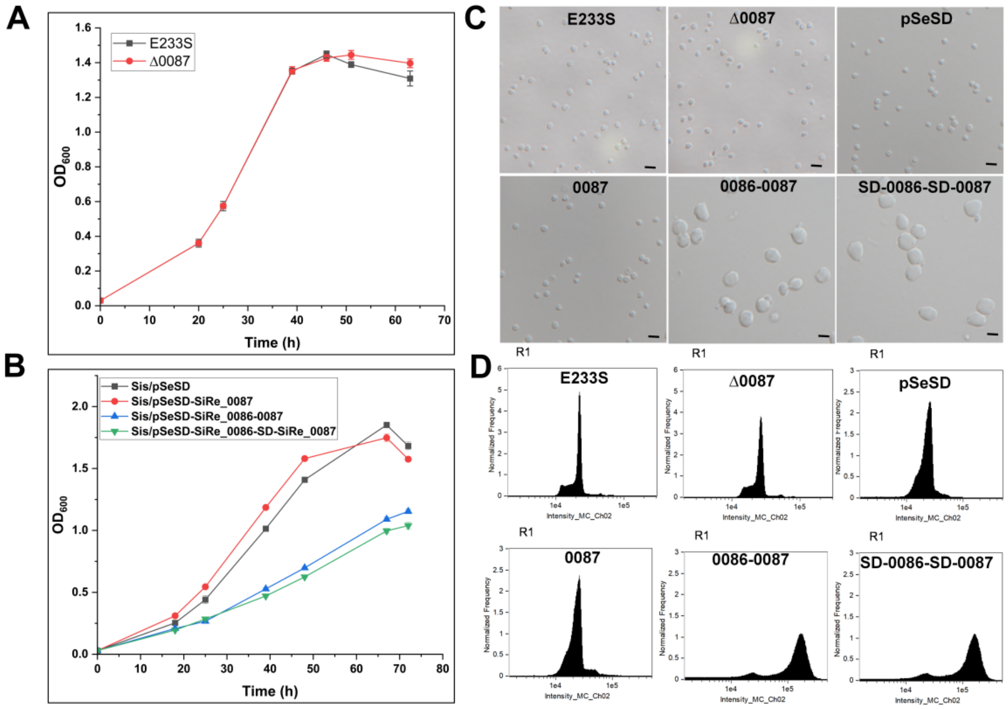

Appendix Figure S5. Phenotypic analysis of SiRe_0087 knockout strains and Cran1 and SiRe_0087 co-expression strains. (**A**) Growth curves of SiRe_0087 knockout strain and wild-type strain E233S were determined in MTSVU medium. Initial OD_{600}=0.03 (**B**) Overexpression of SiRe_0087 alone, co-expression of Cran1 and SiRe_0087 (sharing an arabinose promoter as well as using the arabinose promoter separately) strains were determined by growth curves in MTAV medium with an initial OD_{600}=0.03. (**C**) Observation of cellular morphology of the strains in A and B at 24h of growth and flow cytometry analysis (**D**) Scale bars: 2 μm.

Appendix Tables

Appendix Table S1. Plasmids and strains used in the current study.

**Strain/Plasmid**	**Description**	**References or sources**
pSeSD	A Sulfolobus-E. coli shuttle vector carrying an expression cassette controlled under a synthetic strong promoter ParaS-SD	(1)
pGE-Cran1-KO	The genome-editing plasmid for *cran1* knockout	This study
pGE-SiRe_0087-KO	The genome-editing plasmid for *sire_0087* knockout	This study
Sis/pGE-Cran1-insitu -Flag	E233S harboring the genome-editing plasmid for *cran1* add a C-terminal flag tag	This study
Sis/pGE-Cran1-insitu-His	E233S harboring the genome-editing plasmid for *cran1* add a C-terminal His tag	This study
Cran1-insitu-flag/pGE-Cran1-KD-4S	Cran1-insitu-flag strains harboring the genome-editing plasmid for *cran1* knockdown	This study
Sis/pSeSD	E233S harboring pSeSD plasmid	This study
Sis/pSeSD-Cran1-C-His	E233S harboring pSeSD-Cran1-C-His	This study
Sis/pSeSD-Cran1-no tag	E233S harboring pSeSD-Cran1-no tag	This study
Sis/pSeSD-SiRe_0087-no tag	E233S harboring pSeSD-SiRe_0087-no tag	This study
Sis/pSeSD-SiRe_0086-SiRe_0087-no tag	E233S harboring SiRe_0086 and SiRe_0087 co-expression plasmids	This study
Sis/pSeSD-Cran1-K36A-C-His	E233S harboring pSeSD-Cran1-K36A-C-His	This study
Sis/pSeSD-Cran1-T37A-C-His	E233S harboring pSeSD-Cran1-T37A-C-His	This study
Sis/pSeSD-Cran1-F156A-C-His	E233S harboring pSeSD-Cran1-F156A-C-His	This study
Sis/pSeSD-Cran1-K36A-C-His	E233S harboring pSeSD-Cran1-K36A-C-His	This study

Sis/pSeSD-Cran1-T37A-C-His	E233S harboring pSeSD-Cran1-T37A-C-His	This study
Sis/pSeSD-Cran1-F156A-C-His	E233S harboring pSeSD-Cran1-F156A-C-His	This study
Sis/pSeSD-Cran1-D157A-C-His	E233S harboring pSeSD-Cran1-D157A-C-His	This study
Sis/pSeSD-Cran1-Q158A-C-His	E233S harboring pSeSD-Cran1-Q158A-C-His	This study
Sis/pSeSD-Cran1-P246A-C-His	E233S harboring pSeSD-Cran1-P246A-C-His	This study
Sis/pSeSD-Cran1-E247A-C-His	E233S harboring pSeSD-Cran1-E247A-C-His	This study
Sis/pSeSD-Cran1-P248A-C-His	E233S harboring pSeSD-Cran1-P248A-C-His	This study
Sis/pSeSD-Cran1-E249A-C-His	E233S harboring pSeSD-Cran1-E249A-C-His	This study
Sis/pSeSD-Cran1-H280A-C-His	E233S harboring pSeSD-Cran1-H280A-C-His	This study
Sis/pSeSD-Cran1-S281A-C-His	E233S harboring pSeSD-Cran1-S281A-C-His	This study
Sis/pSeSD-Cran1-T37A/H280A/S281A-C-His	E233S harboring pSeSD-Cran1-T37A/H280A/S281A-C-His	This study
Sis/pSeSD-Cran1-E338A/L391A/D393A-C-His	E233S harboring pSeSD-Cran1-E338A/L391A/D393A-C-His	This study
Sis/pSeSD-Cran1-E341A/E409A/E411A-C-His	E233S harboring pSeSD-Cran1-E341A/E409A/E411A-C-His	This study
Sis/pSeSD-Cran1-L391A/D393A/E409A/E411A-C-His	E233S harboring pSeSD-Cran1-L391A/D393A/E409A/E411A-C-His	This study

Sis/pSeSD-Saci_OLD	E233S harboring pSeSD-Saci_OLD strain	This study
cran1-Flag/pGE	Cran1 genome in situ Flag-tagged strain carrying pGE empty vector	This study
cran1-Flag/ pGE-KD-4spacers	Cran1 genome in situ Flag-tagged strain carrying pGE-Cran1 knockdown vector	This study
E. coli /pET22b-Cran1-C-His	Expression of WT-Cran1 having His-tag at the C-terminal	This study
E. coli /pET22b-Cran1-K36A-C-His	Expression of Cran1-K36A having His-tag at the C-terminal	This study
E. coli /pET22b-Cran1-T37A-C-His	Expression of Cran1-T37A having His-tag at the C-terminal	This study
E. coli /pET22b-Cran1-F156A-C-His	Expression of Cran1-F156A having His-tag at the C-terminal	This study
E. coli /pET22b-Cran1-D157A-C-His	Expression of Cran1-D157A having His-tag at the C-terminal	This study
E. coli /pET22b-Cran1-Q158A-C-His	Expression of Cran1-Q158A having His-tag at the C-terminal	This study
E. coli /pET22b-Cran1-P248A-C-His	Expression of Cran1-P248A having His-tag at the C-terminal	This study
E. coli /pET22b-Cran1-E249A-C-His	Expression of Cran1-E249A having His-tag at the C-terminal	This study
E. coli /pET22b-Cran1-H280A-C-His	Expression of Cran1-H280A having His-tag at the C-terminal	This study
E. coli /pET22b-Cran1-S281A-C-His	Expression of Cran1-S281A having His-tag at the C-terminal	This study
E. coli /pET22b-Cran1-T37A/H280A/S281A-C-His	Expression of Cran1-T37A/H280A/S281A having His-tag at the C-terminal	This study
E. coli /pET22b-Cran1-	Expression of Cran1-E338A/L391A/D393A	This study

E338A/L391A/D393A-C-His	having His-tag at the C-terminal	
E. coli /pET22b-Cran1-E341A/E409A/E411A-C-His	Expression of -Cran1-E341A/E409A/E411A having His-tag at the C-terminal	This study
E. coli /pET22b--Cran1-L391A/D393A/E409A/E411A-C-His	Expression of -Cran1-L391A/D393A/E409A/E411A having His-tag at the C-terminal	This study
MG1655/PET22b	*MG1655* strain carrying the PET22b empty vector	This study
MG1655/PET22b-SiRe_0086	*MG1655* strain carrying the PET22b-SiRe_0086 vector	This study
MG1655/PET22b-SiRe_0086-0087	*MG1655* strain carrying the PET22b-SiRe_0086-0087 vector	This study

Appendix Table S2. Oligonucleotides used as primers in the current study.

Primer	Sequence [a, b] (5′-3′)
Cran1-KO-spacer-F	AAGACTCTTCAAGCTAAAATCATGAAATTAATTAAGAACTGGA
Cran1-KO-spacer-R	AGCTCCAGTTCTTAATTAATTTCATGATTTTAGCTTGAAGAGT
Cran1-L-arm-F-sphI	AAGTACAATTGTGCTGCATGCGACAAAGACGGAAAGGCT
Cran1-L-arm-R	AACATTTATATTCCATCTTCTATTCCGGCTGACAAA
Cran1-R-arm-F	TTTGTCAGCCGGAATAGA AGATGGATTATAAATGTT
Cran1-R-arm-R-XhoI	TTAACATATTGGATGCTCGAGTTTCATTTACCATCTCTA
Cran1Flanking-F	GCTTACGTTACCGGTAGTCCAAAAC
Cran1Flanking-R	CTTCATAGCATACCAGTACGTTCG
Cran1-in situ-Sp-F	AAGAGCAACACTATAAATTATGGATCTTAGTATCTTATTCGAT
Cran1-in situ-Sp-R	AGCATCGAATAAGATACTAAGATCCATAATTTATAGTGTTGCT
Cran1insitu-L-arm-F	AAGTACAATTGTGCTGCATGCTTAGTAGGATTATAATACTG
Cran1insitu-L-arm-R	CTTATCGTCGTCATCCTTGTAATCTAGTGTTGCTTGTAGGGATT
Cran1insitu-R-arm-F	GATTACAAGGATGACGACGATAAGTAAATTATGGATCTTAGTAT
Cran1insitu-R-arm-R	TTAACATATTGGATGCTCGAGAAGAAGTAGTTGAGATGATT
Cran1insitu-L-His-R	ATGGTGATGGTGATGATGATGGTGATGGTGATGATGTAGTGTTGCTTGTAGGGATT
Cran1insitu-R-His-F	CATCATCACCATCACCATCATCATCACCATCACCATTAAATTATGGATCTTAGTAT
Flag-F	GATTACAAGGATGACGACGATAAG
His-F	ATGGTGATGGTGATGATG
Cran1-NdeI-F	GAATGAGGTGAAGCTCATATGAGGATTACTGAATTTTAC
Cran1-SalI-R	GGCCGCTTGATCAGCGTCGACTAGTGTTGCTTGGAGGG
Cran1-ST-salI-R	GGCCGCTTGATCAGCGTCGACTTATAGTGTTGCTTGGAGGGATT
Cran1K36A-F	AACGGATATGGA**GCA**ACTAATCTACTT
Cran1K36A-R	AAGTAGATTAGT**TGC**TCCATATCCGTT
Cran1T37A-F	AACGGATATGGAAAA**GCT**AATCTACTT

Cran1T37A-R	AAGTAGATT**AGC**TTTTCCATATCCGTT
Cran1F156A-F	TGTACCTATC**GCC**GACCAAGCGTATTTTG
Cran1F156A-R	CAAAATACGCTTGGTC**GGC**GATAGGTACA
Cran1D157A-F	TGTACCTATCTTC**GCC**CAAGCGTATTTTG
Cran1D157A-R	CAAAATACGCTTG**GGC**GAAGATAGGTACA
Cran1Q158A-F	TGTACCTATCTTCGAC**GCA**GCGTATTTTG
Cran1Q158A-R	CAAAATACGC**TGC**GTCGAAGATAGGTACA
Cran1P246A-F	AAGATATTACTAATT**GCA**GAACCAGAGGTGAA
Cran1P246A-R	TTCACCTCTGGTTC**TGC**AATTAGTAATATCTT
Cran1E247A-F	AAGATATTACTAATTGAA**GCA**CCAGAGGTGAA
Cran1E247A-R	TTCACCTCTGG**TGC**TTCAATTAGTAATATCTT
Cran1P248A-F	AATTGAAGAA**GCA**GAGGTGAATATGC
Cran1P248A-R	GCATATTCACCTC**TGC**TTCTTCAATT
Cran1E249A-F	AATTGAAGAACCA**GCG**GTGAATATGC
Cran1E249A-R	GCATATTCAC**CGC**TGGTTCTTCAATT
Cran1H280A-F	CCTTACAACA**GCT**TCCCCATACATCG
Cran1H280A-R	CGATGTATGGGGA**AGC**TGTTGTAAGG
Cran1S281A-F	CCTTACAACACAT**GCC**CCATACATCG
Cran1S281A-R	CGATGTATGG**GGC**ATGTGTTGTAAGG
Cran1H280AS281A-F	CCTTACAACA**GCTGCC**CCATACATCG
Cran1H280AS281A-R	CGATGTATGG**GGCAGC**TGTTGTAAGG
Cran1-E338A-F	ATACTGACAAGT**GCG**TTAGCAGAA
Cran1-E338A-R	TTCTGCTAA**CGC**ACTTGTCAGTAT
Cran1-L391A-D393A-F	CGTGATATTCCTTGGA**GCA**TGT**GCC**AAGCTTGATATGACCTTAAAGGAT
Cran1-L391A-D393A-R	ATCCTTTAAGGTCATATCAAGCTT**GGC**ACA**TGC**TCCAAGGAATATCACG
Cran1-E341A-F	CAAGTGAGTTAGCA**GCA**CCCTCTGTAATAGTGAAT

Cran1-E341A-R	ATTCACTATTACAGAGGG**TGC**TGCTAACTCACTTG
Cran1-E409A-E411A-F	GCGTTCCTTTAAGTCGT**GCG**GTA**GCG**AATTTCTATAGTAAACAAG
Cran1-E409A-E411A-R	CTTGTTTACTATAGAAATT**CGC**TAC**CGC**ACGACTTAAAGGAACGC
Sac_0157-NdeI-F	GAATGAGGTGAAGCTCATATGAAGCTTATTGAGTTTTACGT
Sac_0157-SalI-R	GGCCGCTTGATCAGCGTCGACTATAGTTTCTTCGAAAGATT
Cran1KD-F1	GCTAATCTACTATAGAATTGAAAGTAGATTAGTTTTTCCATATCCGTTATAGCCAACGA
Cran1KD-F2	CTACGGCTAATCTACTATAGAATTGAAAGTAGATTAGTTTTTCCATATCCGTTATAGCC
Cran1KD-R1	CTAATCTACTTTCAATTCTATAGTAGATTAGCCGTAGTCGTTGGCTATAACGGATATGG
Cran1KD-R2	CTTTCAATTCTATAGTAGATTAGCCGTAGTCGTTGGCTATAACGGATATGGAAAAA
0087-SalI-ST-R	GGCCGCTTGATCAGCGTCGACCTACAAGATGTAAAATATCA
0087-NdeI-F	GAATGAGGTGAAGCTCATATGATGGATCTTAGTATCTTATT
FAM-P$_{Cran1}$-F	AGATAATACTCCGTTTTTCTCTAACAATAGATTAACGTTAAAGAAGCCTGCTAACGATTATATTTTATTCTGCTAAGTATTACCGTAGAGTGATCTGTA
P$_{Cran1}$-R	TACAGATCACTCTACGGTAATACTTAGCAGAATAAAATATAATCGTTAGCAGGCTTCTTTAACGTTAATCTATTGTTAGAGAAAAACGGAGTATTATCT
0087KOspacer-F	AAGTCTGATAGGGATCAAAAAACACATGTTGGTGGGGTAATTT
0087KOspacer-R	AGCAAATTACCCCACCAACATGTGTTTTTTGATCCCTATCAGA
0087KOL-arm-sphI-F	AAGTACAATTGTGCTGCATGCGGATTATAATACTGACAAGT

0087KOL-arm-R	AATTTTTTATCATAATCACGAATTTATAGTGTTGCTTGGA
0087KOR-arm-F	TCCAAGCAACACTATAAATTCGTGATTATGATAAAAAATT
0087KOR-arm-xhoI-R	TTAACATATTGGATGCTCGAGCGGCTTCATTATTGGGAGAT
0087KO-Flanking-F	GTCTTTCCTTACAACACATT
0087KO-Flanking-R	GGTTATTTGCAAATAAGTTA

[a] The underlined denote sites of restriction enzymes. [b] The mutated codons are indicated in boldface.

1. Peng, N., Deng, L., Mei, Y.X., Jiang, D.Q., Hu, Y.M., Awayez, M., Liang, Y.X. and She, Q.X. (2012) A Synthetic Arabinose-Inducible Promoter Confers High Levels of Recombinant Protein Expression in Hyperthermophilic Archaeon. *Appl Environ Microb*, **78**, 5630-5637.
