# Supplementary figures and images for: Cran1, member of a new class of OLD family ATPases, functions in cell cycle progression in an archaeon
Source: EMBO Rep. 2025 Dec 2;27(1):208–29. doi: 10.1038/s44319-025-00650-y (PMC12796447; doi:10.1038/s44319-025-00650-y)

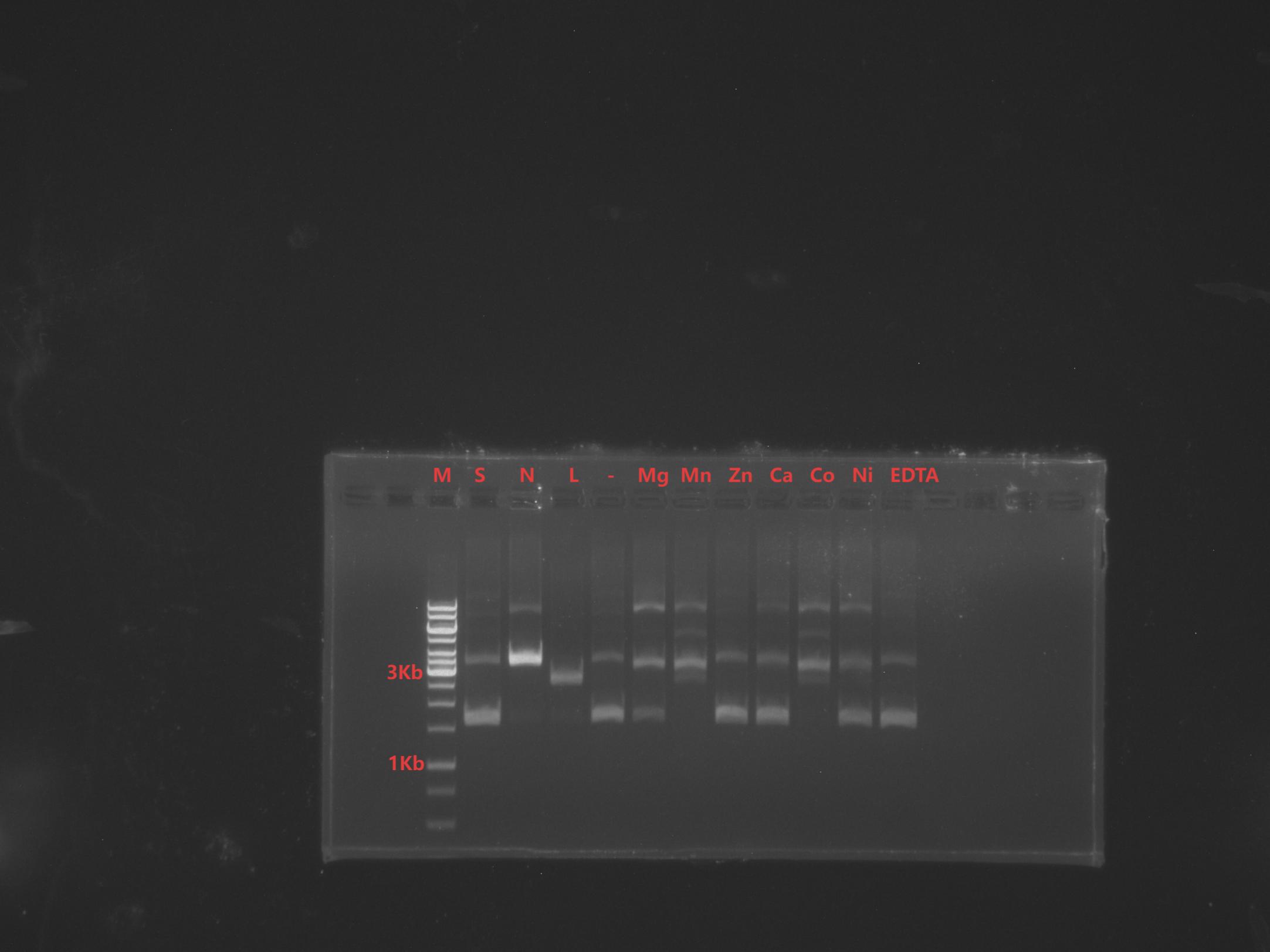

Supplement: Supplementary file 5 — Source data Fig. 2 [file 44319_2025_650_MOESM5_ESM.zip › Figure 2D.jpg]

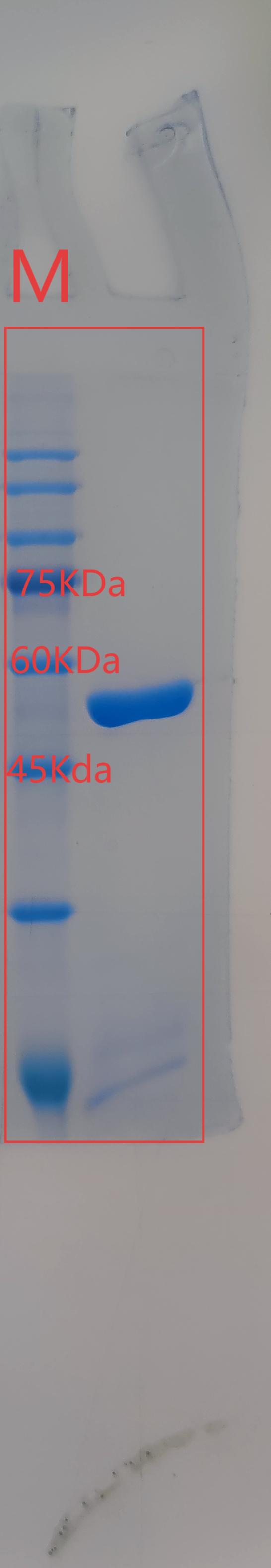

Supplement: Supplementary file 5 — Source data Fig. 2 [file 44319_2025_650_MOESM5_ESM.zip › Figure 2C.jpg]

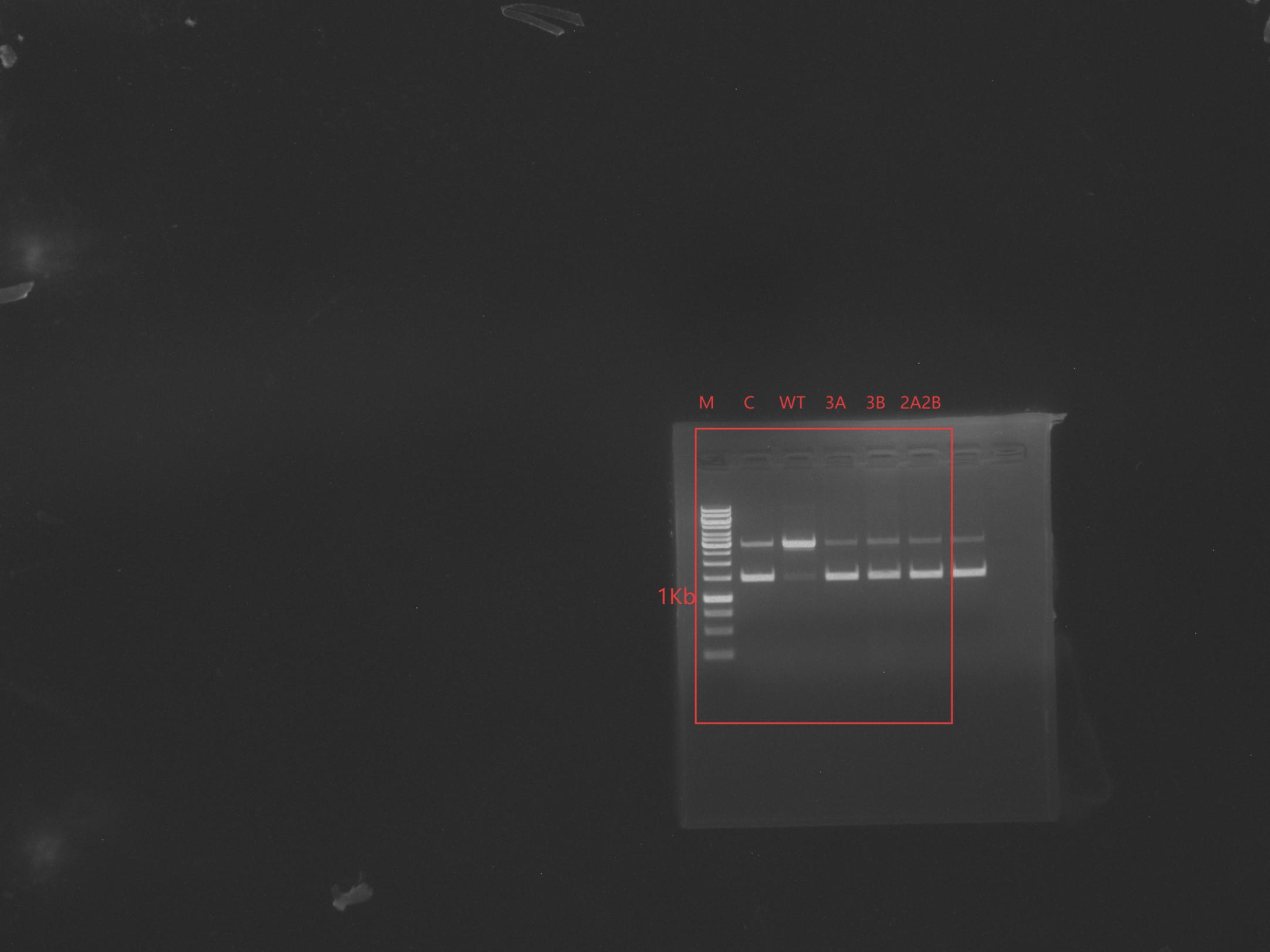

Supplement: Supplementary file 6 — Source data Fig. 3 [file 44319_2025_650_MOESM6_ESM.zip › Figure 3B.jpg]

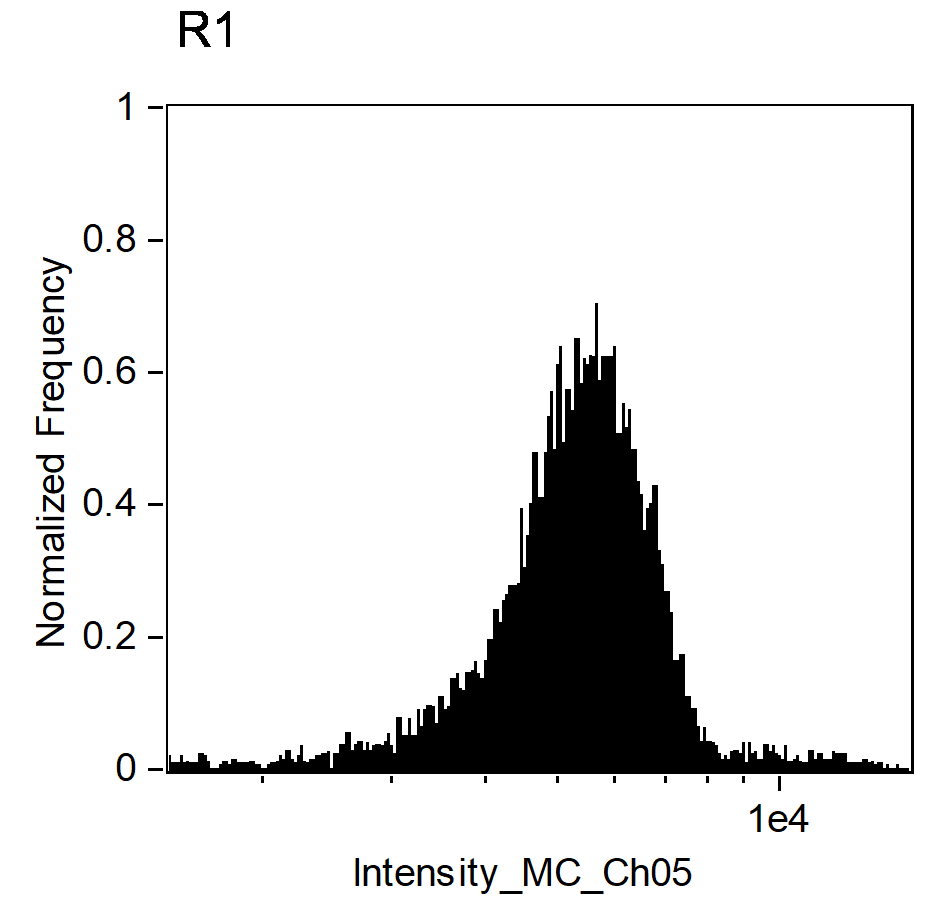

Supplement: Supplementary file 7 — Source data Fig. 4 [file 44319_2025_650_MOESM7_ESM.zip › Figure 4A 0h.png]

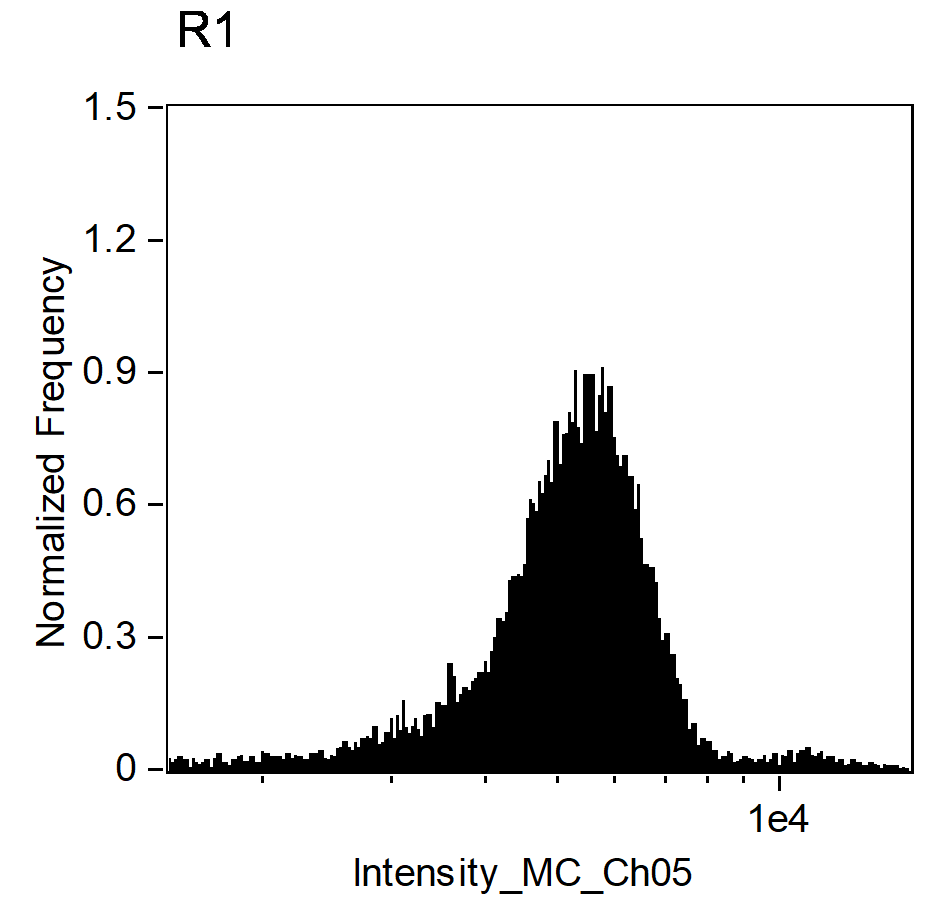

Supplement: Supplementary file 7 — Source data Fig. 4 [file 44319_2025_650_MOESM7_ESM.zip › Figure 4A 1h.png]

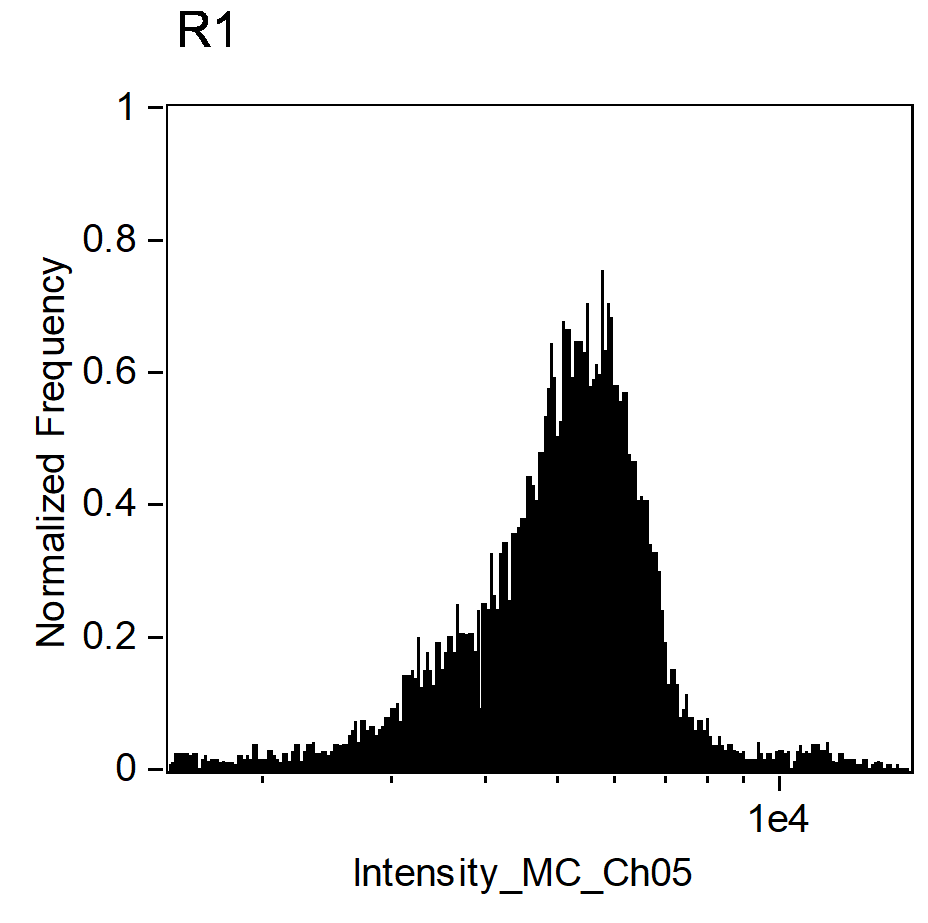

Supplement: Supplementary file 7 — Source data Fig. 4 [file 44319_2025_650_MOESM7_ESM.zip › Figure 4A 2h.png]

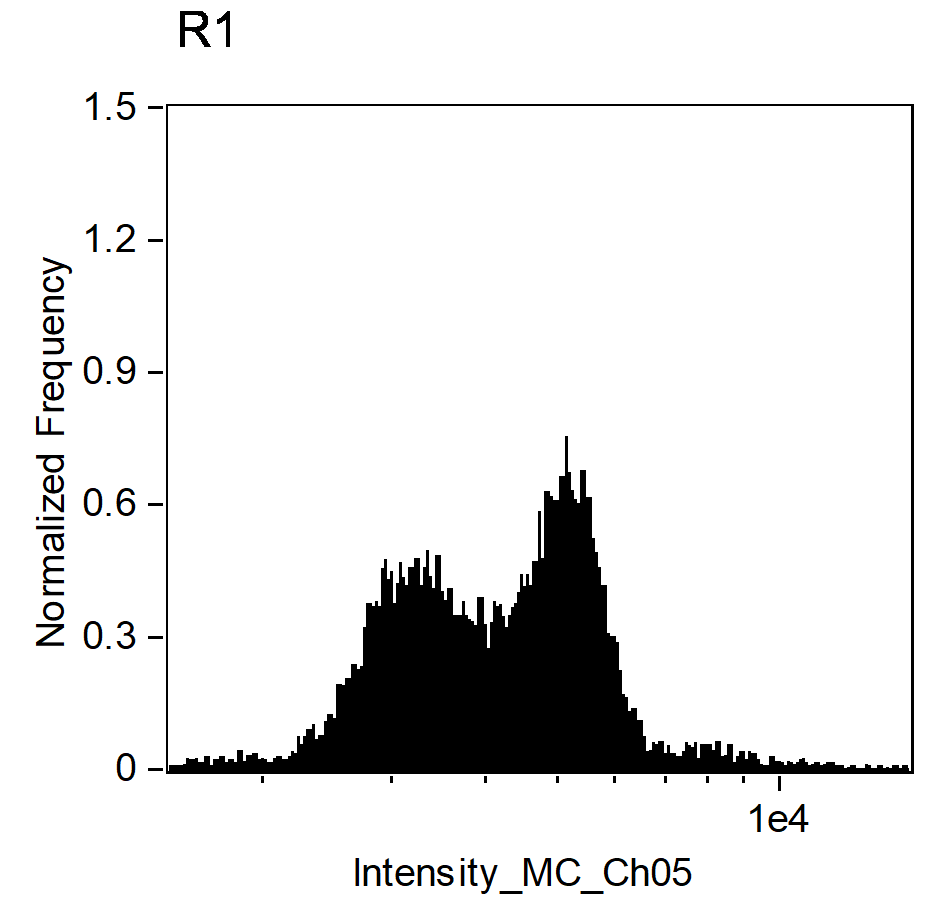

Supplement: Supplementary file 7 — Source data Fig. 4 [file 44319_2025_650_MOESM7_ESM.zip › Figure 4A 3h.png]

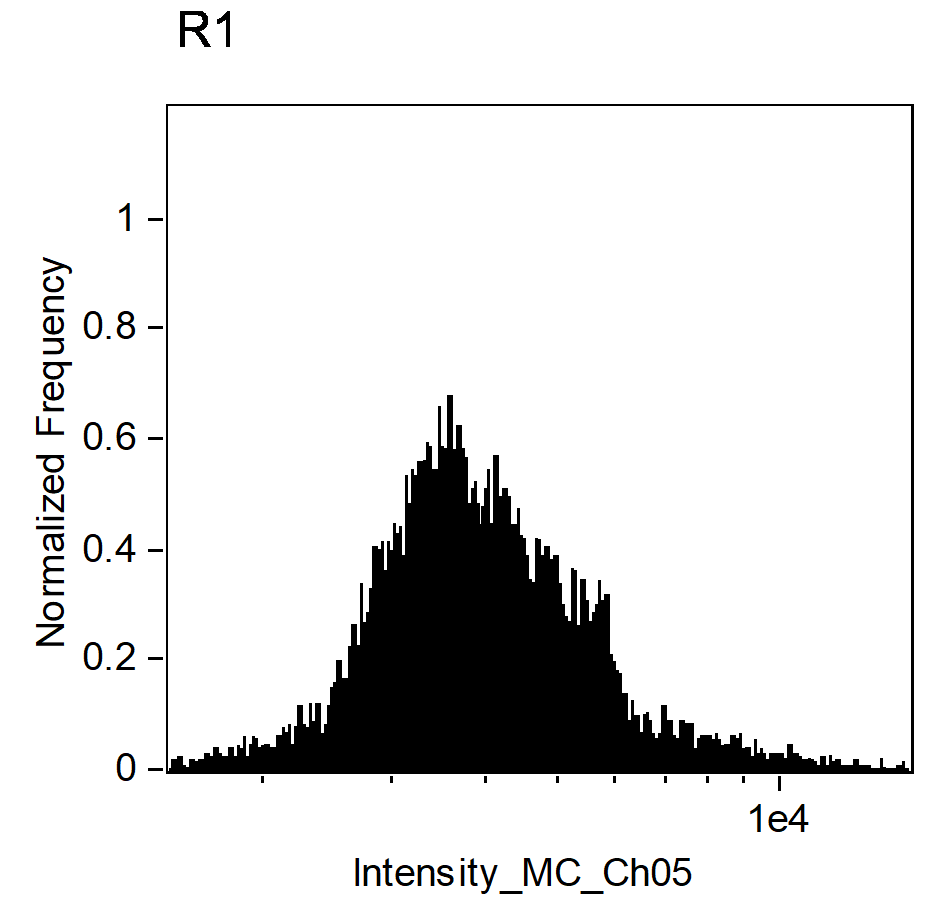

Supplement: Supplementary file 7 — Source data Fig. 4 [file 44319_2025_650_MOESM7_ESM.zip › Figure 4A 4h.png]

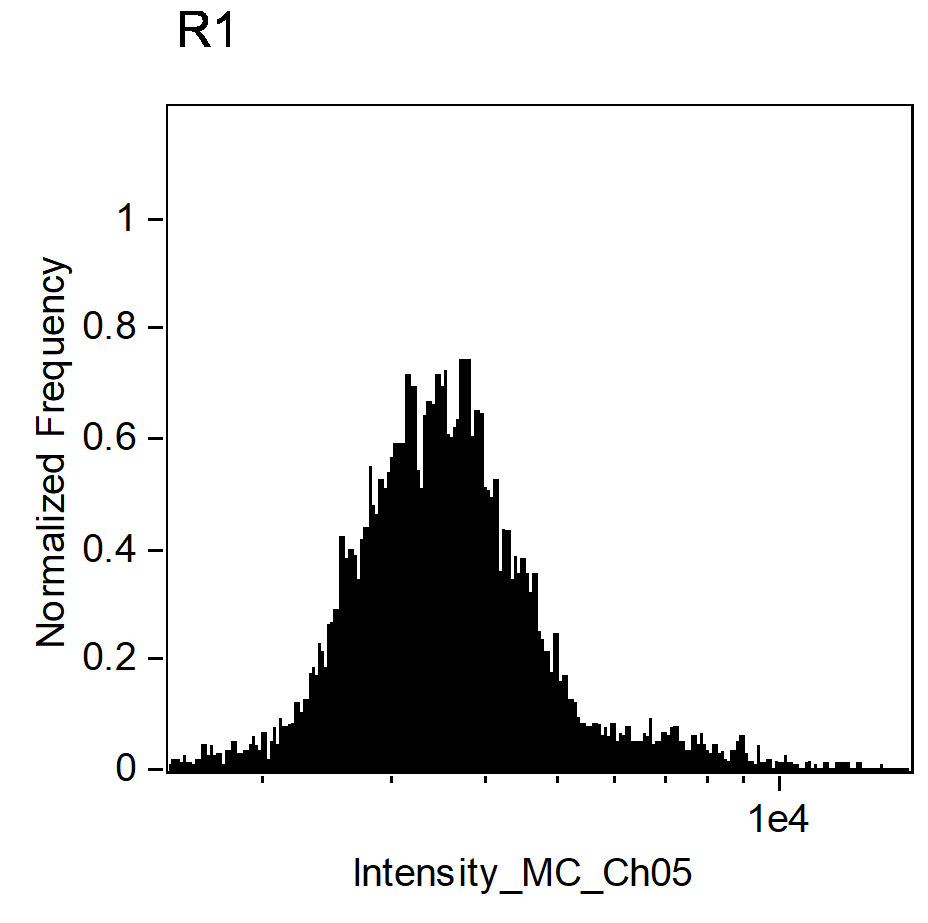

Supplement: Supplementary file 7 — Source data Fig. 4 [file 44319_2025_650_MOESM7_ESM.zip › Figure 4A 5h.png]

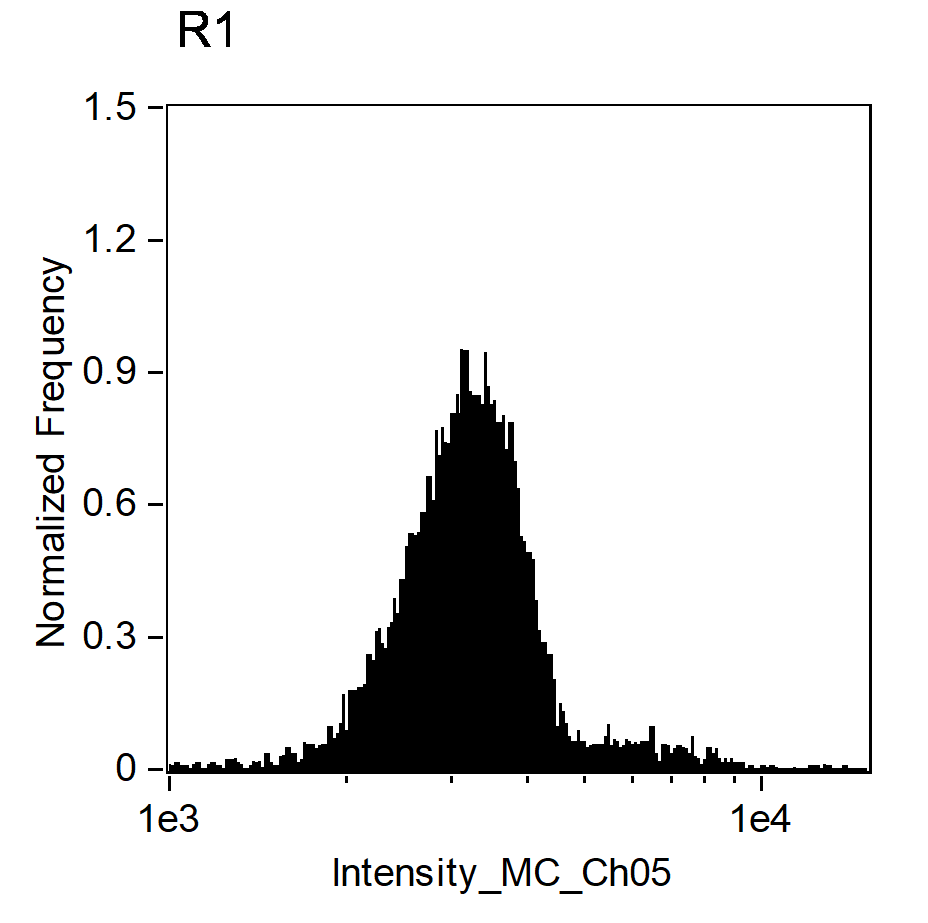

Supplement: Supplementary file 7 — Source data Fig. 4 [file 44319_2025_650_MOESM7_ESM.zip › Figure 4A 6h.png]

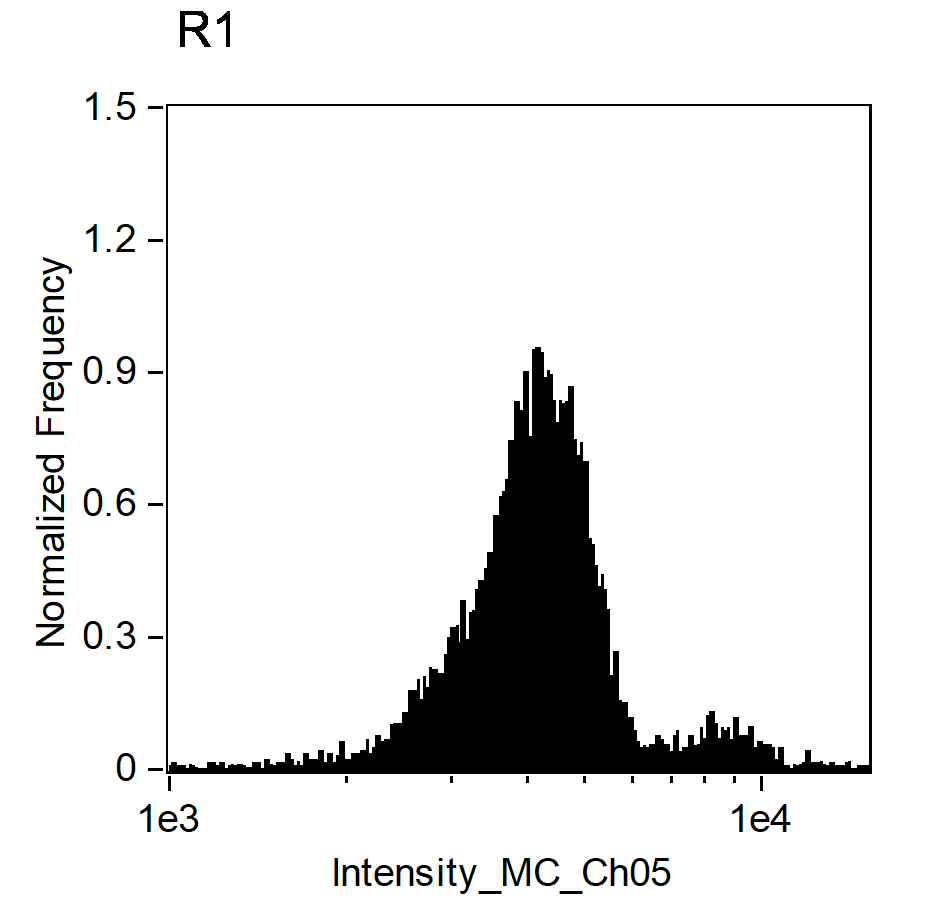

Supplement: Supplementary file 7 — Source data Fig. 4 [file 44319_2025_650_MOESM7_ESM.zip › Figure 4A 7h.png]

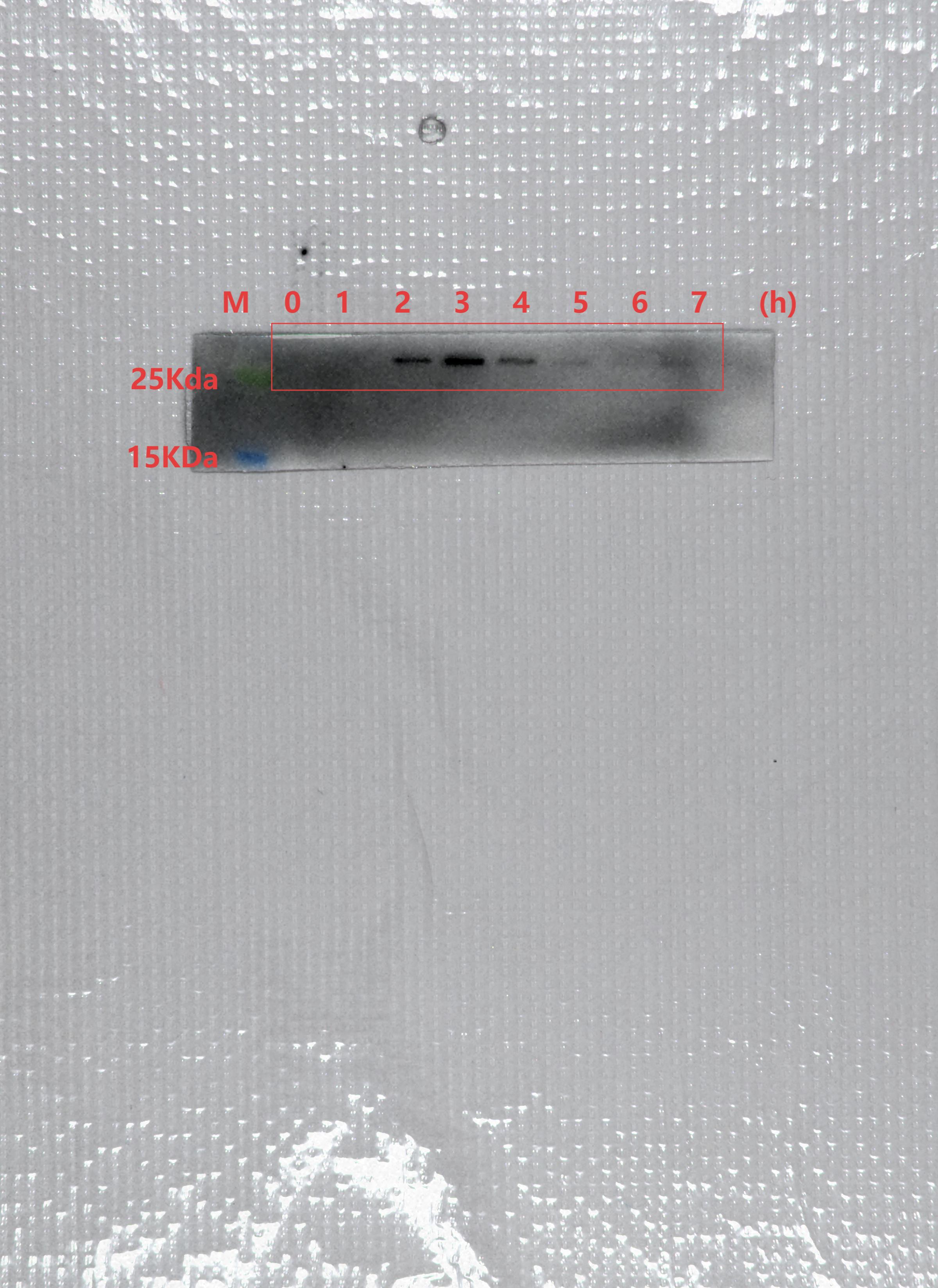

Supplement: Supplementary file 7 — Source data Fig. 4 [file 44319_2025_650_MOESM7_ESM.zip › Figure 4B cdvB.jpg]

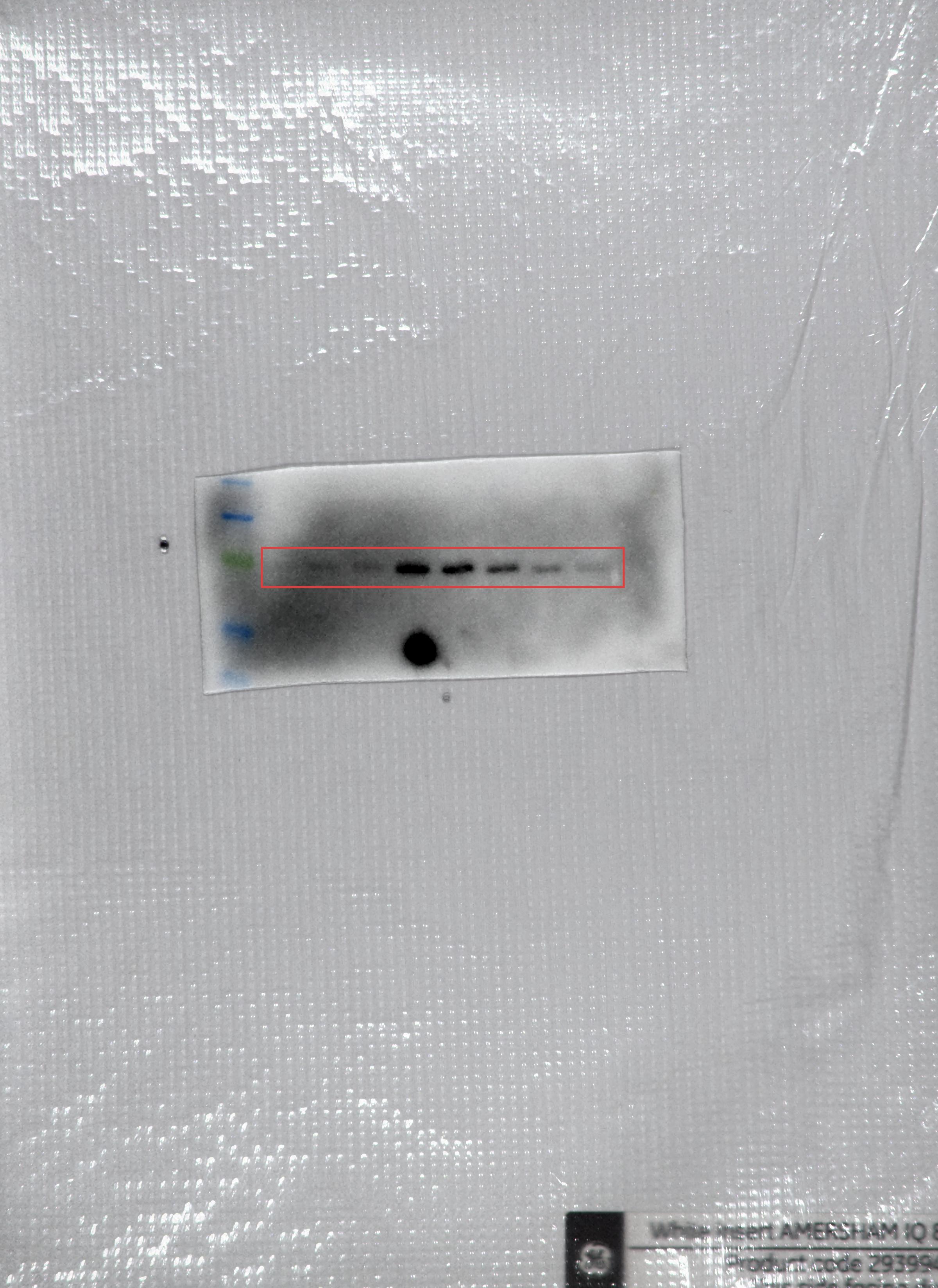

Supplement: Supplementary file 7 — Source data Fig. 4 [file 44319_2025_650_MOESM7_ESM.zip › Figure 4B cdvB1.jpg]

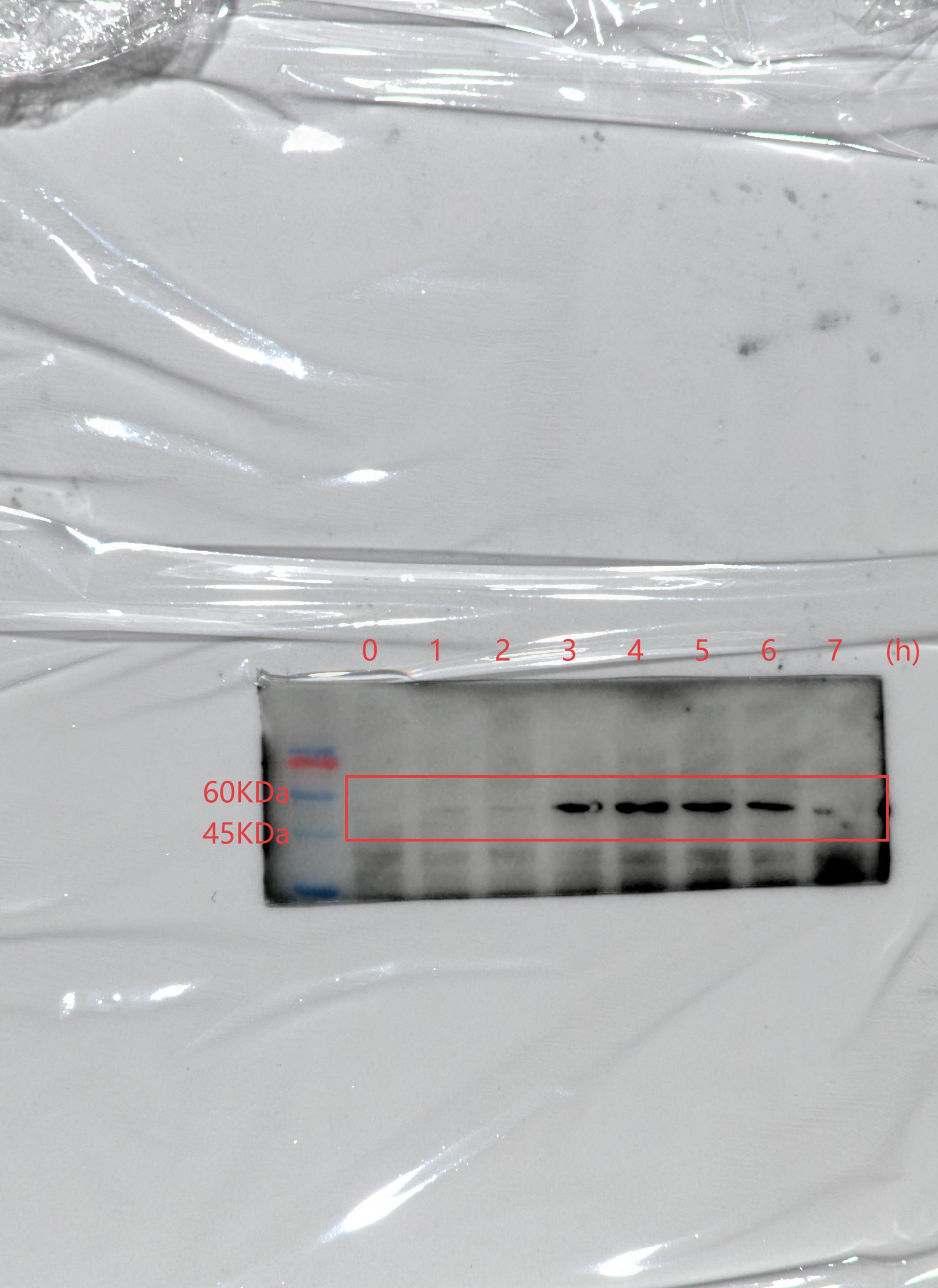

Supplement: Supplementary file 7 — Source data Fig. 4 [file 44319_2025_650_MOESM7_ESM.zip › Figure 4B flag.jpg]

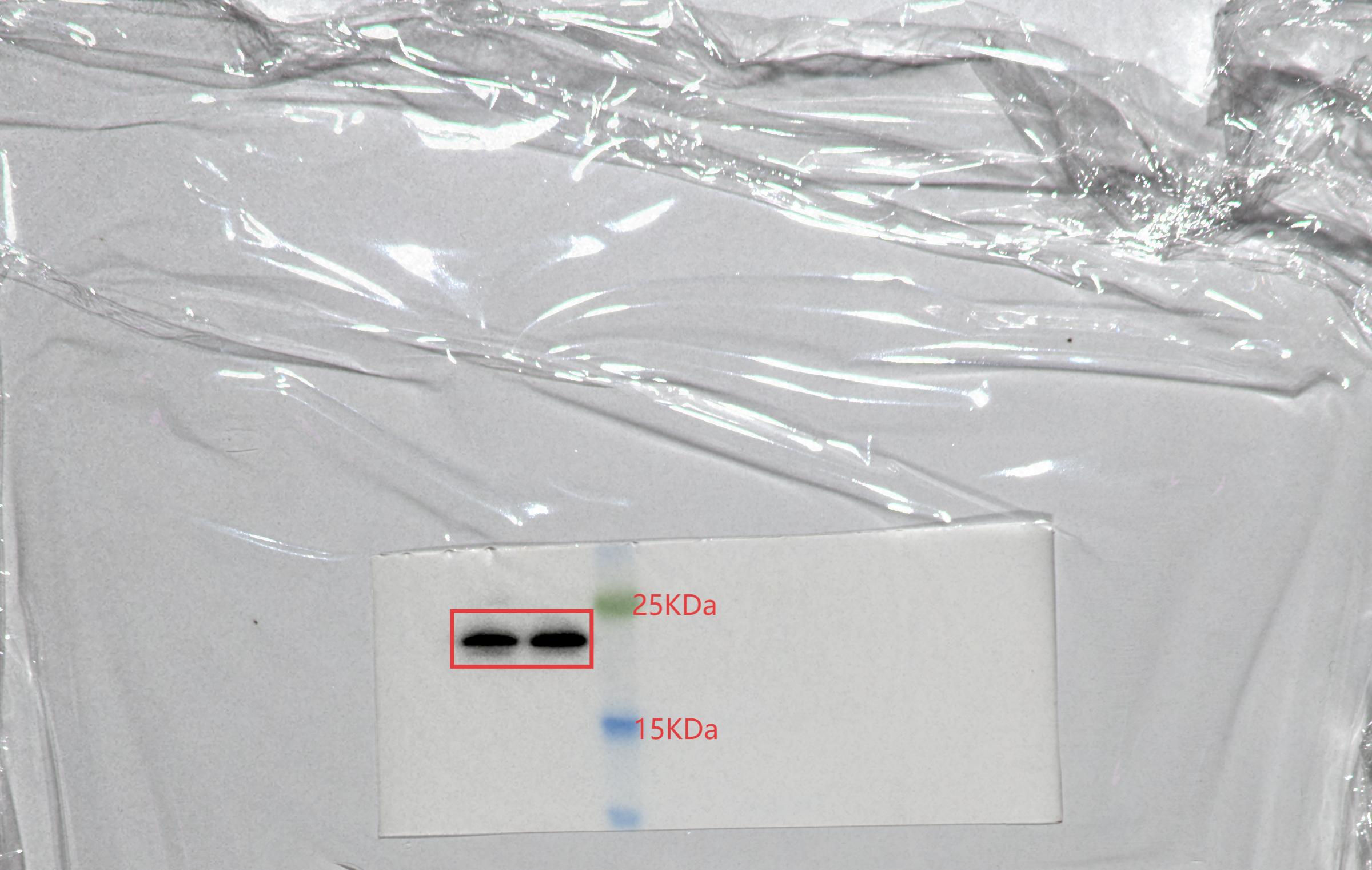

Supplement: Supplementary file 7 — Source data Fig. 4 [file 44319_2025_650_MOESM7_ESM.zip › Figure 4E TBP.jpg]

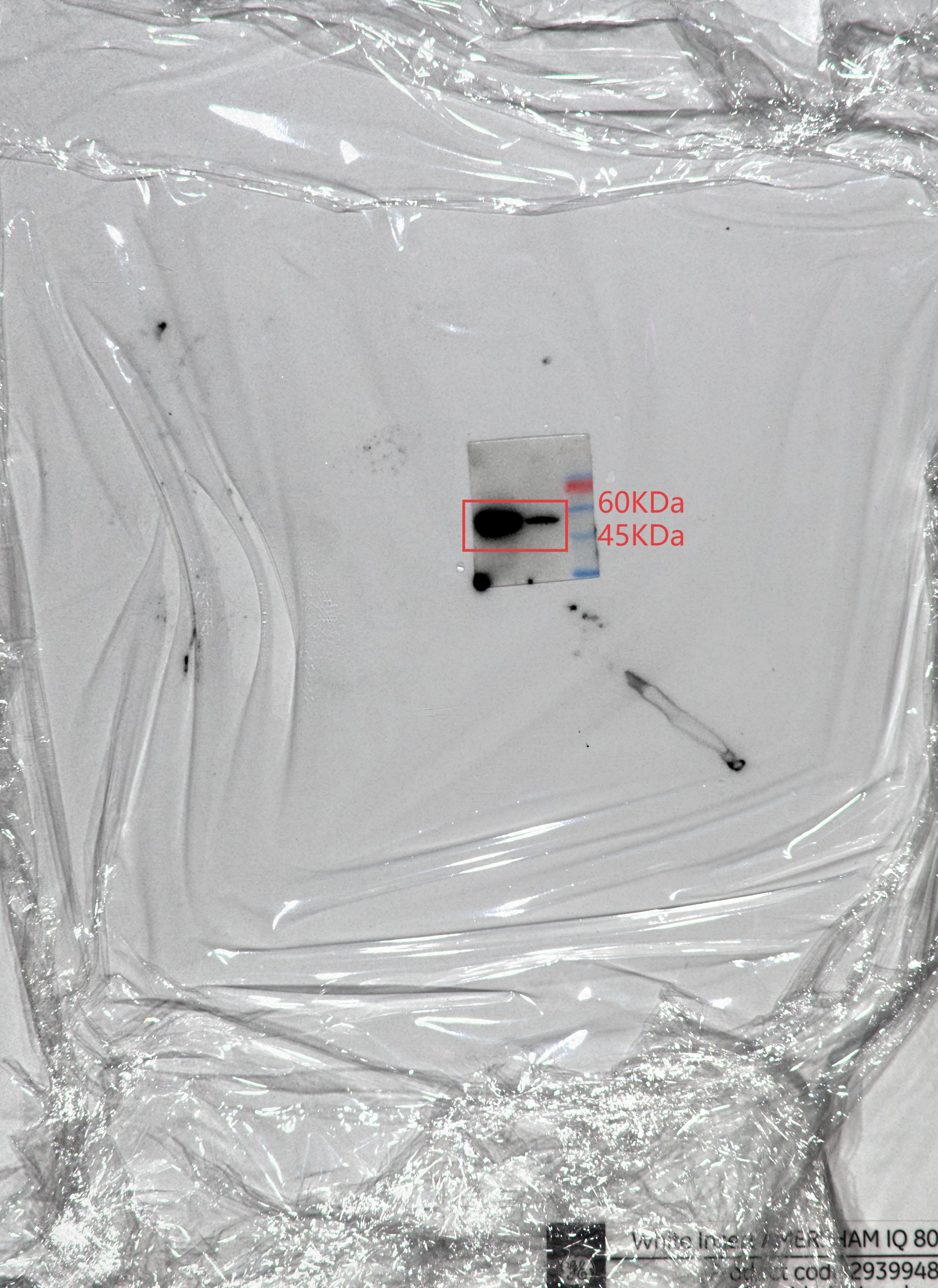

Supplement: Supplementary file 7 — Source data Fig. 4 [file 44319_2025_650_MOESM7_ESM.zip › Figure 4E falg.jpg]

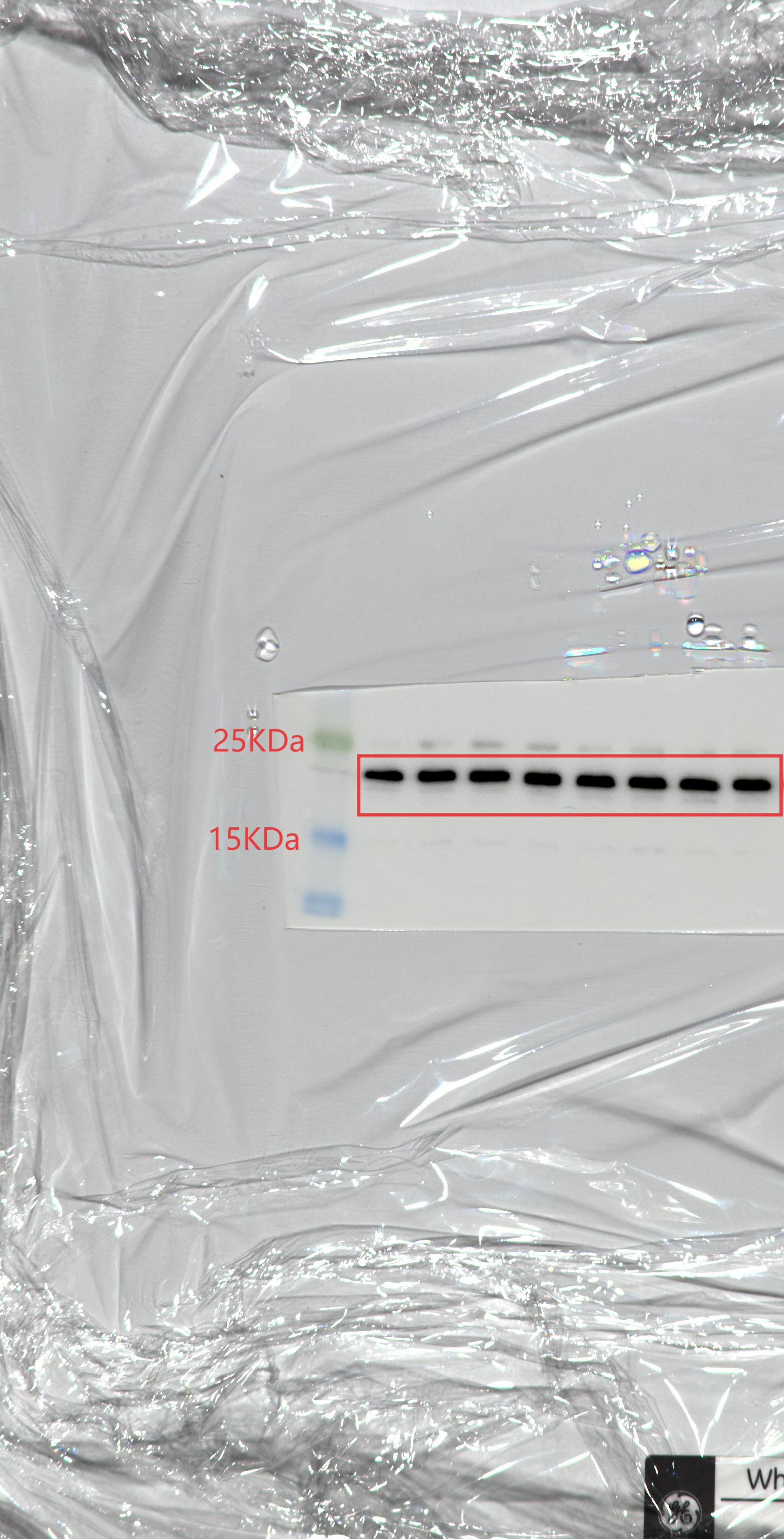

Supplement: Supplementary file 7 — Source data Fig. 4 [file 44319_2025_650_MOESM7_ESM.zip › Figure 4B TBP.jpg]

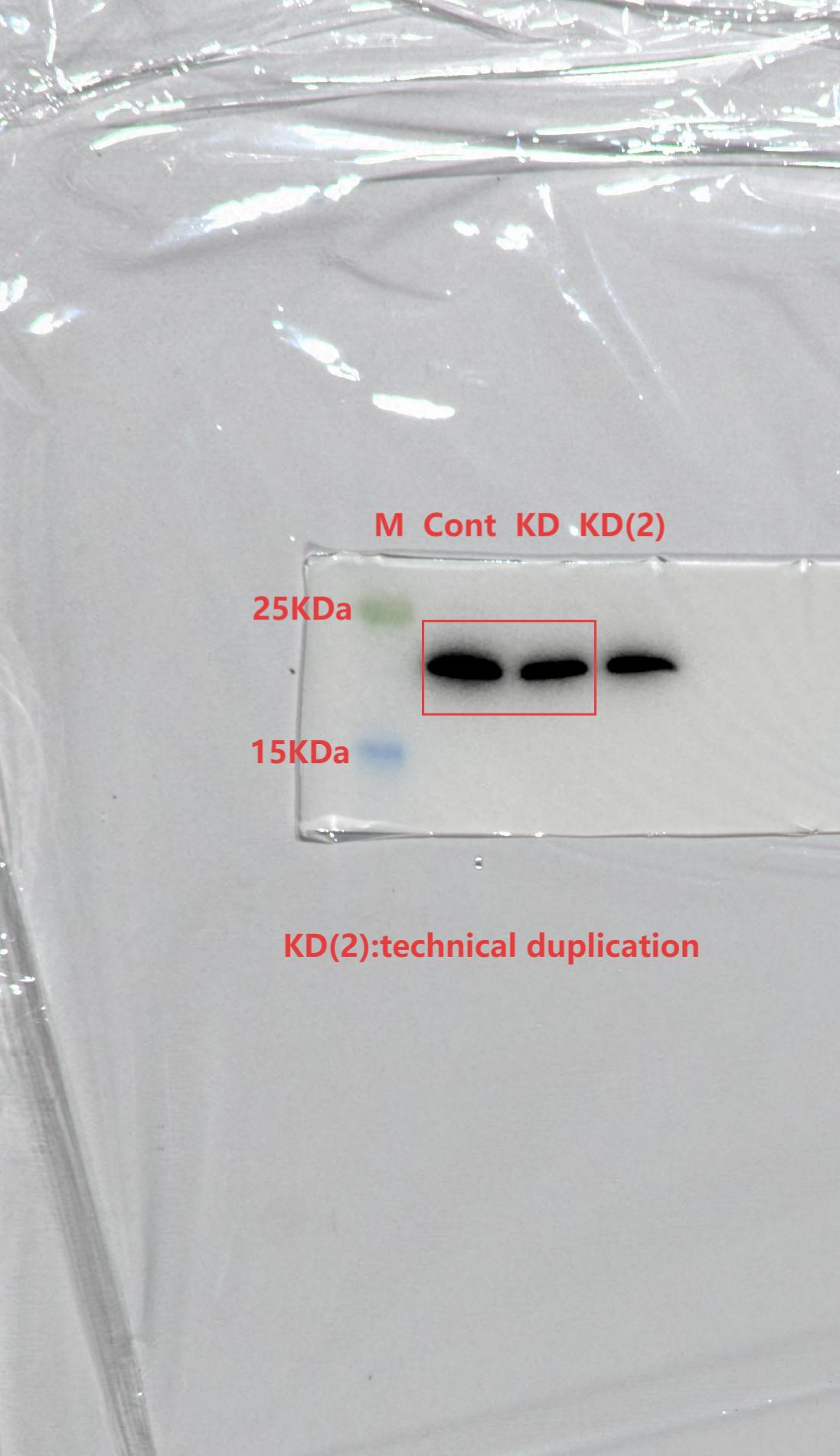

Supplement: Supplementary file 8 — Source data Fig. 5 [file 44319_2025_650_MOESM8_ESM.zip › Figure 5B TBP.jpg]

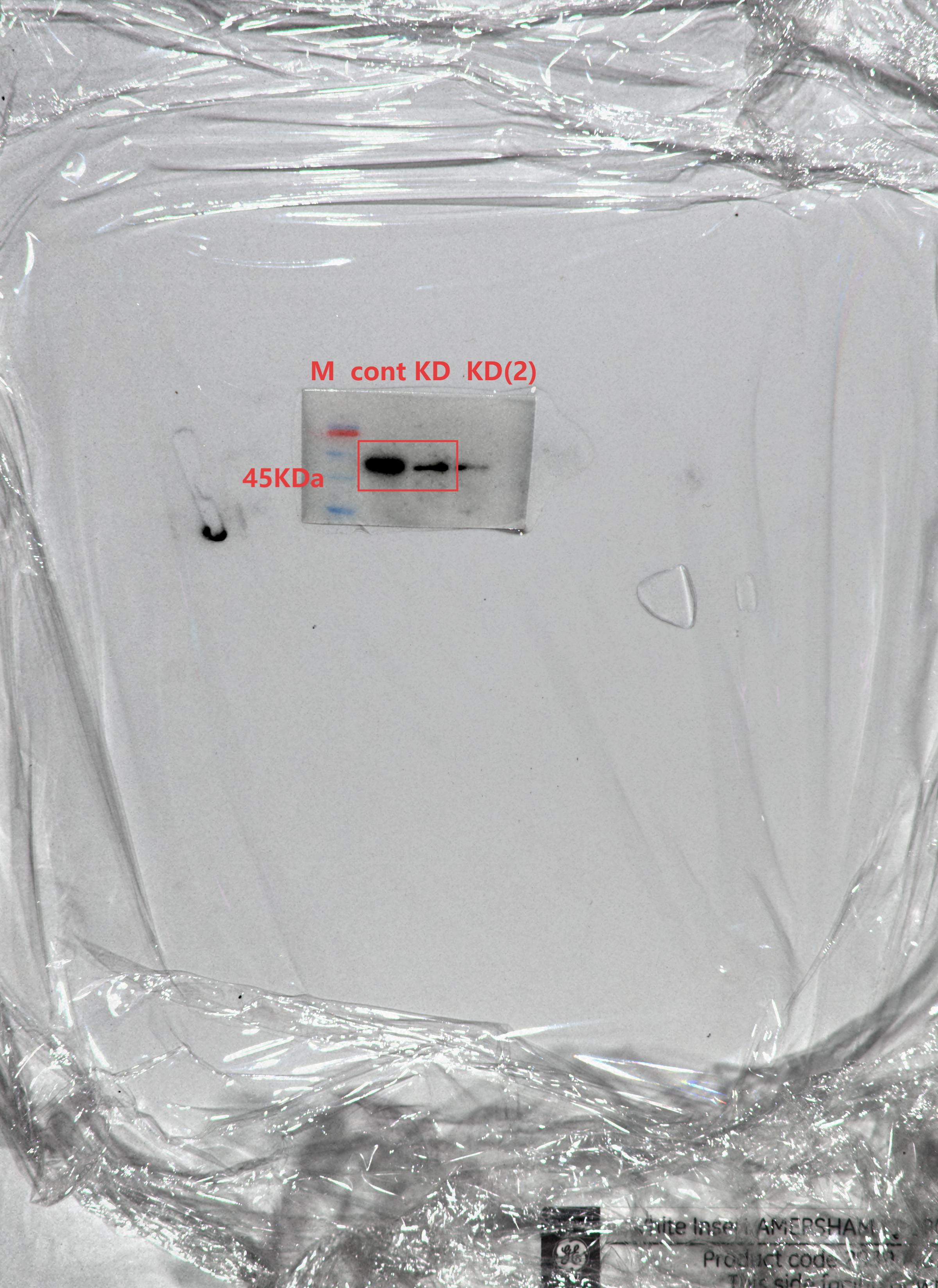

Supplement: Supplementary file 8 — Source data Fig. 5 [file 44319_2025_650_MOESM8_ESM.zip › Figure 5B flag.jpg]

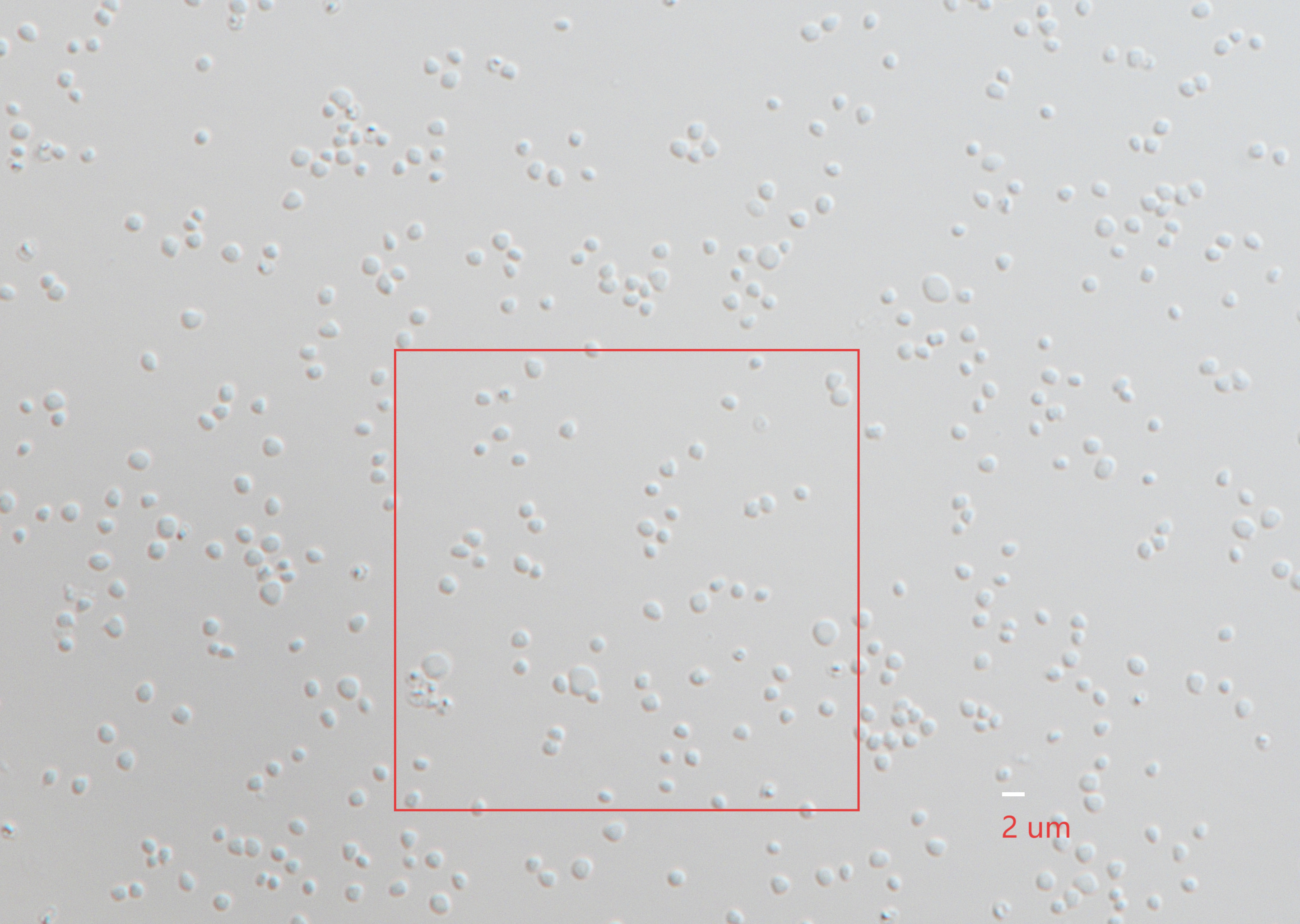

Supplement: Supplementary file 8 — Source data Fig. 5 [file 44319_2025_650_MOESM8_ESM.zip › Figure 5C SisOLD KD.png]

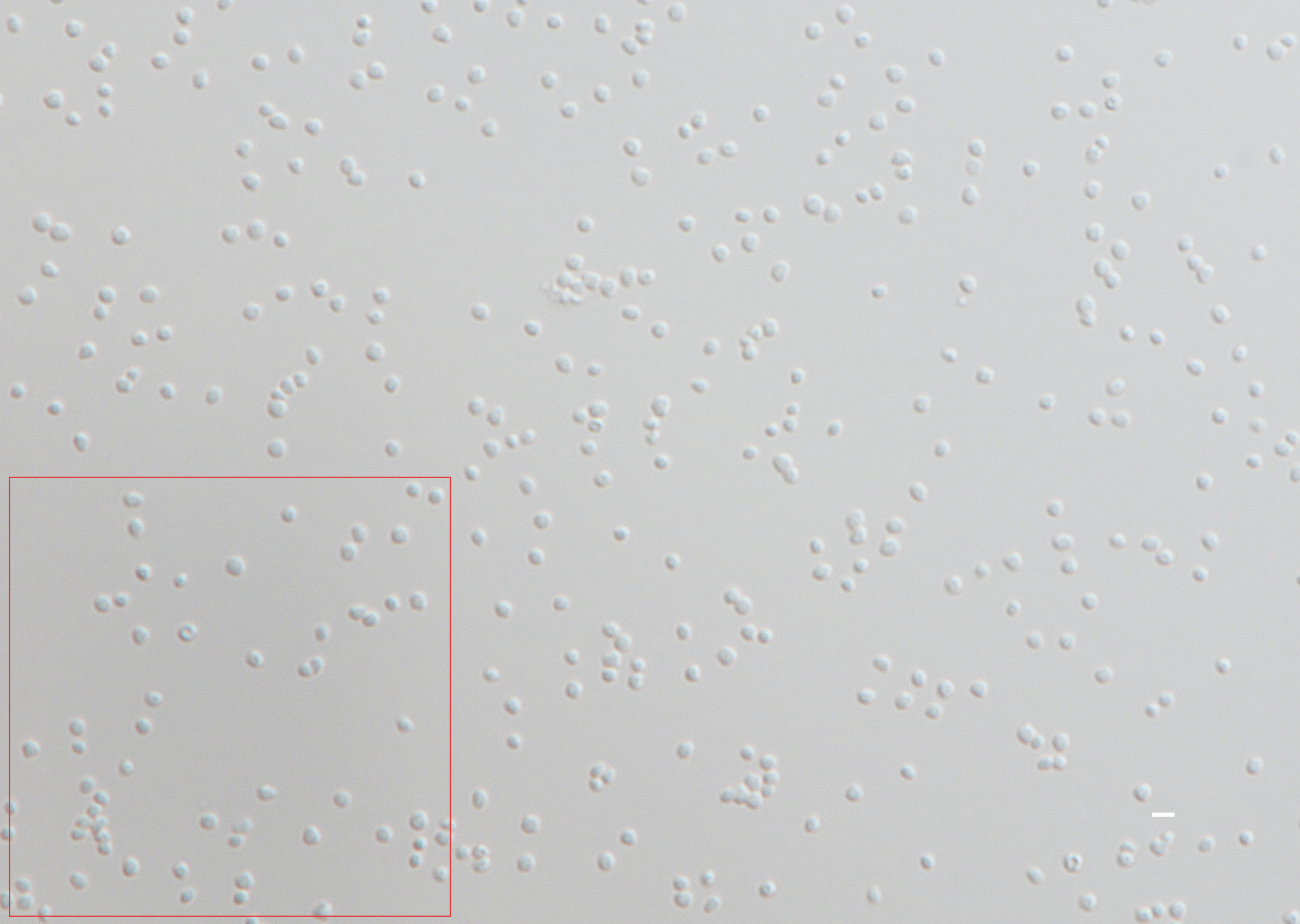

Supplement: Supplementary file 8 — Source data Fig. 5 [file 44319_2025_650_MOESM8_ESM.zip › Figure 5C cont.png]

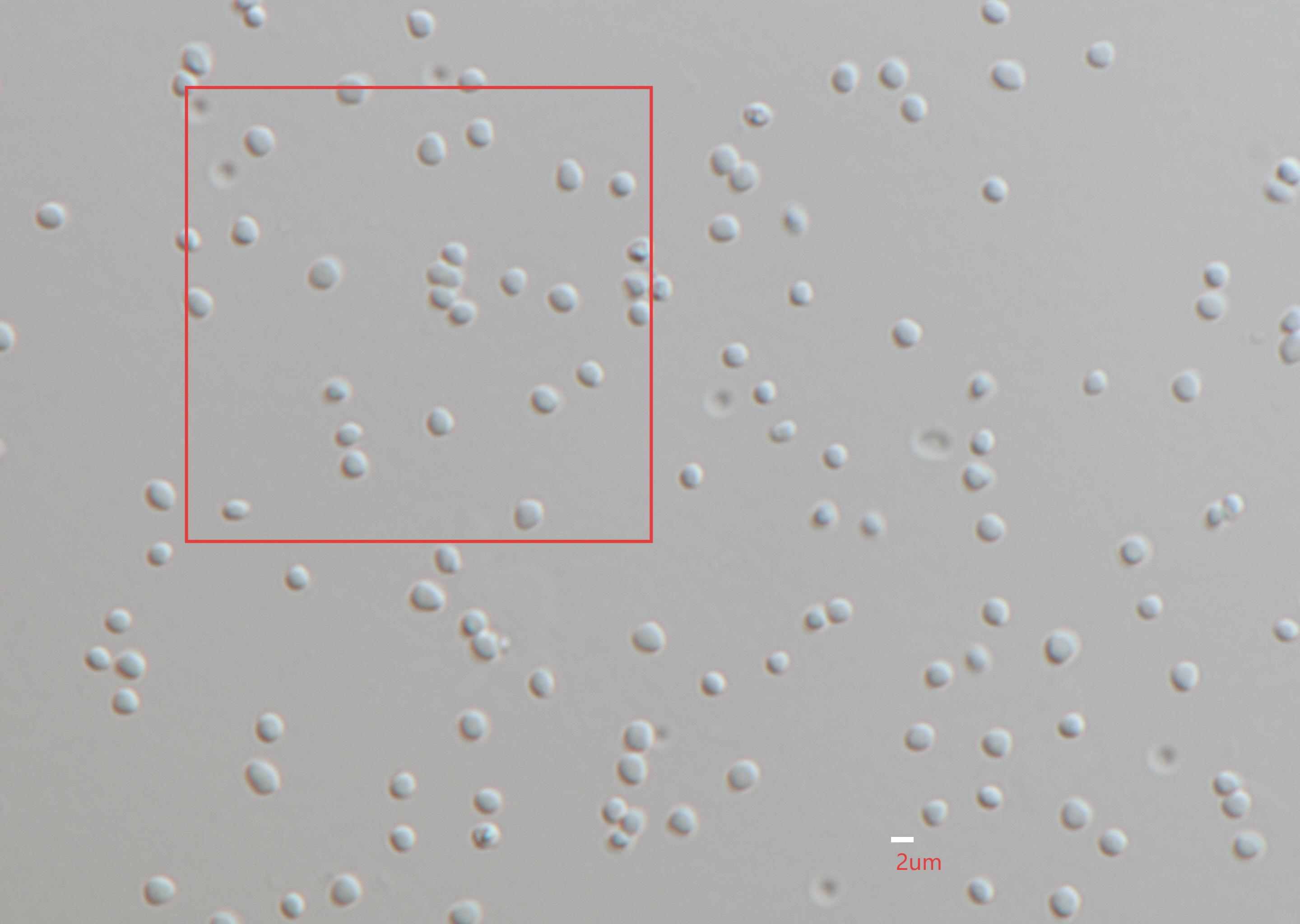

Supplement: Supplementary file 9 — Source data Fig. 6 [file 44319_2025_650_MOESM9_ESM.zip › Figure 6B 0h.jpg]

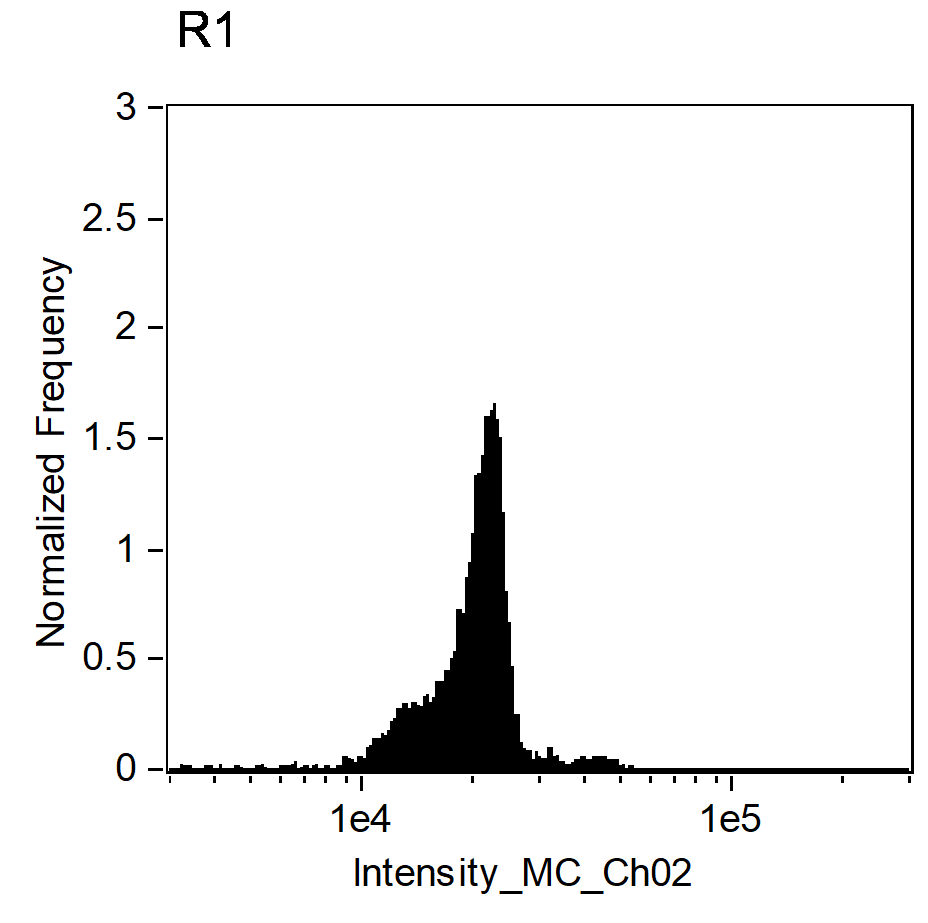

Supplement: Supplementary file 9 — Source data Fig. 6 [file 44319_2025_650_MOESM9_ESM.zip › Figure 6D FC 0086-0h.png]

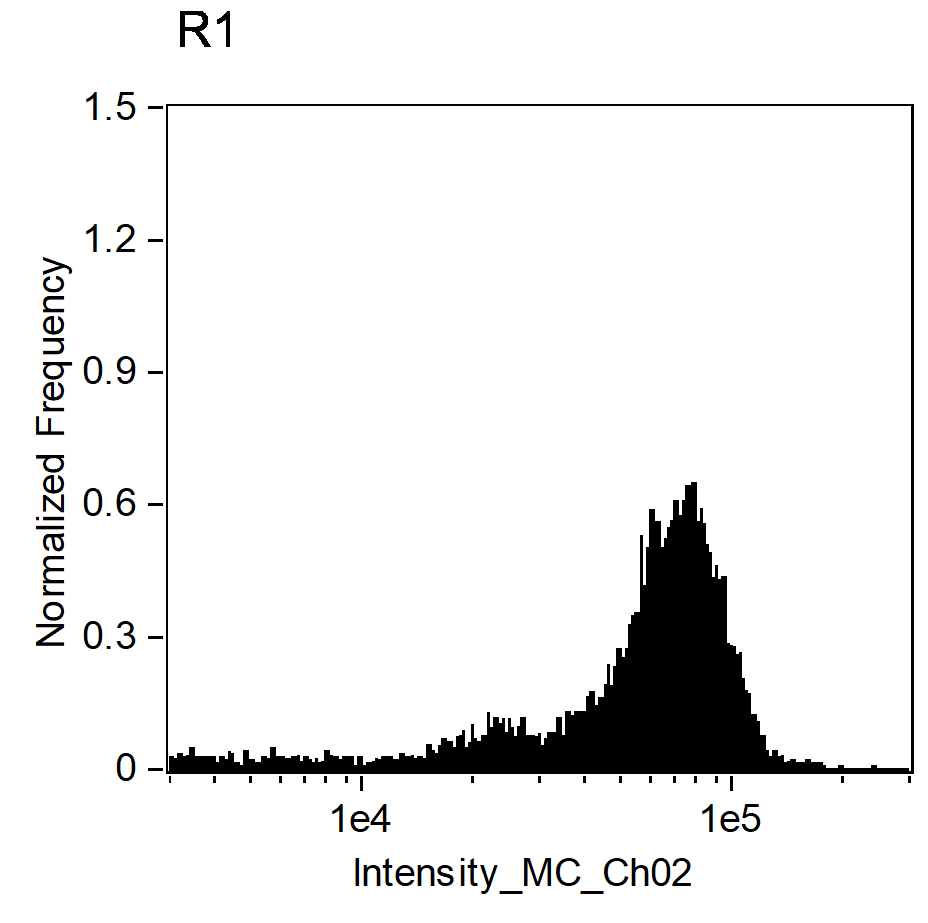

Supplement: Supplementary file 9 — Source data Fig. 6 [file 44319_2025_650_MOESM9_ESM.zip › Figure 6D FC 0086-12h.png]

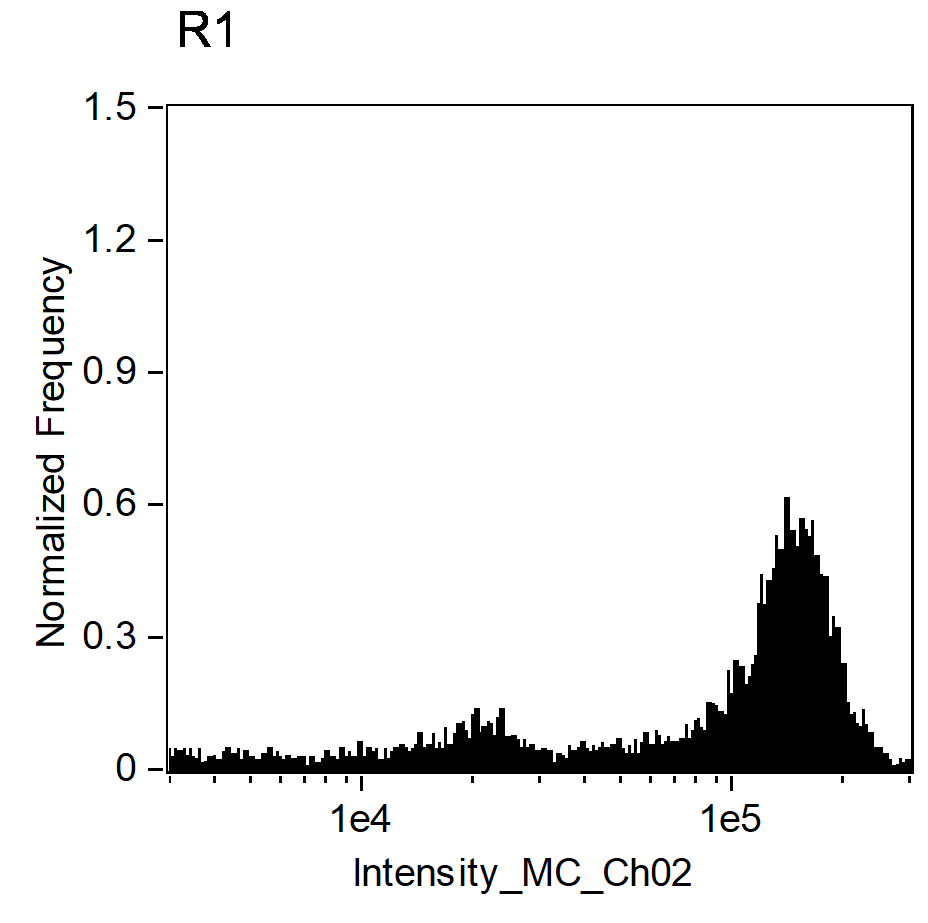

Supplement: Supplementary file 9 — Source data Fig. 6 [file 44319_2025_650_MOESM9_ESM.zip › Figure 6D FC 0086-24h.png]

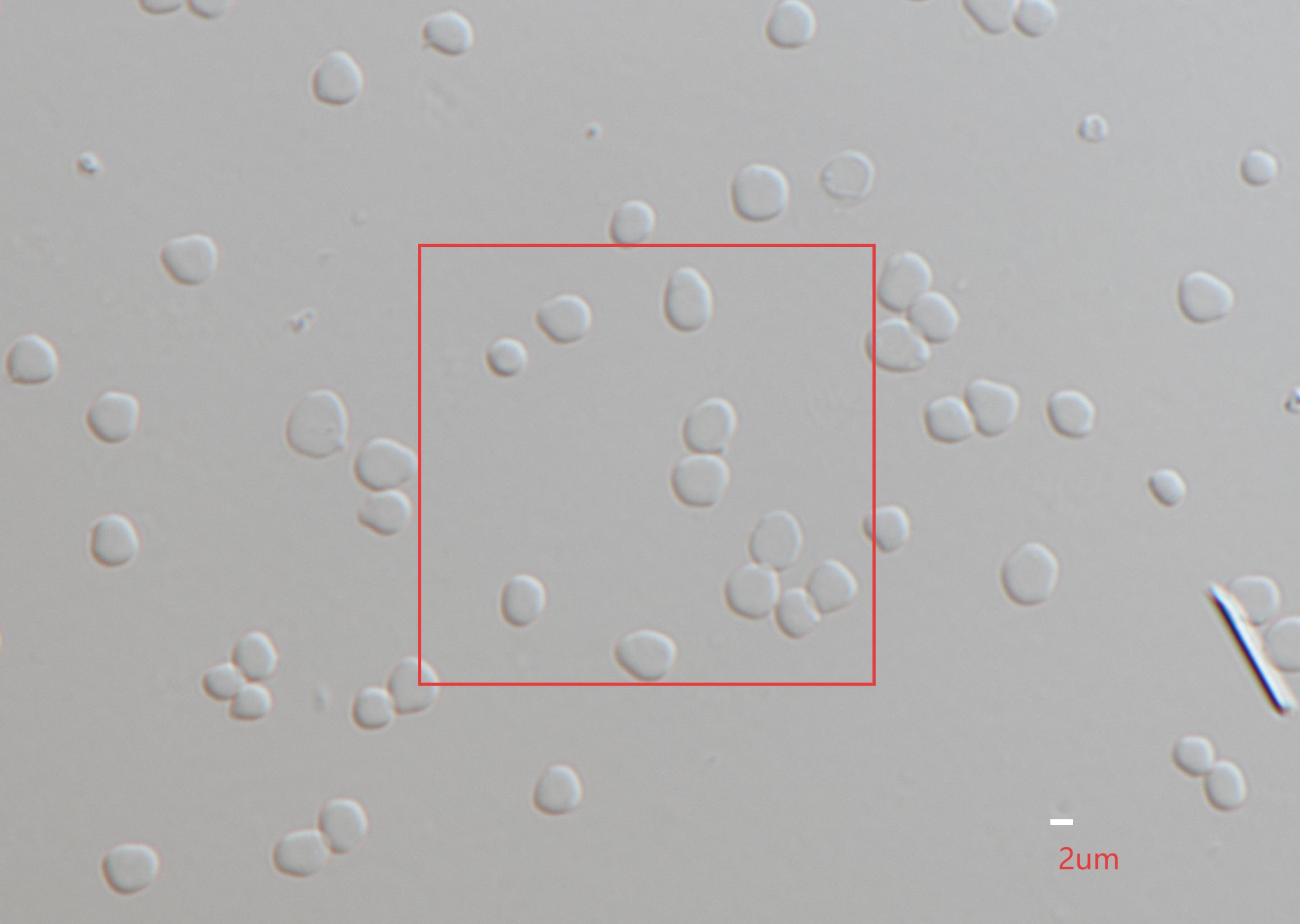

Supplement: Supplementary file 9 — Source data Fig. 6 [file 44319_2025_650_MOESM9_ESM.zip › figure 6B 12h.jpg]

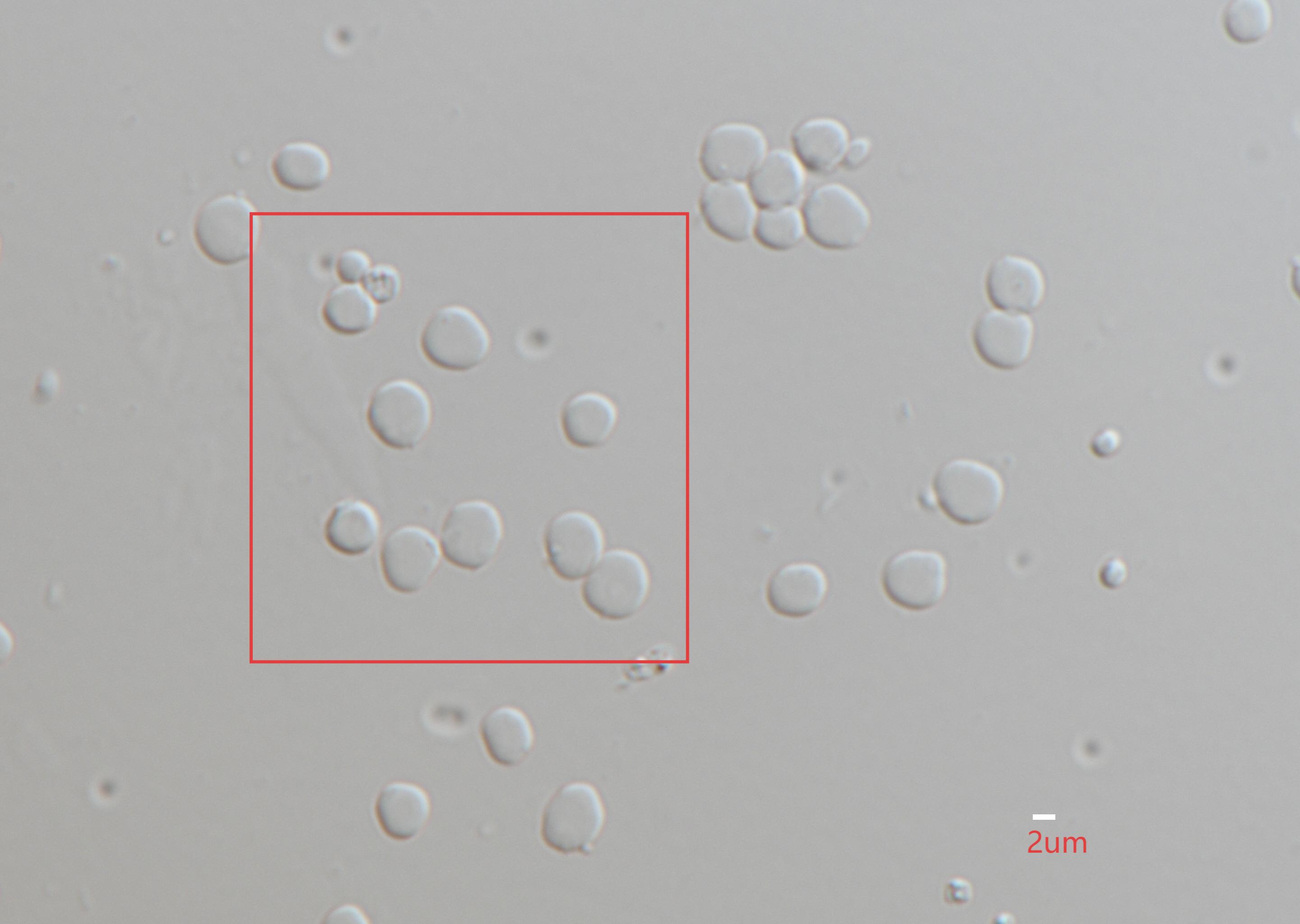

Supplement: Supplementary file 9 — Source data Fig. 6 [file 44319_2025_650_MOESM9_ESM.zip › figure 6B 24h.jpg]

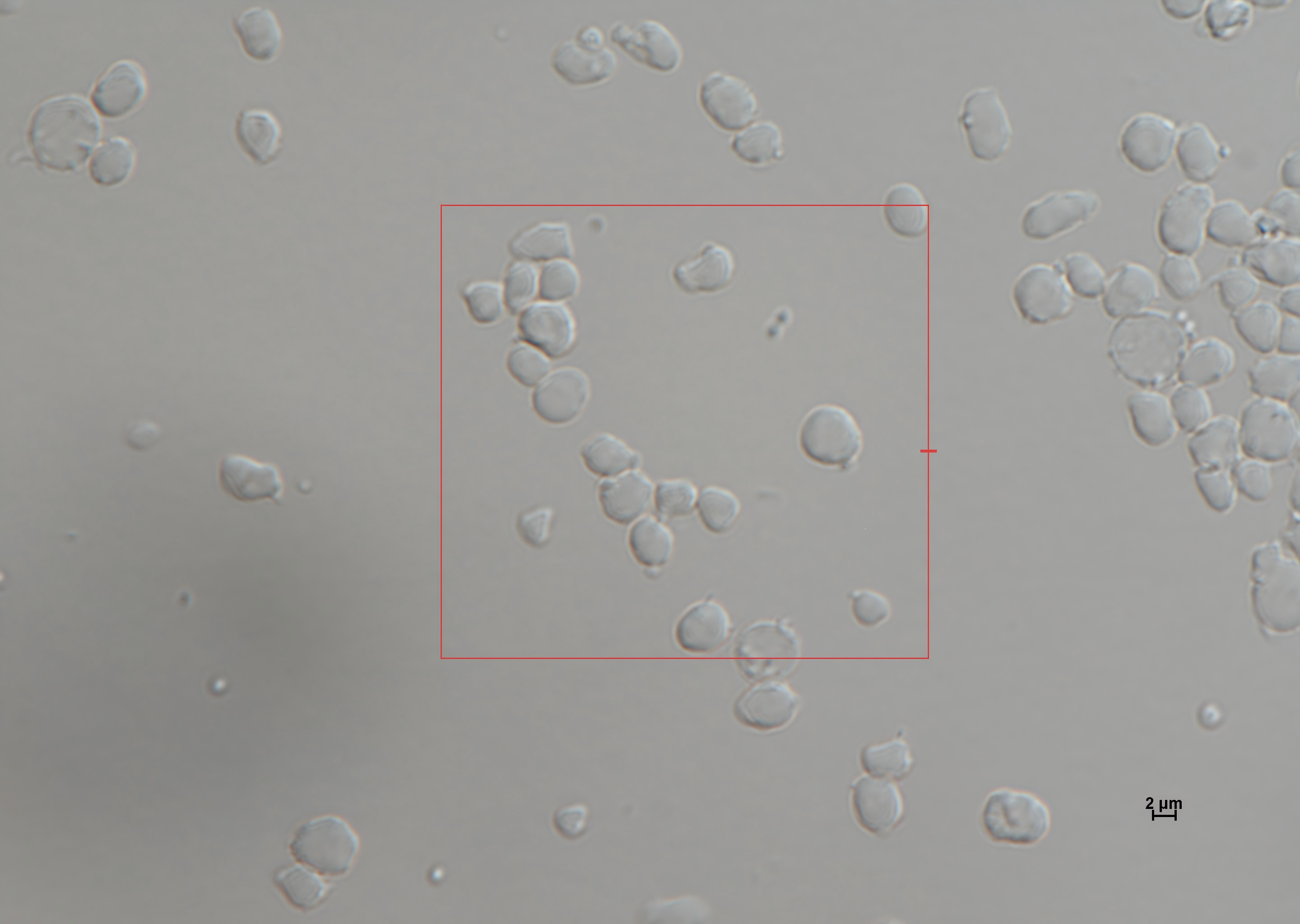

Supplement: Supplementary file 10 — Source data Fig. 7 [file 44319_2025_650_MOESM10_ESM.zip › Figure 7E E246A.png]

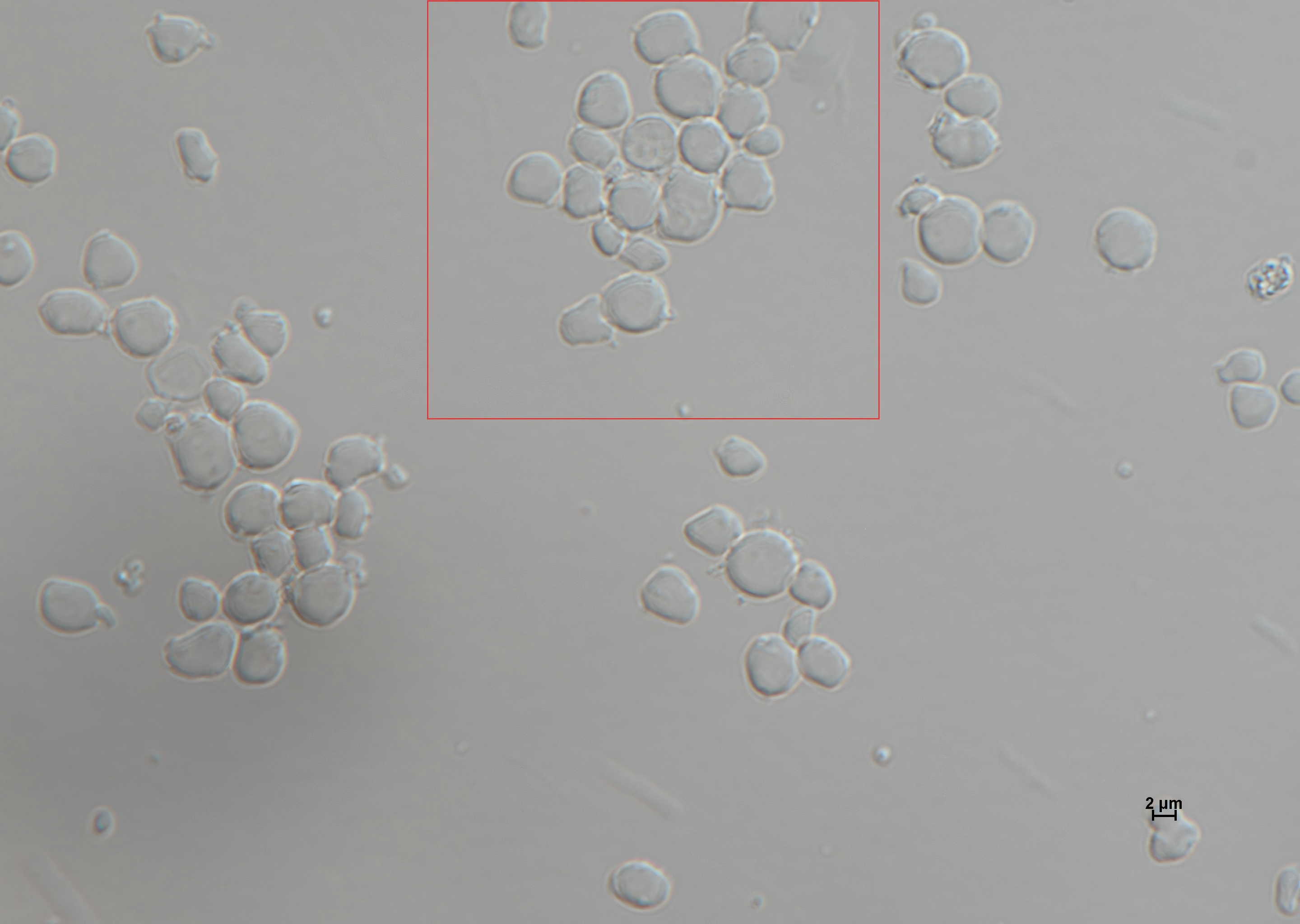

Supplement: Supplementary file 10 — Source data Fig. 7 [file 44319_2025_650_MOESM10_ESM.zip › Figure 7E E247A.png]

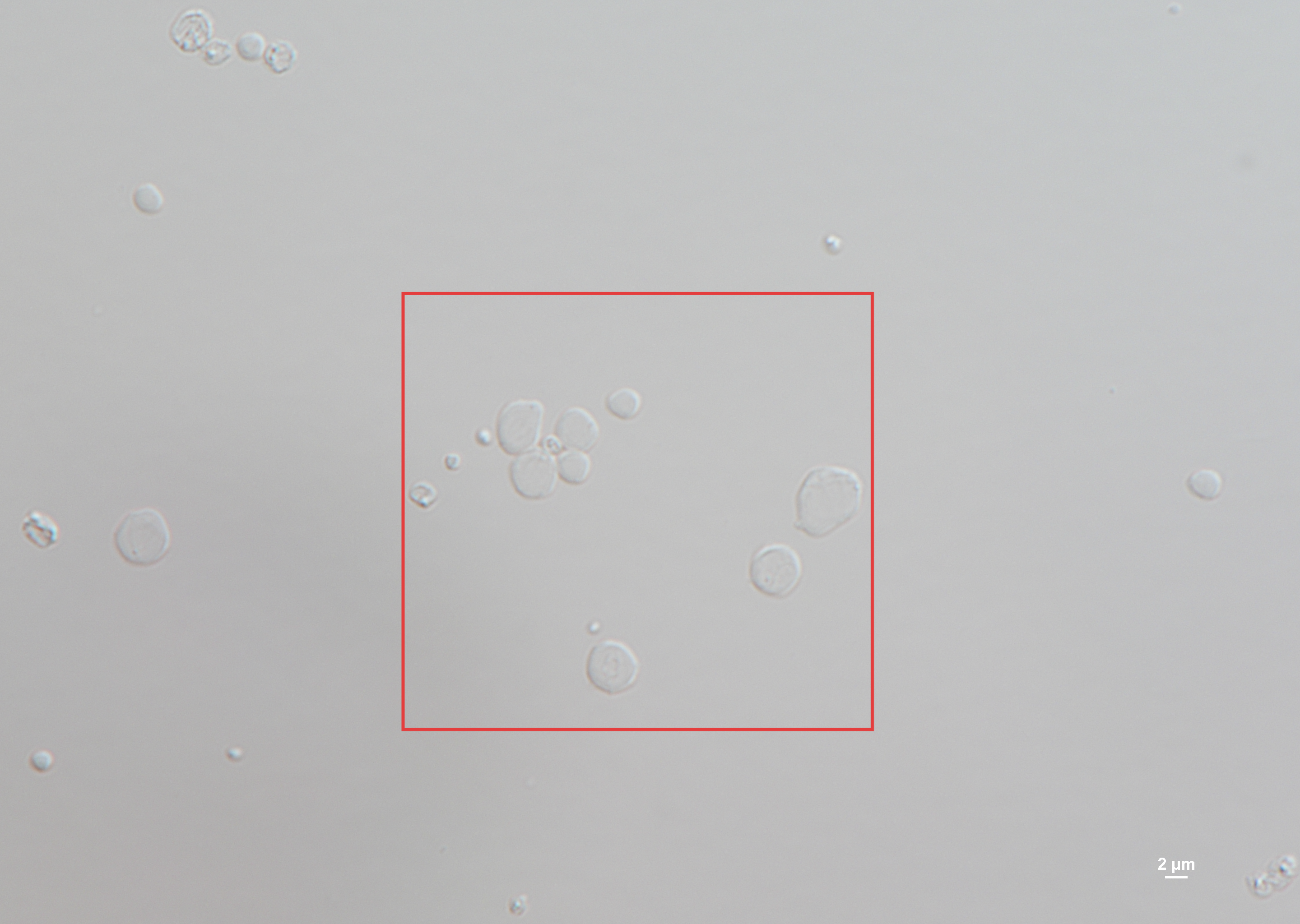

Supplement: Supplementary file 10 — Source data Fig. 7 [file 44319_2025_650_MOESM10_ESM.zip › Figure 7D 2A2B.png]

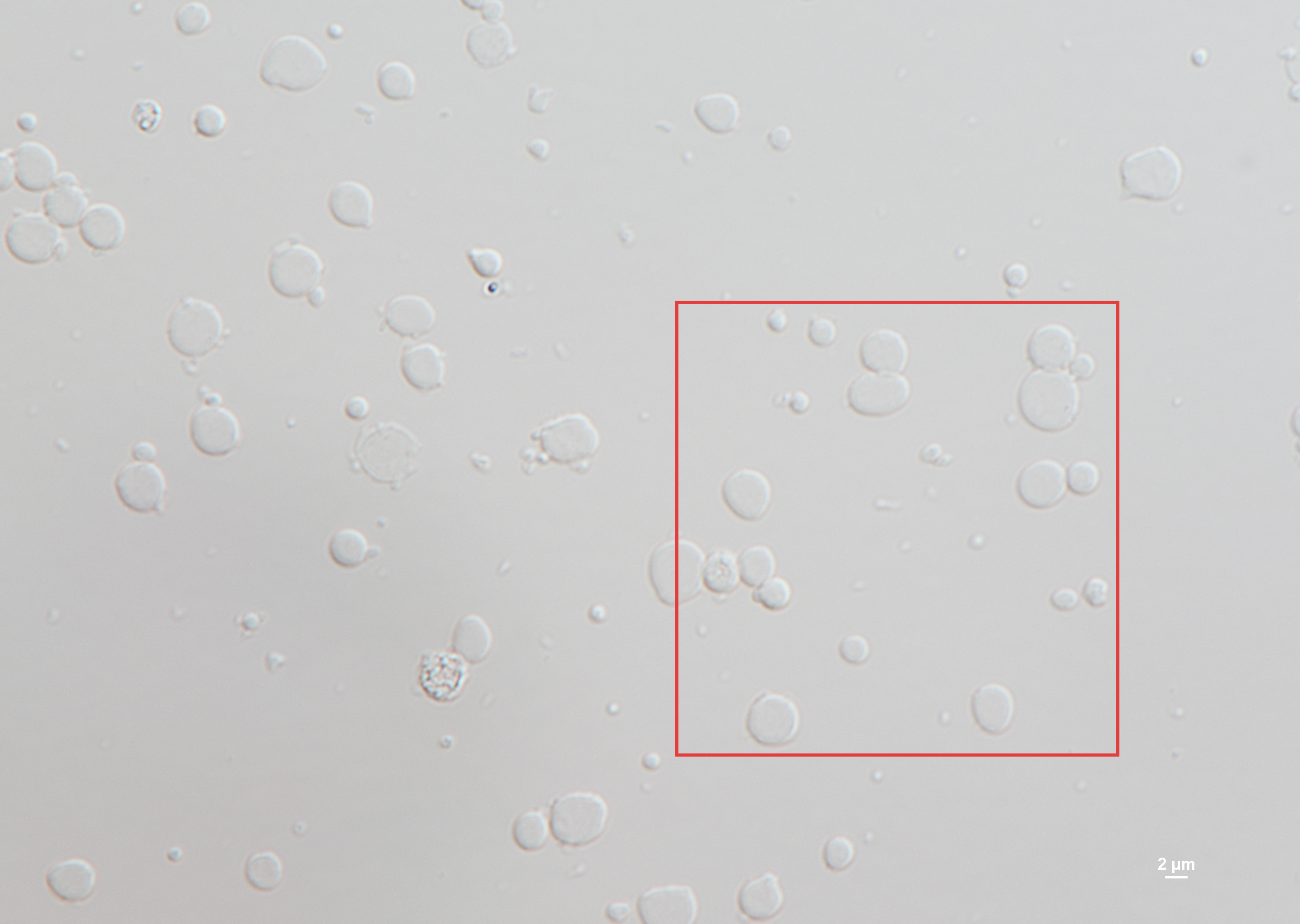

Supplement: Supplementary file 10 — Source data Fig. 7 [file 44319_2025_650_MOESM10_ESM.zip › Figure 7D 3A.png]

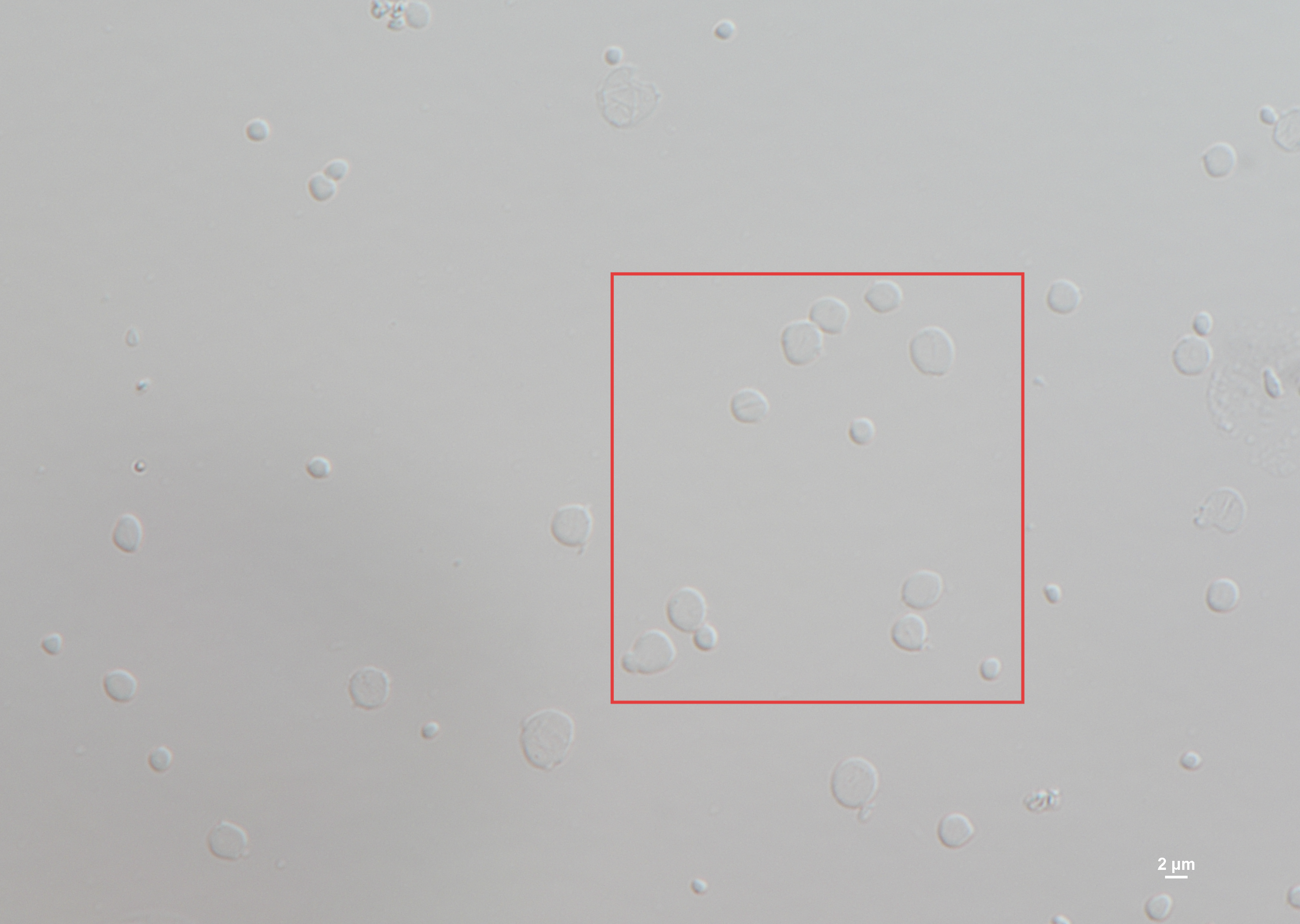

Supplement: Supplementary file 10 — Source data Fig. 7 [file 44319_2025_650_MOESM10_ESM.zip › Figure 7D 3B.png]

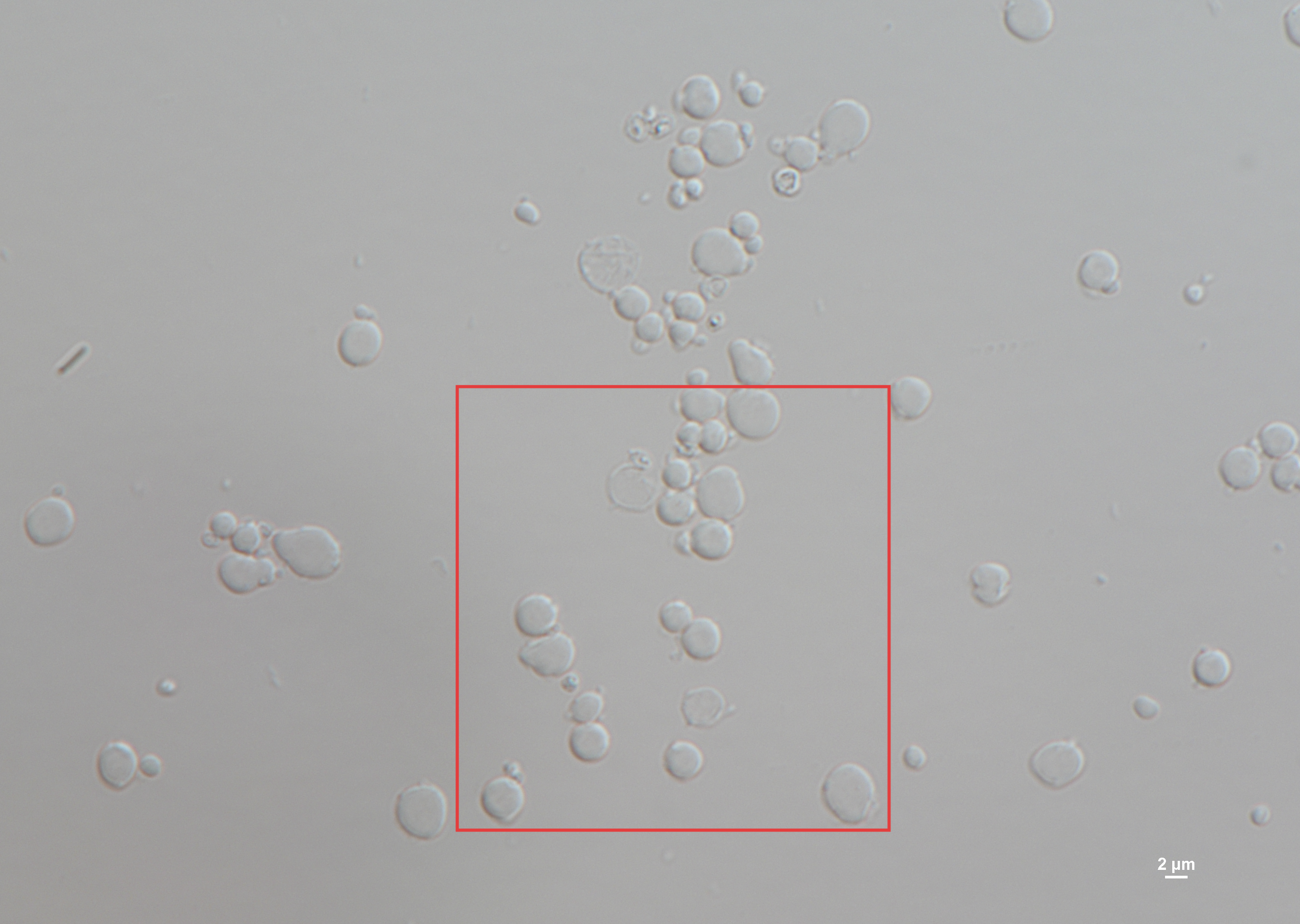

Supplement: Supplementary file 10 — Source data Fig. 7 [file 44319_2025_650_MOESM10_ESM.zip › Figure 7D WT.png]

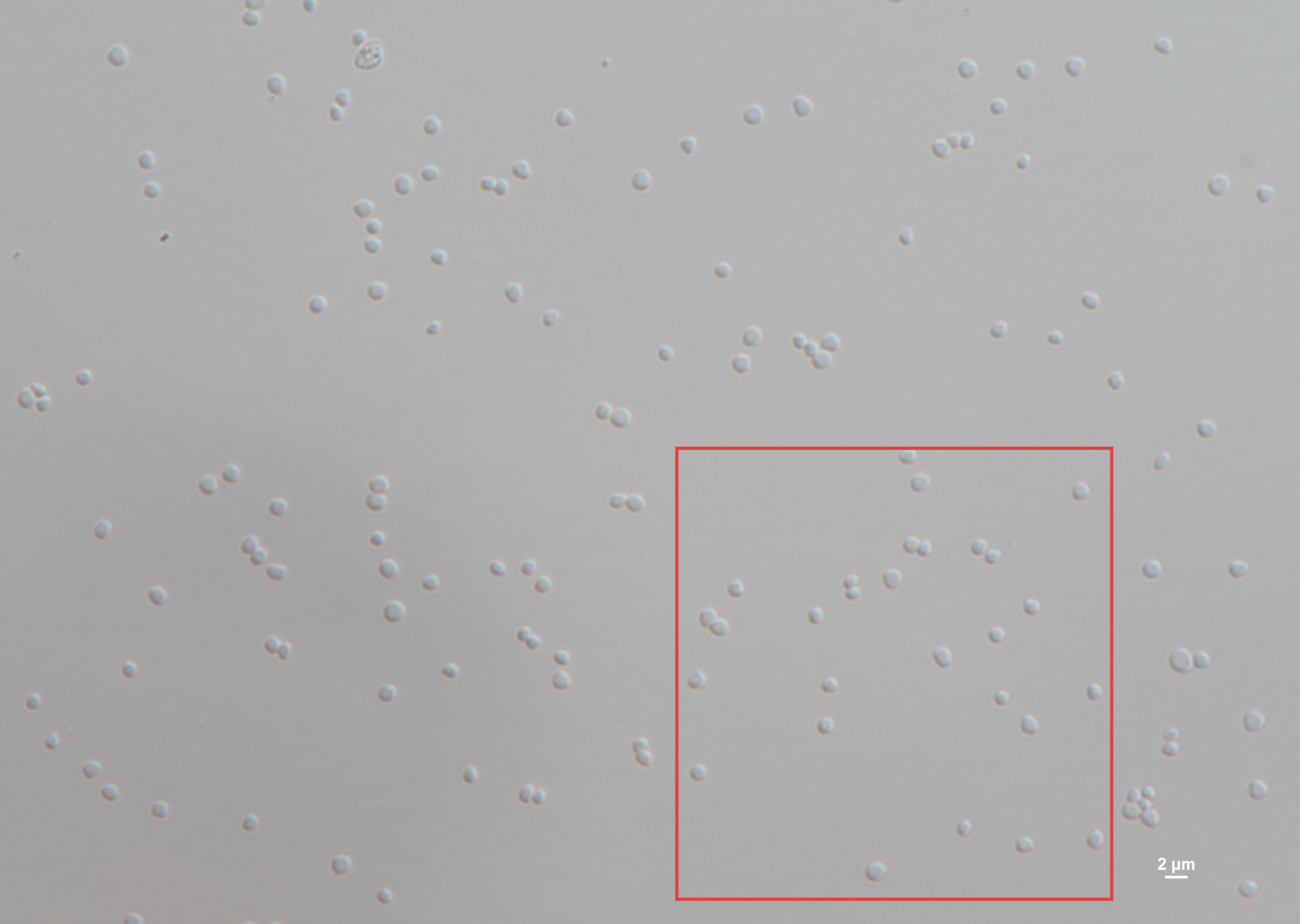

Supplement: Supplementary file 10 — Source data Fig. 7 [file 44319_2025_650_MOESM10_ESM.zip › Figure 7D cont.png]

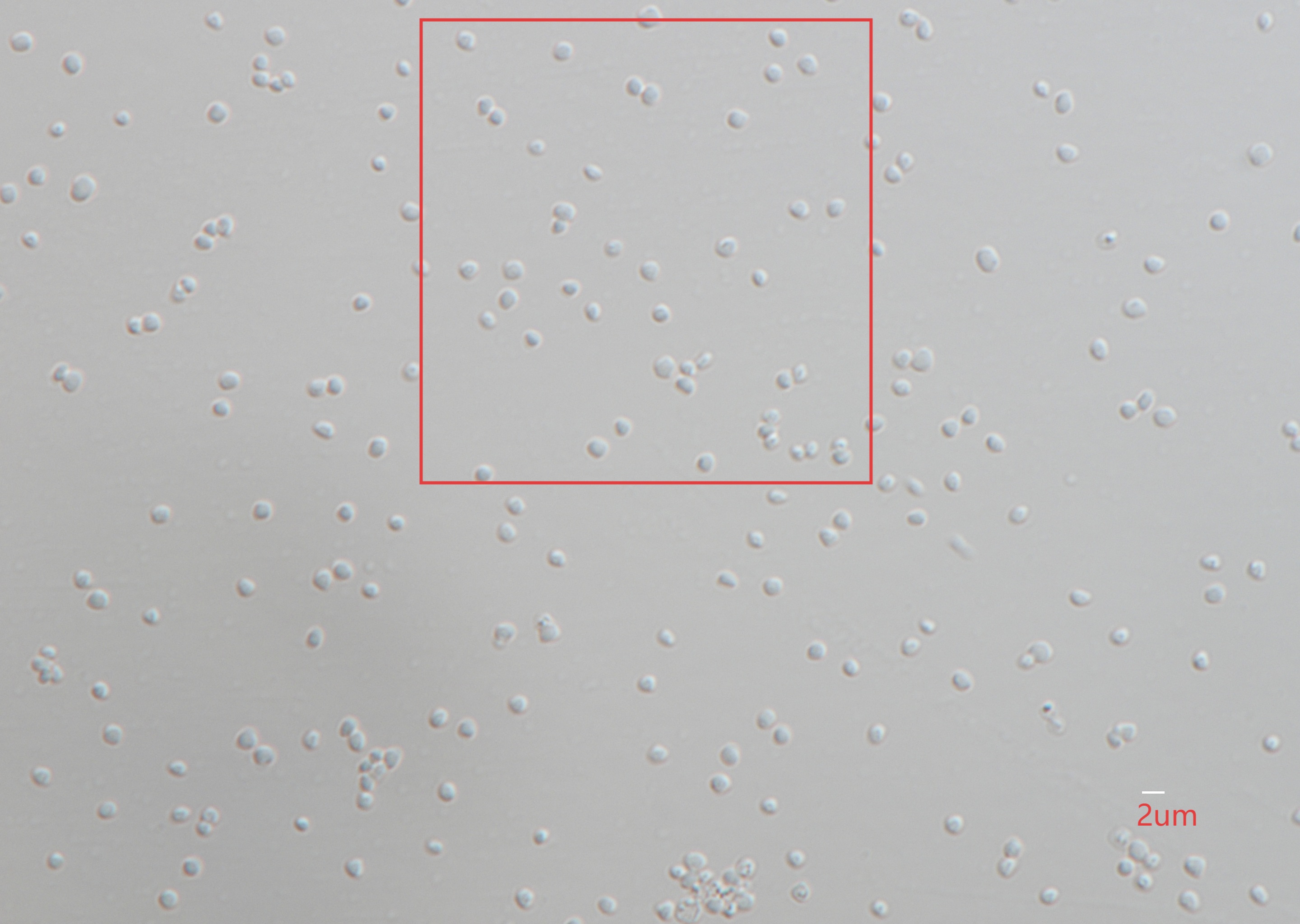

Supplement: Supplementary file 10 — Source data Fig. 7 [file 44319_2025_650_MOESM10_ESM.zip › Figure 7E 37 280 281A.png]

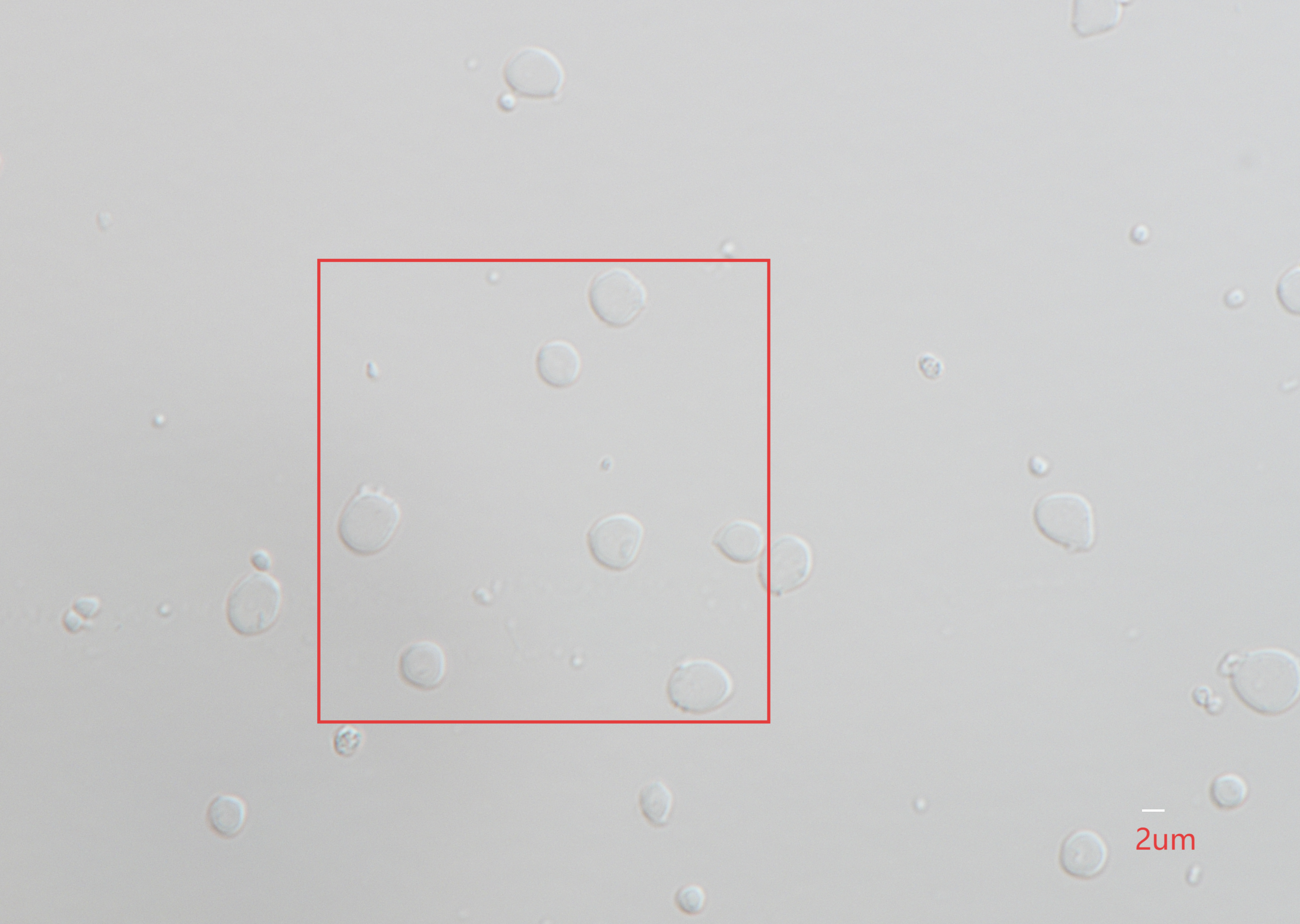

Supplement: Supplementary file 10 — Source data Fig. 7 [file 44319_2025_650_MOESM10_ESM.zip › Figure 7E D157A.png]

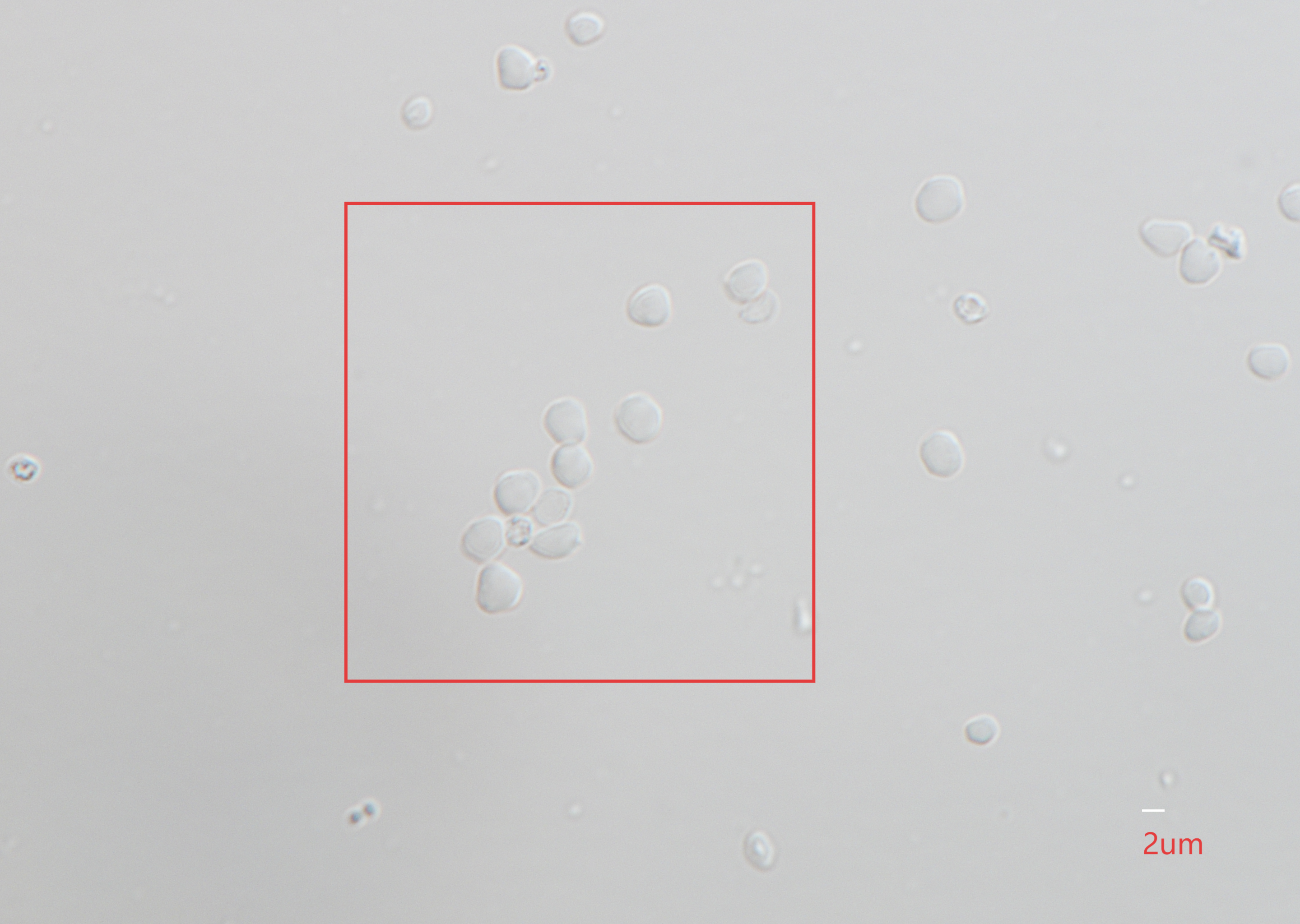

Supplement: Supplementary file 10 — Source data Fig. 7 [file 44319_2025_650_MOESM10_ESM.zip › Figure 7E E249A.png]

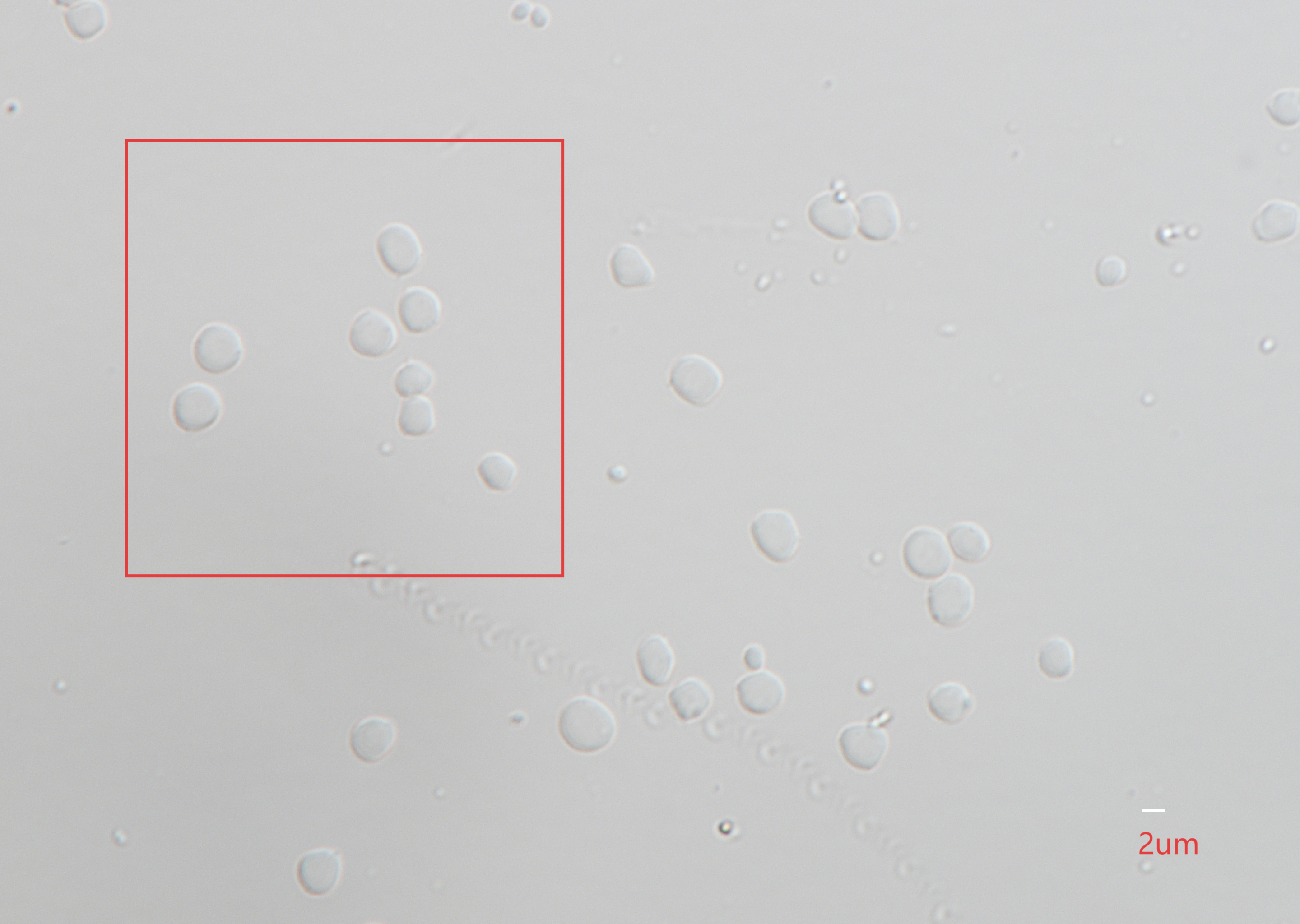

Supplement: Supplementary file 10 — Source data Fig. 7 [file 44319_2025_650_MOESM10_ESM.zip › Figure 7E F156A.png]

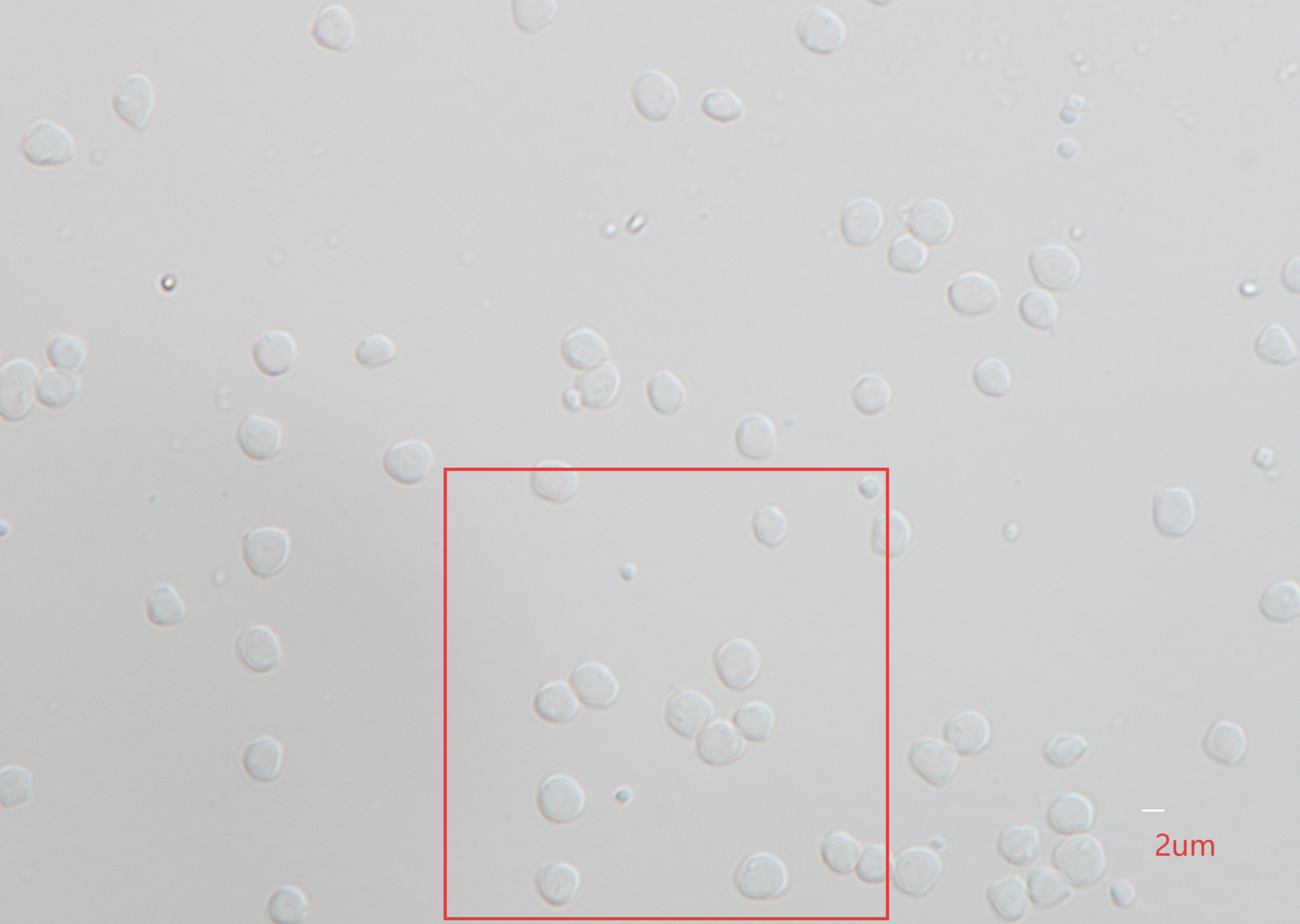

Supplement: Supplementary file 10 — Source data Fig. 7 [file 44319_2025_650_MOESM10_ESM.zip › Figure 7E H280A.png]

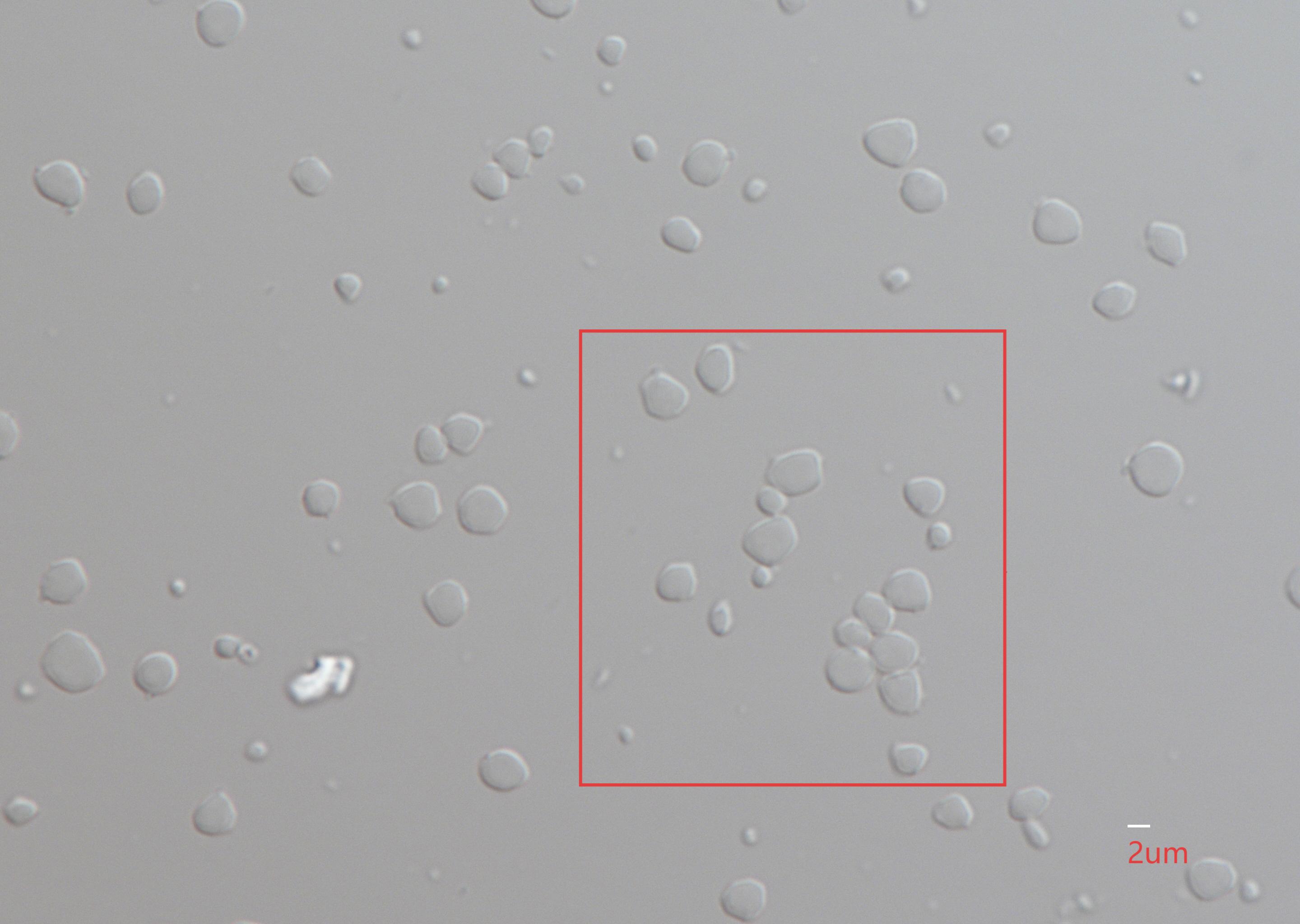

Supplement: Supplementary file 10 — Source data Fig. 7 [file 44319_2025_650_MOESM10_ESM.zip › Figure 7E K36A.jpg]

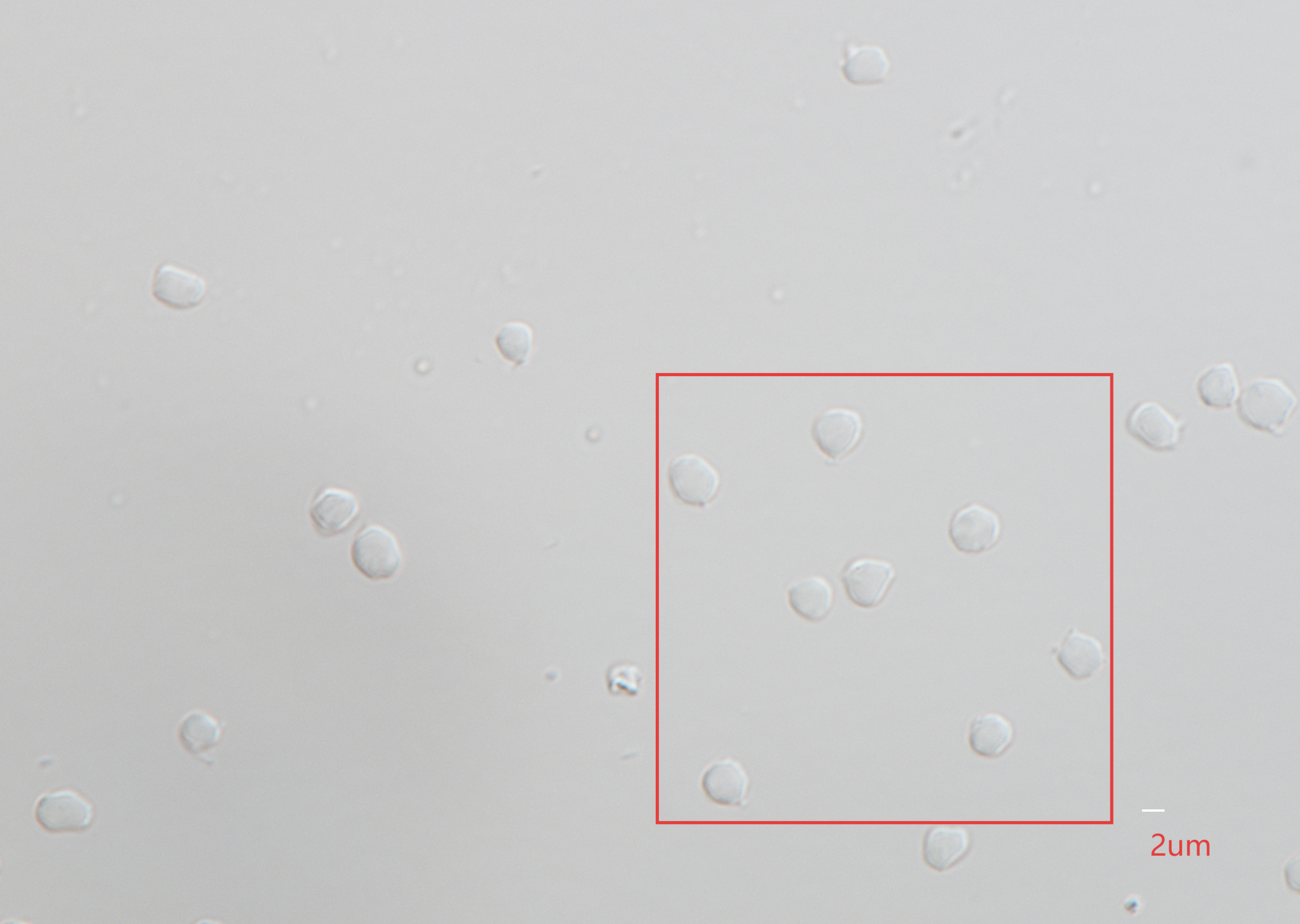

Supplement: Supplementary file 10 — Source data Fig. 7 [file 44319_2025_650_MOESM10_ESM.zip › Figure 7E P248A.png]

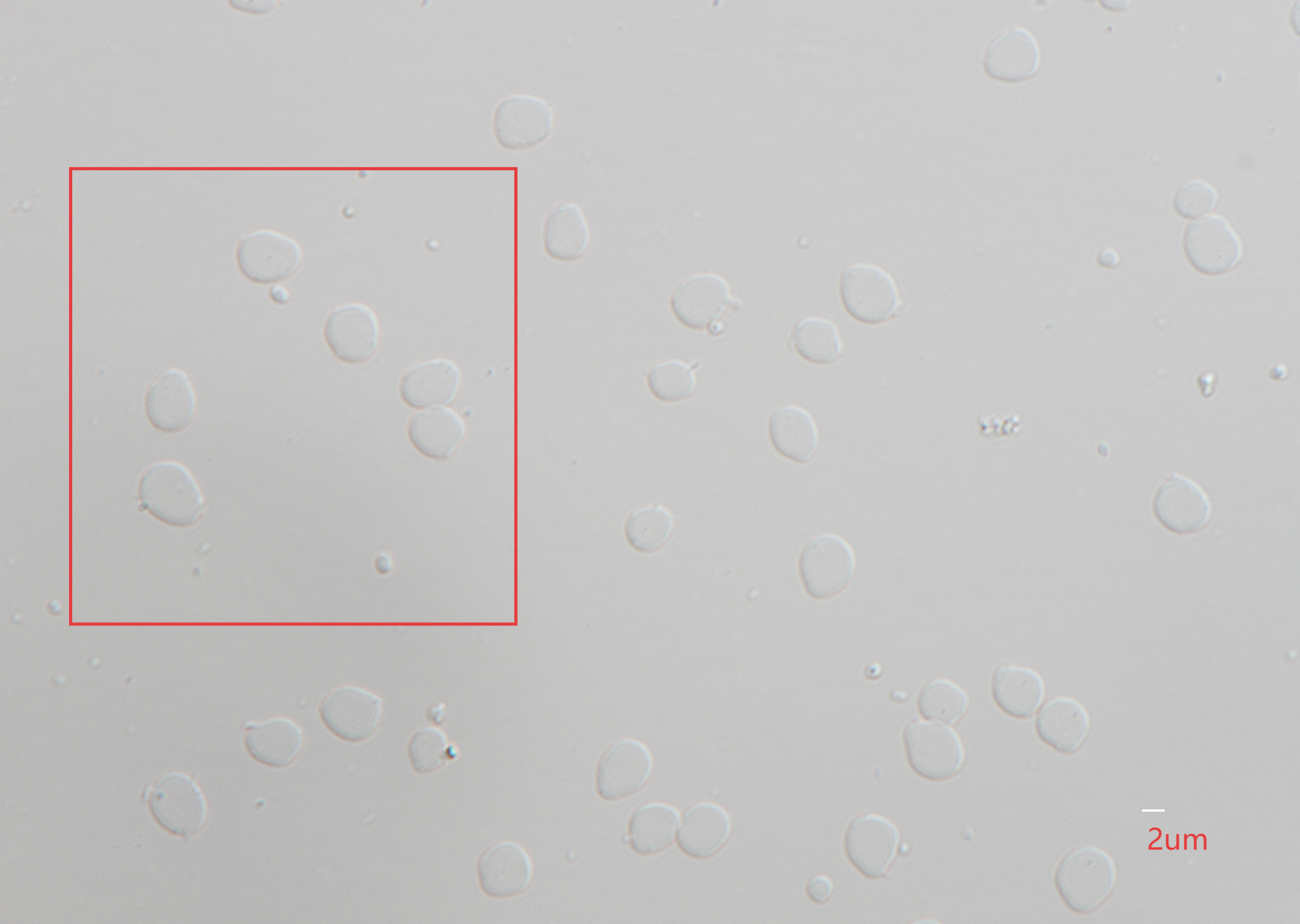

Supplement: Supplementary file 10 — Source data Fig. 7 [file 44319_2025_650_MOESM10_ESM.zip › Figure 7E Q158A.png]

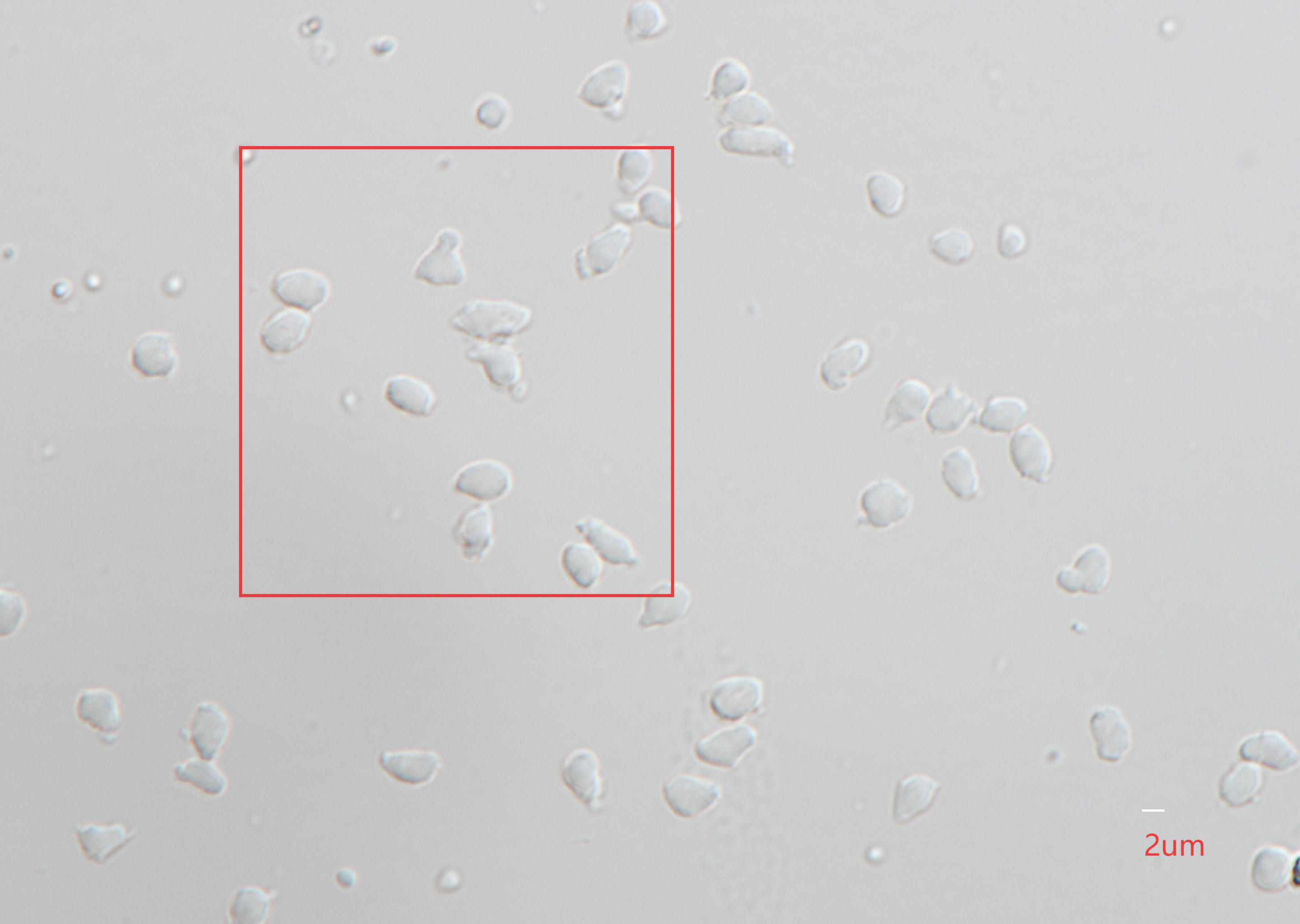

Supplement: Supplementary file 10 — Source data Fig. 7 [file 44319_2025_650_MOESM10_ESM.zip › Figure 7E S281A.png]

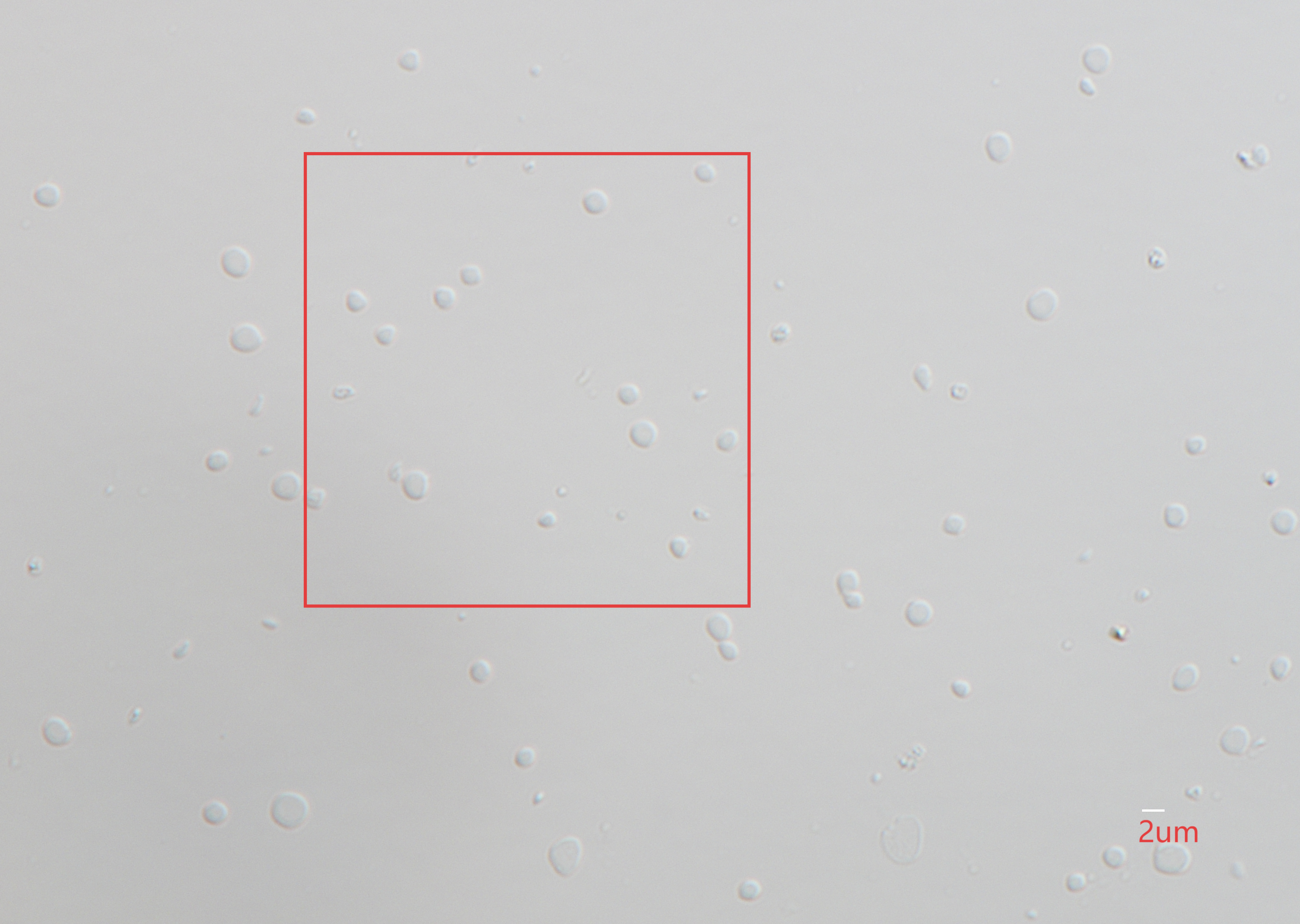

Supplement: Supplementary file 10 — Source data Fig. 7 [file 44319_2025_650_MOESM10_ESM.zip › Figure 7E T37A.png]

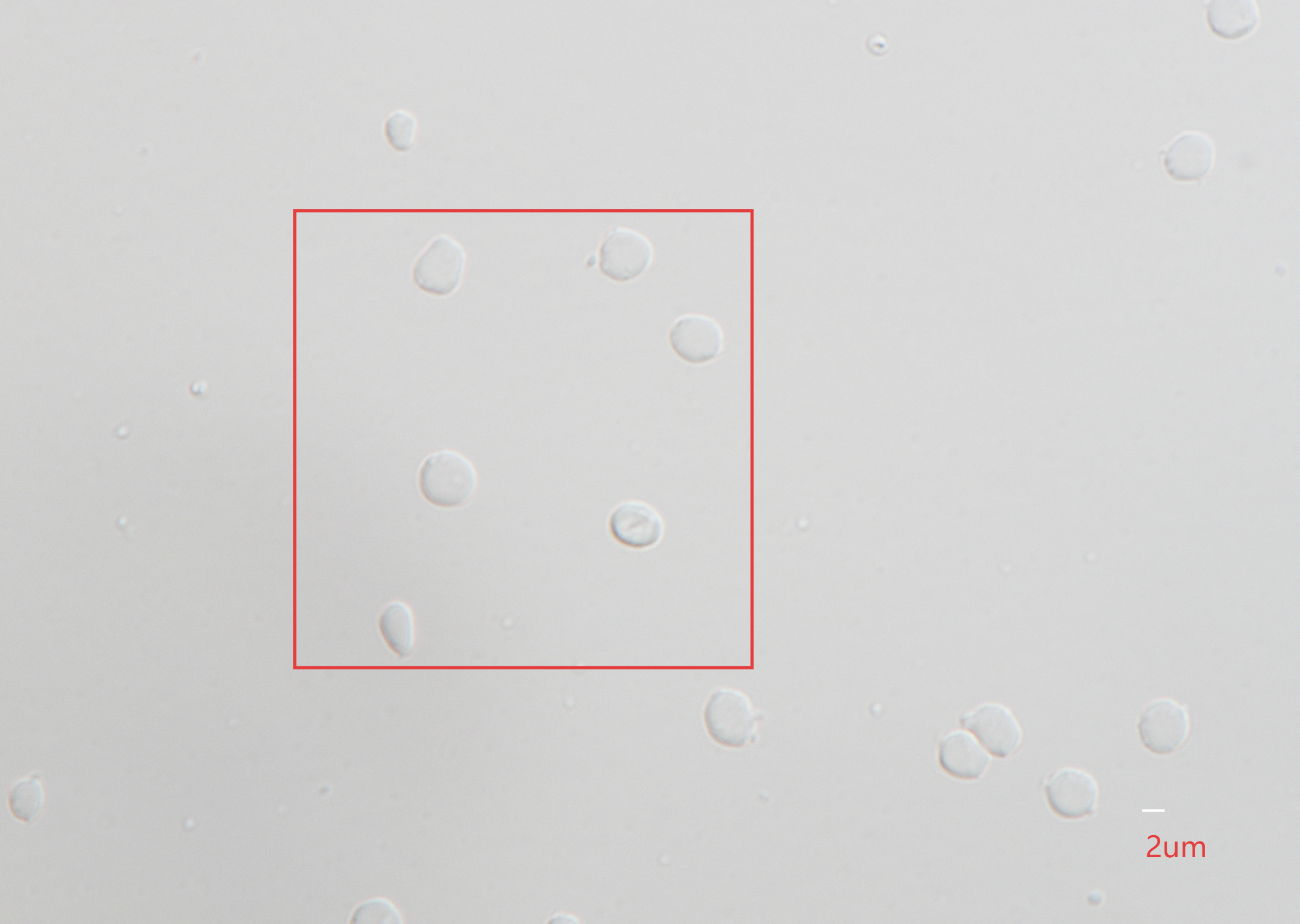

Supplement: Supplementary file 10 — Source data Fig. 7 [file 44319_2025_650_MOESM10_ESM.zip › Figure 7E WT.png]

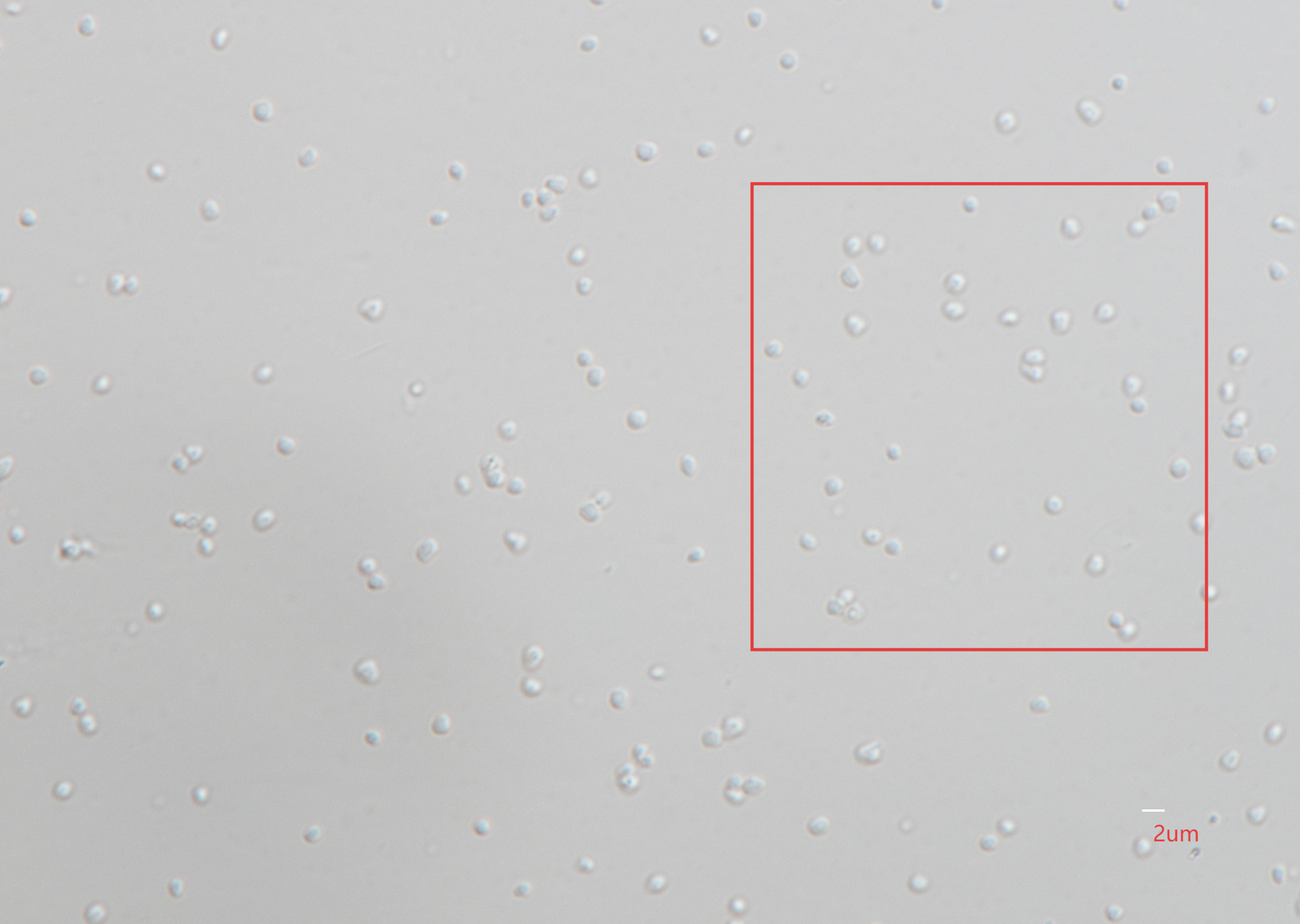

Supplement: Supplementary file 10 — Source data Fig. 7 [file 44319_2025_650_MOESM10_ESM.zip › Figure 7E cont.png]

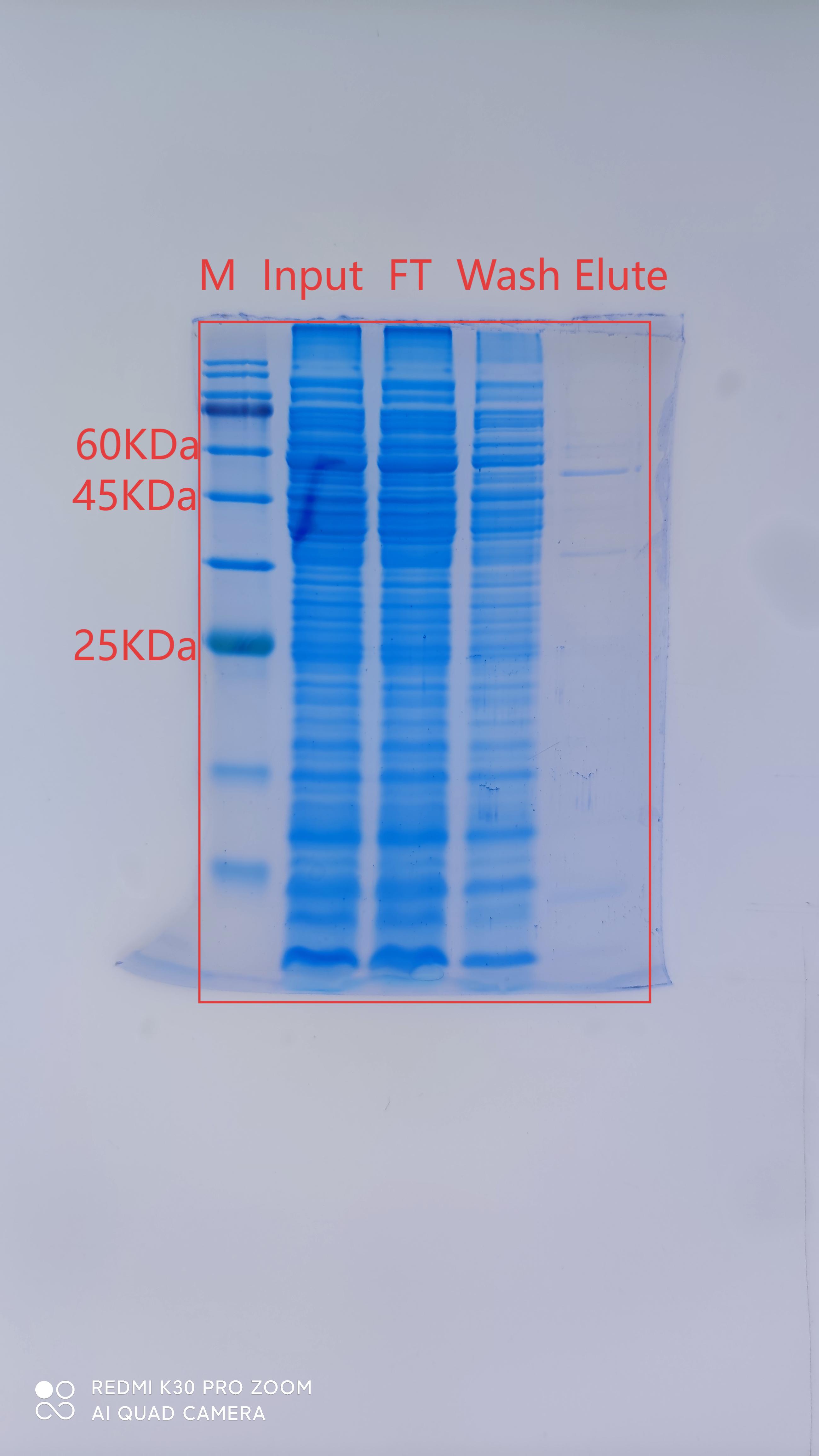

Supplement: Supplementary file 11 — Source data Fig. 8 [file 44319_2025_650_MOESM11_ESM.zip › Figure 8B.jpg]

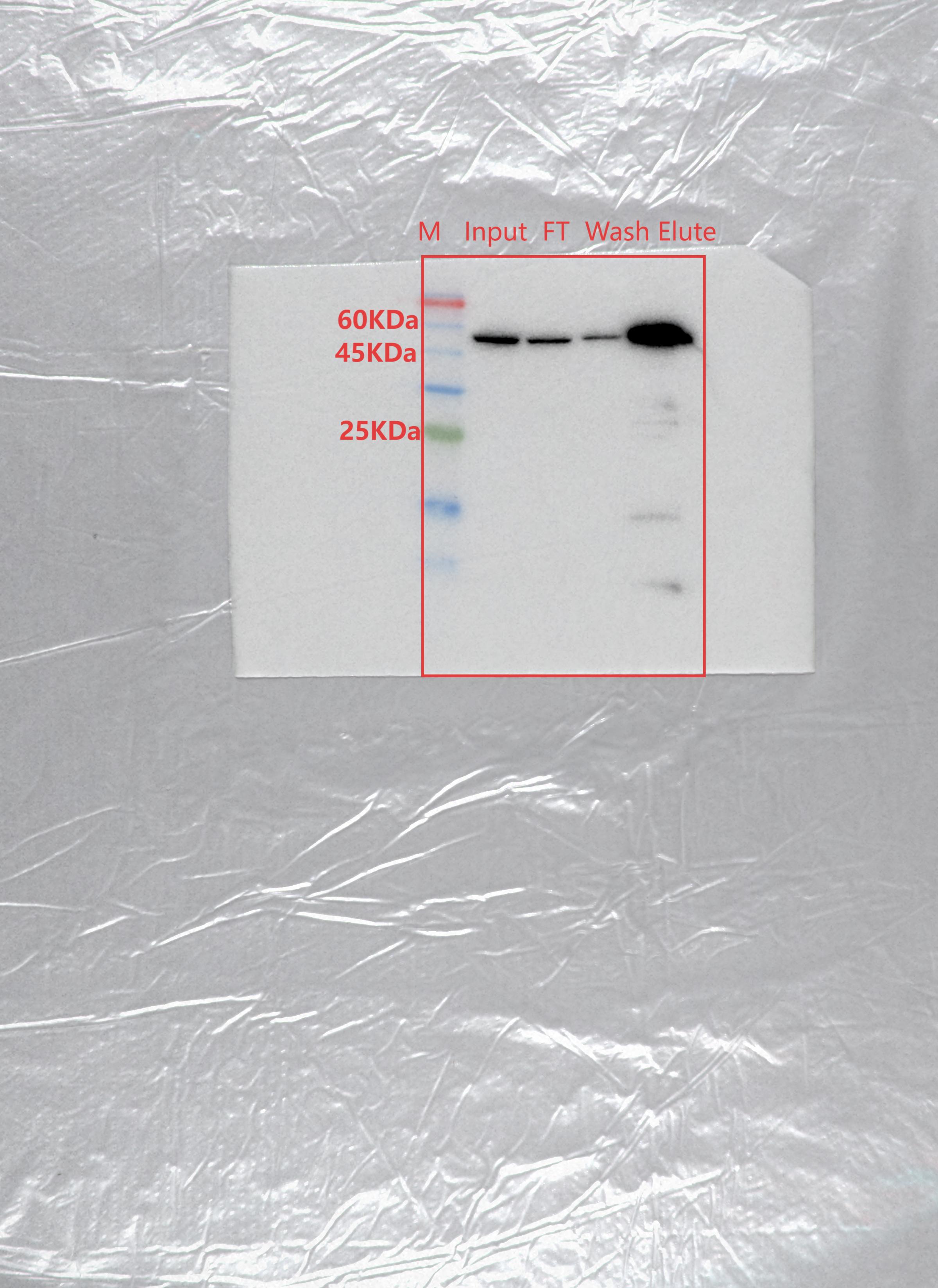

Supplement: Supplementary file 11 — Source data Fig. 8 [file 44319_2025_650_MOESM11_ESM.zip › Figure 8C.jpg]

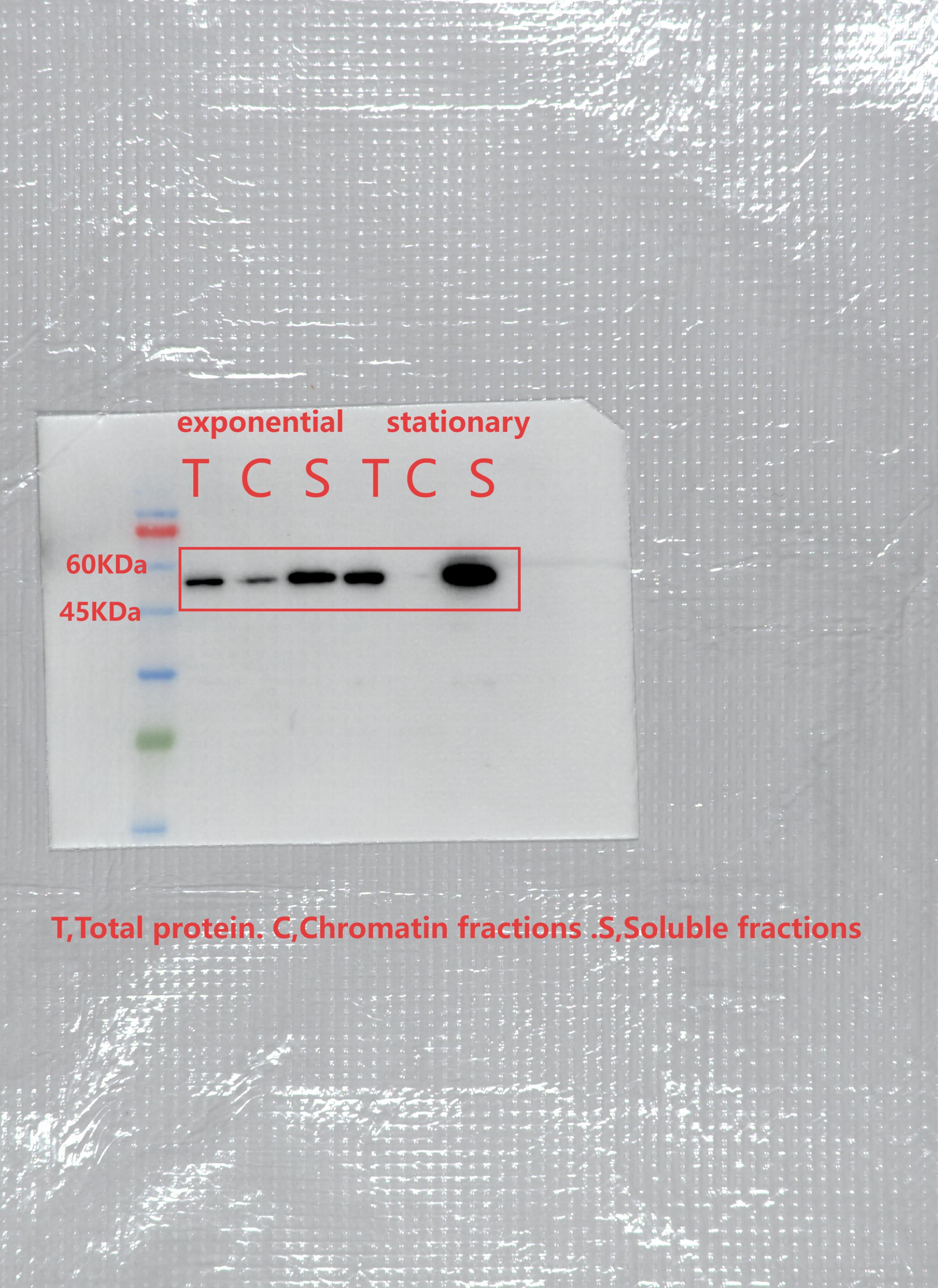

Supplement: Supplementary file 11 — Source data Fig. 8 [file 44319_2025_650_MOESM11_ESM.zip › Figure 8A.jpg]
