# Supplementary material for: Cran1, member of a new class of OLD family ATPases, functions in cell cycle progression in an archaeon
Source: EMBO Rep. 2025 Dec 2;27(1):208–29. doi: 10.1038/s44319-025-00650-y (PMC12796447; doi:10.1038/s44319-025-00650-y)
Supplement: Supplementary file 13 — Expanded View Figures [file 44319_2025_650_MOESM13_ESM.pdf]

# Expanded View Figures

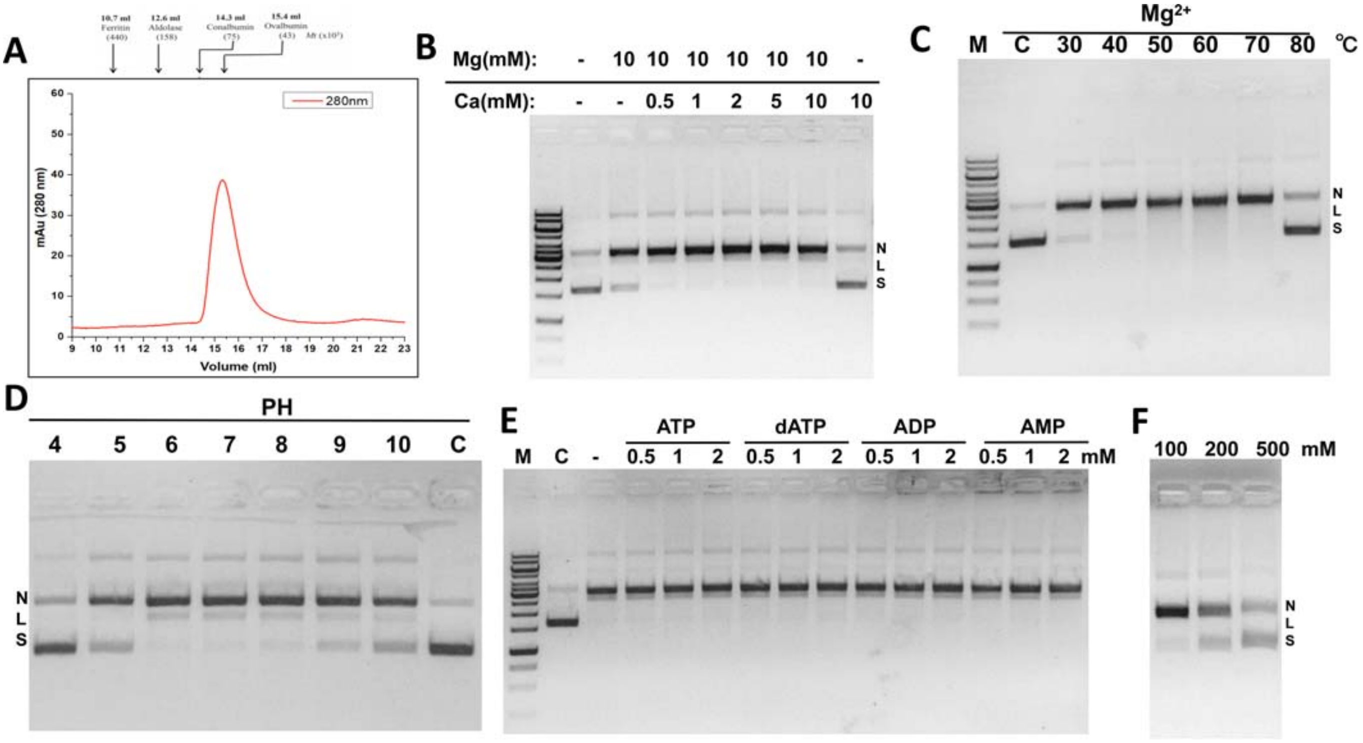


**Figure EV1. Biochemical properties of Cran1 in vitro.**

(**A**) Size exclusion profile of the purified wild-type Cran1. The protein was expressed in *E. coli* BL21 and purified by heat treatment, nickel affinity, and gel filtration with a Superdex 200 column as described in the “Methods”. Cran1 was assayed for nickase activity under different temperature gradient (**B**) as well as PH gradient conditions (**C**). C refers to the control reaction without Cran1 at pH 7 and 37 °C. In these reactions, 300 ng of PUC19 DNA was incubated with 2 µM Cran1 in a final volume of 20 µl. Reactions were performed at 70 °C for 30 min and then stopped by the addition of 4 µl of 6×loading dye containing 20 mM EDTA. Samples were analyzed via native agarose gel electrophoresis. (**D**) Gradual increase in the concentration of calcium ions under 10 mM magnesium ions to observe the effect of two-metal ion-catalyzed conditions on the activity of Cran1 nickase. (**E**) Observation of the effect of 0.5, 1, and 2 mM of ATP, dATP, AMP, and ADP on the activity of Cran1 nickase. (**F**) Effect of KCl at 100, 200, and 500 mM on Cran1 activity. ‘N’, ‘L’, and ‘S’ denote the positions of ‘nicked’, ‘linearized’, and ‘supercoiled’ DNA, respectively. Source data are available online for this figure.

© The Author(s) 

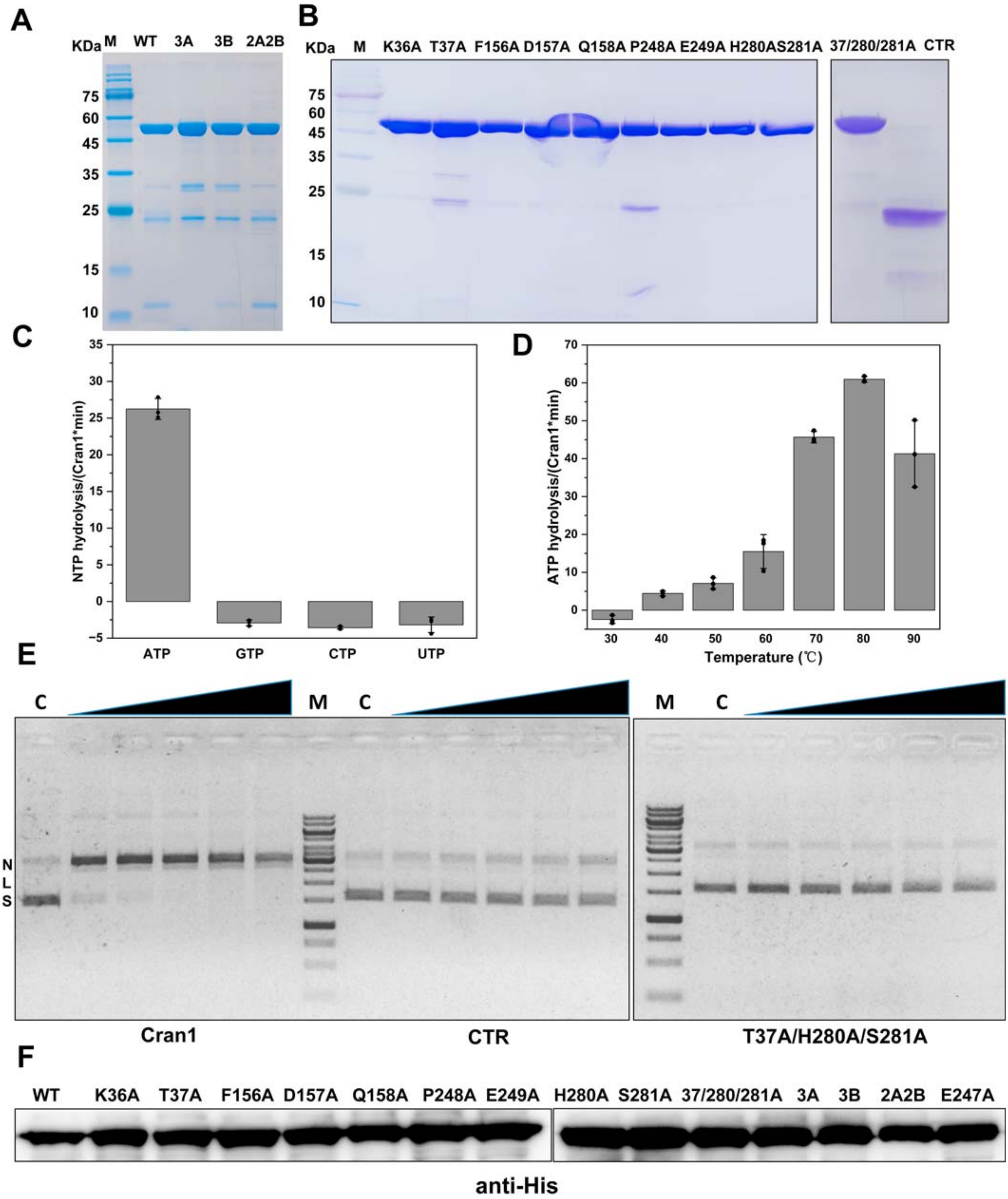
A
KDa M WT 3A 3B 2A2B
75
60
45
35
25
15
10
B
KDa M K36A T37A F156A D157A Q158A P248A E249A H280A S281A 37/280/281A CTR
75
60
45
35
25
15
10
C
NTP hydrolysis/(Cran1*min)
ATP GTP CTP UTP
D
ATP hydrolysis/(Cran1*min)
Temperature (℃)
30 40 50 60 70 80 90
E
C M C M C
N
L
S
Cran1
CTR
T37A/H280A/S281A
F
WT K36A T37A F156A D157A Q158A P248A E249A H280A S281A 37/280/281A 3A 3B 2A2B E247A
anti-His

**Figure EV2. Analysis of the activities of the wild-type and the mutant proteins of Cran1.**

(**A**, **B**) SDS-PAGE analysis of the wild-type, nuclease active site mutants (**A**) and the ATPase active site mutants (**B**) of Cran1. (**C**) Assay of the ability to hydrolyze ATP, GTP, CTP, or UTP. Data from three independent replicates were shown as mean ± SD. Error bars represent standard deviations from three independent measurements. Source data are available online for this figure. (**D**) The hydrolytic activity of Cran1 on ATP under different temperature conditions. Data from three independent replicates were shown as mean ± SD. Error bars represent standard deviations from three independent measurements. Source data are available online for this figure. (**E**) The ATPase activity of Cran1 is essential for the performance of nuclease functions. The cleavage experiments of wild-type Cran1 as well as mutant CTR, T37A/H280A/S281A were carried out under the same conditions as in the previous reaction, with magnesium ion conditions at 70 °C for 30 min, and protein concentration gradients of 0.25, 0.5, 1, 2, and 4 μM. 'N', 'L', and 'S' denote the positions of 'nicked', 'linearized', and 'supercoiled' DNA, respectively. (**F**) Analysis of the protein levels in cells overexpressing the wild-type Cran1 and its mutant by Western blotting using anti-His tag antibody. Source data are available online for this figure.

© The Author(s)

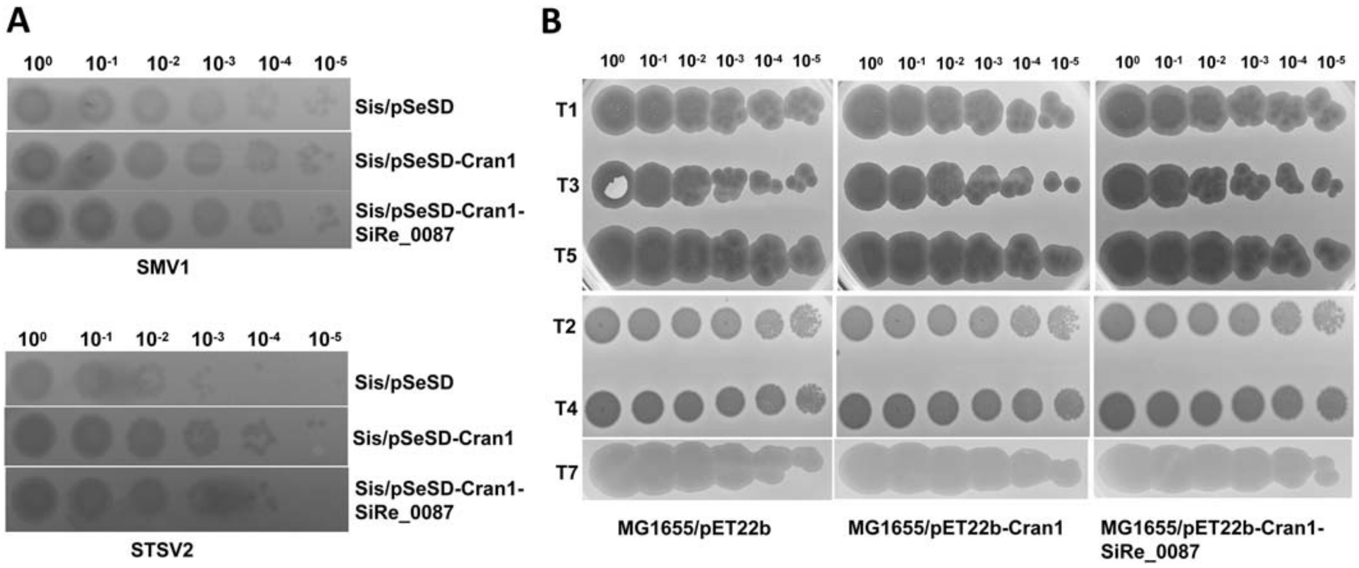


**Figure EV3. Cran1 is not involved in virus defense.**

(**A**) Spot assay with the *Sa. islandicus* virus SMV1 and STSV2 infecting the cells overexpressing Cran1 and cells co-expressing Cran1 and SiRe_0087. (**B**) Spot assay of the infection of phage T1, T2, T3, T4, T5, T7 on *Escherichia coli* MG1655 cells expressing Cran1 or co-expressing Cran1 and SiRe_0087. Both archaea virus and bacterial phages were diluted in a tenfold gradient. Cells carrying the empty vectors pSeSD and pET22b were used as controls. Source data are available online for this figure.

 © The Author(s)

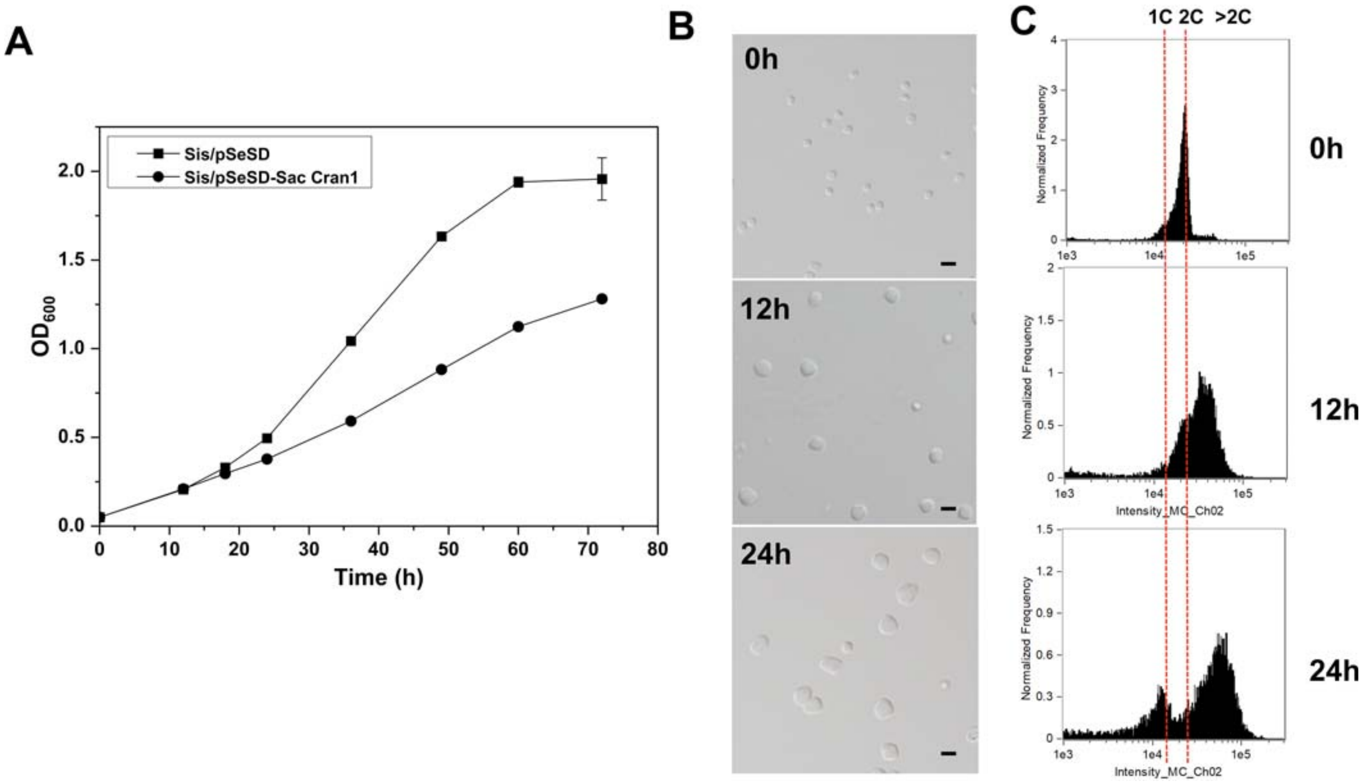


**Figure EV4. Cells with overexpression of the Cran1 homolog from *S. acidocaldarius* (Saci_0157, SacCran1) has similar phenotypes as those of Cran1.**

(**A**) Growth curves of strain Sis/pSeSD-Saci_0157. The cells were inoculated into 30 ml ATV medium to a final estimated $OD_{600}$ of 0.03 and the growth was monitored using spectrometer. Each value was based on data from three independent repeats. Cell harboring the empty plasmid pSeSD was used as a control. (**B**) Differential interference contrast (DIC) mode microscopy and (**C**) flow cytometry of cells overexpressing SacCran1. Cells cultured in the induction medium ATV were taken at different time and observed under an inverted fluorescence microscope. DNA content of cells was analyzed using an ImageStreamX MarkII Quantitative imaging analysis flow cytometry (Merck Millipore, Germany). Scale bars: 2 µm. Source data are available online for this figure.

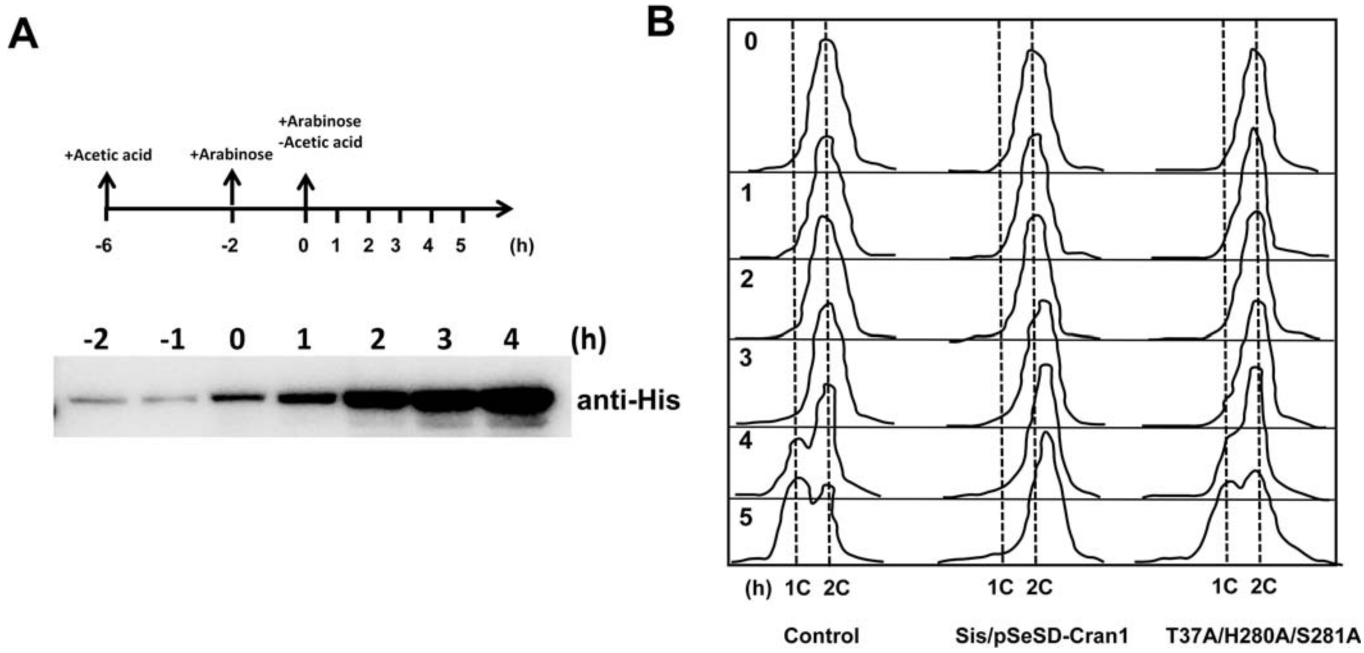


**Figure EV5. The overexpression of Cran1 impairs the cell cycle progression in cells synchronized at the G2 phase.**

(**A**) A schematic showing cell synchronization and induction of Cran1 overexpression with arabinose (0.2%). The time for the acetic acid treatment and arabinose induction are indicated. E233S containing the empty plasmid (Sis/pSeSD) was used as a control. (**B**) Flow cytometry profiles of DNA content distribution of cells 0–5 h after acetic acid removal. Source data are available online for this figure.


© The Author(s)
